# Osteology of *Carnufex carolinensis* (Archosauria: Psuedosuchia) from the Pekin Formation of North Carolina and Its Implications for Early Crocodylomorph Evolution

**DOI:** 10.1371/journal.pone.0157528

**Published:** 2016-06-15

**Authors:** Susan M. Drymala, Lindsay E. Zanno

**Affiliations:** 1 Research & Collections, North Carolina Museum of Natural Sciences, Raleigh, North Carolina, United States of America; 2 Department of Biological Sciences, North Carolina State University, Raleigh, North Carolina, United States of America; College of the Holy Cross, UNITED STATES

## Abstract

Crocodylomorphs originated in the Late Triassic and were the only crocodile-line archosaurs to survive the end-Triassic extinction. Recent phylogenetic analyses suggest that the closest relatives of these generally gracile, small-bodied taxa were a group of robust, large-bodied predators known as rauisuchids implying a problematic morphological gap between early crocodylomorphs and their closest relatives. Here we provide a detailed osteological description of the recently named early diverging crocodylomorph *Carnufex carolinensis* from the Upper Triassic Pekin Formation of North Carolina and assess its phylogenetic position within the Paracrocodylomorpha. *Carnufex* displays a mosaic of crocodylomorph, rauisuchid, and dinosaurian characters, as well as highly laminar cranial elements and vertebrae, ornamented dermal skull bones, a large, subtriangular antorbital fenestra, and a reduced forelimb. A phylogenetic analysis utilizing a comprehensive dataset of early paracrocodylomorphs and including seven new characters and numerous modifications to characters culled from the literature recovers *Carnufex carolinensis* as one of the most basal members of Crocodylomorpha, in a polytomy with two other large bodied taxa (CM 73372 and *Redondavenator*). The analysis also resulted in increased resolution within Crocodylomorpha and a monophyletic clade containing the holotype and two referred specimens of *Hesperosuchus* as well as *Dromicosuchus*. *Carnufex* occupies a key transition at the origin of Crocodylomorpha, indicating that the morphology typifying early crocodylomorphs appeared before the shift to small body size.

## Introduction

Crocodile-line archosaurs (Pseudosuchia) underwent a rapid radiation in the wake of the Permian-Triassic mass extinction and came to dominate terrestrial ecosystems by the Late Triassic [1]. Despite their widespread success, only a single pseudosuchian clade—Crocodylomorpha—survived the end-Triassic extinction event, singlehandedly defining psuedosuchian evolution for the next 200 million years. Earliest known crocodylomorphs were in large part, gracile, small-bodied, terrestrial forms [2]. However, several large-bodied early crocodylomorphs–*Redondavenator quayensis*, CM 73372, a preliminarily reported specimen from the Ischigualasto Formation, and *Carnufex carolinensis*–have been identified recently [3–7], expanding our understanding of the basal crocodylomorph bauplan and raising additional questions regarding the origin and earliest evolution of the clade.

Recent phylogenetic analyses (e.g. [4,8]) recover Rauisuchidae, a group of large-bodied predatory pseudosuchians, as the sister taxon to Crocodylomorpha in a clade dubbed Loricata. This topology produces an absence of transitional morphologies between early crocodylomorphs and their closest relatives [9]. In addition to this sister taxon relationship, there is growing consensus that the loricatan groups “Rauisuchia” (large-bodied suchians including *Postosuchus kirkpatricki* and *Batrachotomus kupferzellensis*) and “Sphenosuchia” (small-bodied, gracile, foxlike crocodylomorphs including *Sphenosuchus acutus* and *Hesperosuchus agilis*) are not clades as currently defined, but rather paraphyletic grades at the base of Loricata and Crocodylomorpha, respectively [2,4,5,8,10–18].

The recently named *Carnufex carolinensis* [6], an archosaur from the Upper Triassic Chatham Group of North Carolina, displays a mosaic of rauisuchid and crocodylomorph characters, which is helping to clarify the earliest evolution of Crocodylomorpha. Here we present a detailed osteology of *Carnufex carolinensis*, explore its phylogenetic context utilizing a novel paracrocodylomorph data set, and consider the evolutionary and ecological implications of large-bodied crocodylomorphs during the transition from large-bodied basal loricatans to small-bodied early crocodylomorphs.

### Geologic Setting

**Fig 1:**
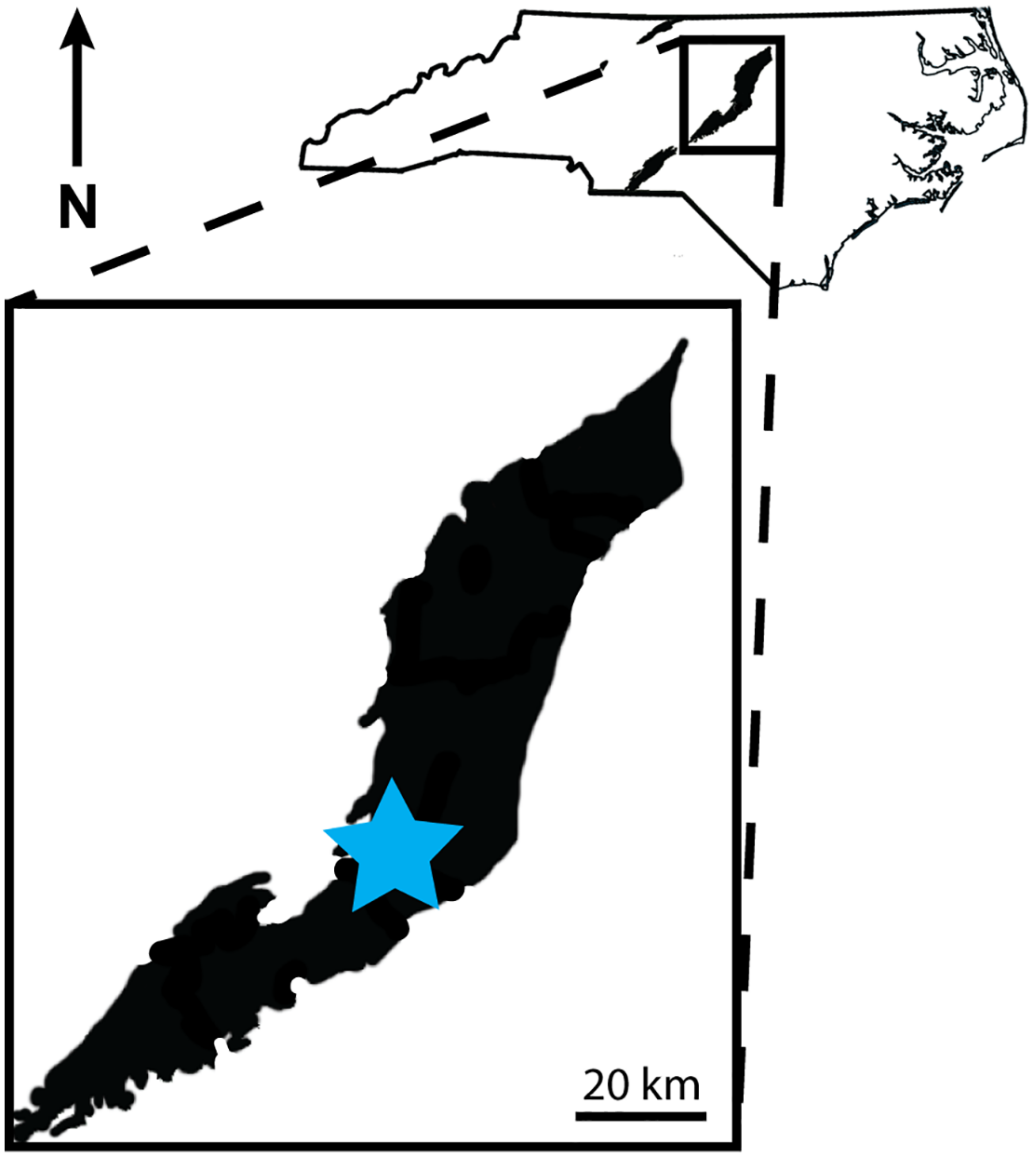
Type locality for *Carnufex carolinensis*. General location of the type locality for NCSM 21558, NCSM locality NCPALEO1902 (blue star) within the Triassic basins of the Newark Supergroup (black) in the state of North Carolina.

*Carnufex carolinensis* was recovered from NCSM locality NCPALEO1902 in southeastern Chatham County, North Carolina (Fig 1). Exposed strata consist of “red-bed” siliciclastics that strike north-northeast and dip 25° southeast. The sediments at this site represent a fluvial environment with lithologic units cycling between rusty-red and purple siltstones (floodplain) and light gray sandstones and conglomerates (river channel) every 5 to 10 meters. NCSM 21558 was collected from a red conglomerate with the majority of clasts <1cm in size, suggesting that the animal was deposited in the river channel or crevasse splay adjacent to the channel.

Strata at this site belong to the Upper Triassic Pekin Formation, the statigraphically lowest unit within the Chatham Group of the Deep River Basin [19–21]. The Deep River Basin is one of many Triassic basins exposed along the east coast that formed during the initial rifting that produced the Atlantic Ocean. The sediments in these rift basins are collectively referred to as the Newark Supergroup [22]. NCPALEO1902 is located within the Colon cross structure, a constriction separating the two sub-basins—the Durham basin in the north and Sanford Basin in the south—of the Deep River basin [20,21].

Since the work of Ward et al. [23], the Chatham Group has generally been considered Upper Triassic in age. Biostratigraphic studies, especially palynomorph assemblages from within the Colon cross-structure, date the Pekin Formation to the early Carnian [24–27]. However, magnetostratigraphy and recent revisions to the Late Triassic timescale indicate a younger deposition in the late Carnian, roughly 231 Ma [28–31].

## Materials and Methods

### Specimens

**Fig 2:**
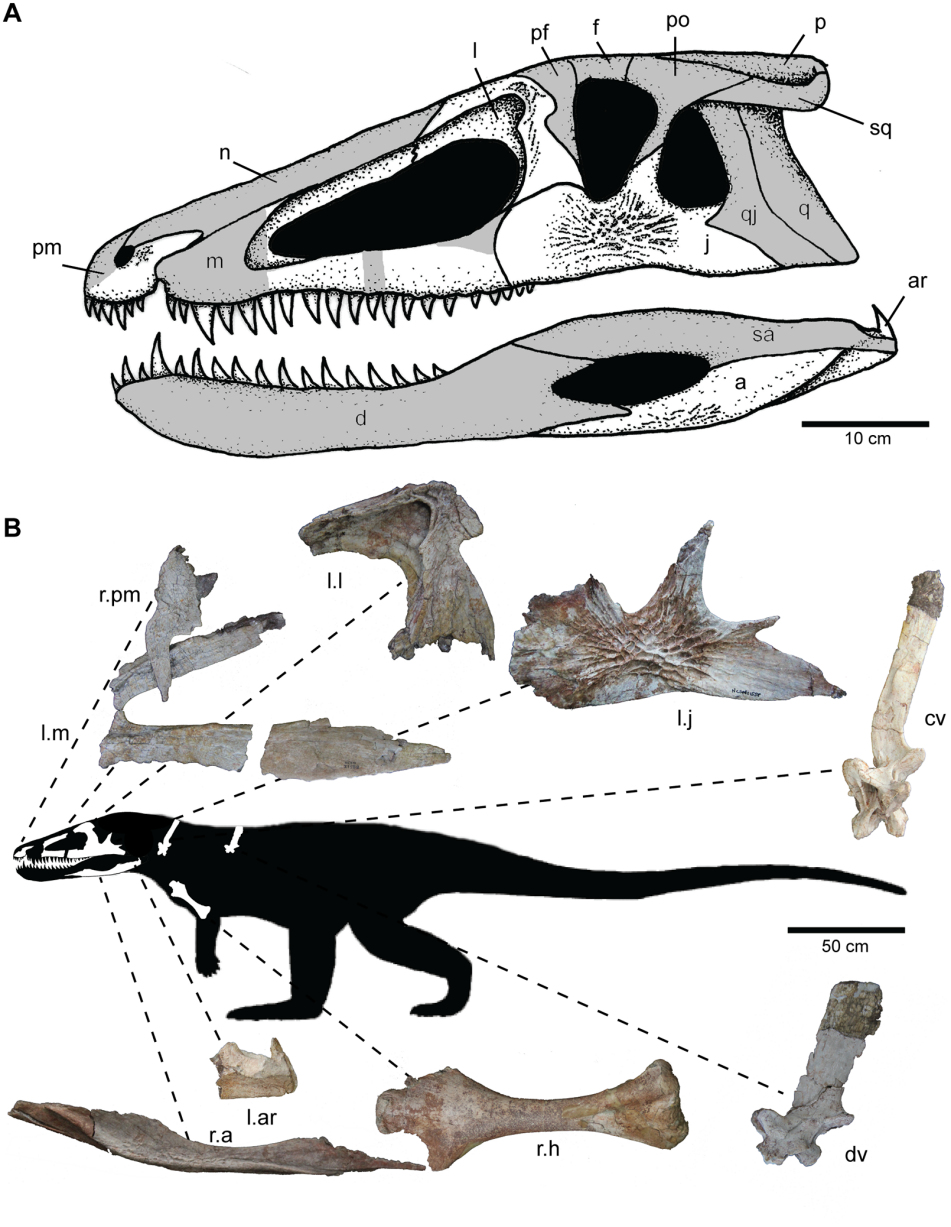
Skeletal reconstruction of *Carnufex carolinensis*. (A) Reconstruction of the skull of *Carnufex carolinensis* with preserved portions in white and inferred portions in gray. (B) Full body silhouette with preserved elements of NCSM 21558 highlighted. Abbreviations: a, angular; ar, articular; cv, cervical vertebra; d, dentary; dv, dorsal vertebra f, frontal; h, humerus; j, jugal; l, lacrimal; l., left; m, maxilla; n, nasal; p, parietal; pf, prefrontal; pm; premaxilla; po, postorbital; q, quadrate; qj, quadratojugal; r., right; sa, surangular; sq, squamosal.

NCSM 21558 was collected in 2003 from field locality NCPALEO 1902. Collection damage to the proximal head of the humerus and the atlas is evident. Remaining elements are complete to nearly complete and exhibit excellent preservation, including intricate ornamentation and thin (1 mm) laminae. All materials are disarticulated, three-dimensionally preserved, and exhibit almost no distortion (Fig 2). Although disarticulated, all elements collected from these blocks are considered to belong to the same individual. These elements were found in close association, are of a size consistent with a single animal (i.e., there are no distinctly large or small elements in comparison to the others), and there are no duplicate elements, suggesting that the specimen does indeed represent a single individual. The referred humerus is considerably smaller than the humerus of the holotype and was found at the same locality; it is referred based on the shared presence of an ectepicondylar crest just proximal to the radial condyle (an autapomophy listed for *Carnufex carolinensis*) and the similar overall shape of the distal head of the two humeri. The skull elements of the holotype are all exceptionally thin, most exhibit similar patterns of ornamentation, and adjacent elements—once prepared from the matrix—articulate well. In addition, there are no other clasts of a size similar to the bones (all pebbles are <1cm) and all elements are for the most part whole, suggesting that they were not transported far and that the deposit does not represent a mixed accumulation of bones.

Both the holotype (NCSM 21558) and referred specimen (NCSM 21623) are permanently housed in the vertebrate paleontology collections at the North Carolina Museum of Natural Sciences in Raleigh, North Carolina and are publically available for research. No permits were required for the described study, which complied with all relevant regulations. The specimens were collected from private land with permission from the landowner. Specific locality data is available on file with each specimen.

### Phylogenetic Analysis

We constructed a dataset expanding on Nesbitt [4] to determine the phylogenetic position of Car*nufex carolinensis* and to improve resolution among select paracrocodylomorphs, particularly throughout the transition from “rauisuchian”-grade loricatans to “sphenosuchian”-grade crocodylomorphs. Details on operational taxonomic units (OTUs), the list of characters, and discussion of character modifications are included in the Supporting Information (S1 and S2 Files).

The matrix (S3 and S4 Files) was constructed and edited in Mesquite version 2.75 [32] and consists of 214 binary characters and 34 multistate characters, twelve of which are ordered (characters 5, 12, 22, 39, 87, 99, 108, 186, 198, 202, 237, 250). The matrix of 41 taxa and 251 characters was analyzed using PAUP* version 4.0a134 [33]. PAUP* determined 10 characters to be parsimony uninformative (characters 10, 78, 167, 176, 181, 182, 205, 208, 219, 243). These characters were excluded a priori when calculating support values to prevent inflation of CI values [34]. Outgroup taxa (*Euparkeria capensis*, *Machaeroprosopus pristinus*, *Turfanosauchus dabanensis*, *Gracilisuchus stipanicicorum*, *Riojasuchus tenulsceps*, *Revueltosaurus callenderi*, and *Stagonolepis robertsoni*) were defined as paraphyletic with respect to the ingroup.

Branches with a maximum length of zero were collapsed [35]. Multistate codings were interpreted as polymorphisms (PAUP* treats these characters as heterogeneous terminal groups). “Gap” states were treated as missing data. All characters were equally weighted. A heuristic search strategy was employed (10000 replicates of random taxon addition sequences, saving 10 trees per replicate) with TBR branch swapping.

### Systematic Paleontology

ARCHOSAURIA Cope, 1869 [36] sensu Gauthier and Padian, 1985 [37]

SUCHIA Krebs, 1974 [38]

PARACROCODYLOMORPHA Parrish, 1993 [39] sensu Weinbaum and Hungerbühler, 2007 [40]

LORICATA Merrem, 1820 [41] sensu Nesbitt, 2011 [4]

CROCODYLOMORPHA Walker, 1968 [42] sensu Nesbitt, 2011 [4]

*Carnufex carolinensis* Zanno, Drymala, Nesbitt, and Schneider 2015 [6]

#### Holotype

NCSM 21558, a partial disarticulated skeleton including several well-preserved cranial bones and elements of the postcranial skeleton. The skull includes a dentigerous right premaxilla, left maxilla, left lacrimal, left jugal, left articular, right angular, and an isolated tooth. Elements of the postcranial skeleton include the atlas intercentrum, a cervical neural arch, dorsal neural arch, cervical rib, dorsal rib, gastralium, and the left humerus.

#### Referred Specimens

NCSM 21623, the shaft and distal end of a right humerus from a smaller-bodied individual.

#### Type Locality

NCPALEO 1902 in southeastern Chatham County, North Carolina, USA. Specific locality data is available by request from the NCSM.

#### Horizon and Age

A dark red, silty pebble conglomerate of the Pekin Formation, Chatham Group, Deep River Basin, Newark Supergroup. Carnian, Late Triassic, approximately 231 Ma.

#### Diagnosis

Follows Zanno et al. [6]. A large bodied (~3m), gracile crocodylomorph distinguished from all other basal crocodylomorph taxa by the following features (autapomorphies denoted by an asterisk): elongate, hypertrophied, subtriangular antorbital fenestra (approx. 14 cm anteroposteriorly long by 6 cm dorsoventrally high at posterior extent); posterodorsally trending ridge on lateral surface of maxilla terminating at edge of antorbital fenestra*; posterior process of maxilla tapers anteriorly, with minimum dorsoventral height at anterior corner of antorbital fenestra*; ornamented dermal skull bones (premaxilla, lacrimal, jugal, angular); posterodorsally deep antorbital fossa on anterior portion of the lacrimal, with a flange projecting anteriorly from posterior margin of fossa*; posterodorsal extent of antorbital fossa directly dorsal to the posteroventral extent (vertically oriented posterior margin)*; bifurcated posterior process of jugal bearing a small posterodorsally directed flange*; small, sub-conical, medial process of articular; pronounced posterodorsally trending ridge on posterior aspect of lateral surface of angular; ectepicondylar crest just proximal to radial condyle of humerus*; and reduced forelimb (humerus less than half estimated length of skull).

### Descriptive Osteology

*Carnufex carolinensis* is represented by two specimens: a partial skeleton (NCSM 21558) and an isolated partial humerus (NCSM 21623). NCSM 21558 comprises a closely associated yet disarticulated partial skull and several elements of the postcranial skeleton. With the exception of the premaxilla, humerus, and elements of the axial skeleton, all preserved material derives from the left side of the body. All preserved neural arches are unfused, indicating that NCSM 21558 is a skeletally immature individual [16,43], a conclusion bolstered by paleohistology data [44]. The skull is a minimum of 50 cm in length (Fig 2A), whereas the humerus is only 21cm in length, suggesting a bauplan (large skull and relatively small forelimb) similar to Triassic poposauroids (e.g., *Poposaurus*), rauisuchids (e.g., *Postosuchus*), and basal loricatans (e.g., *Batrachotomus*) (Fig 2B).

#### Premaxilla

**Fig 3:**
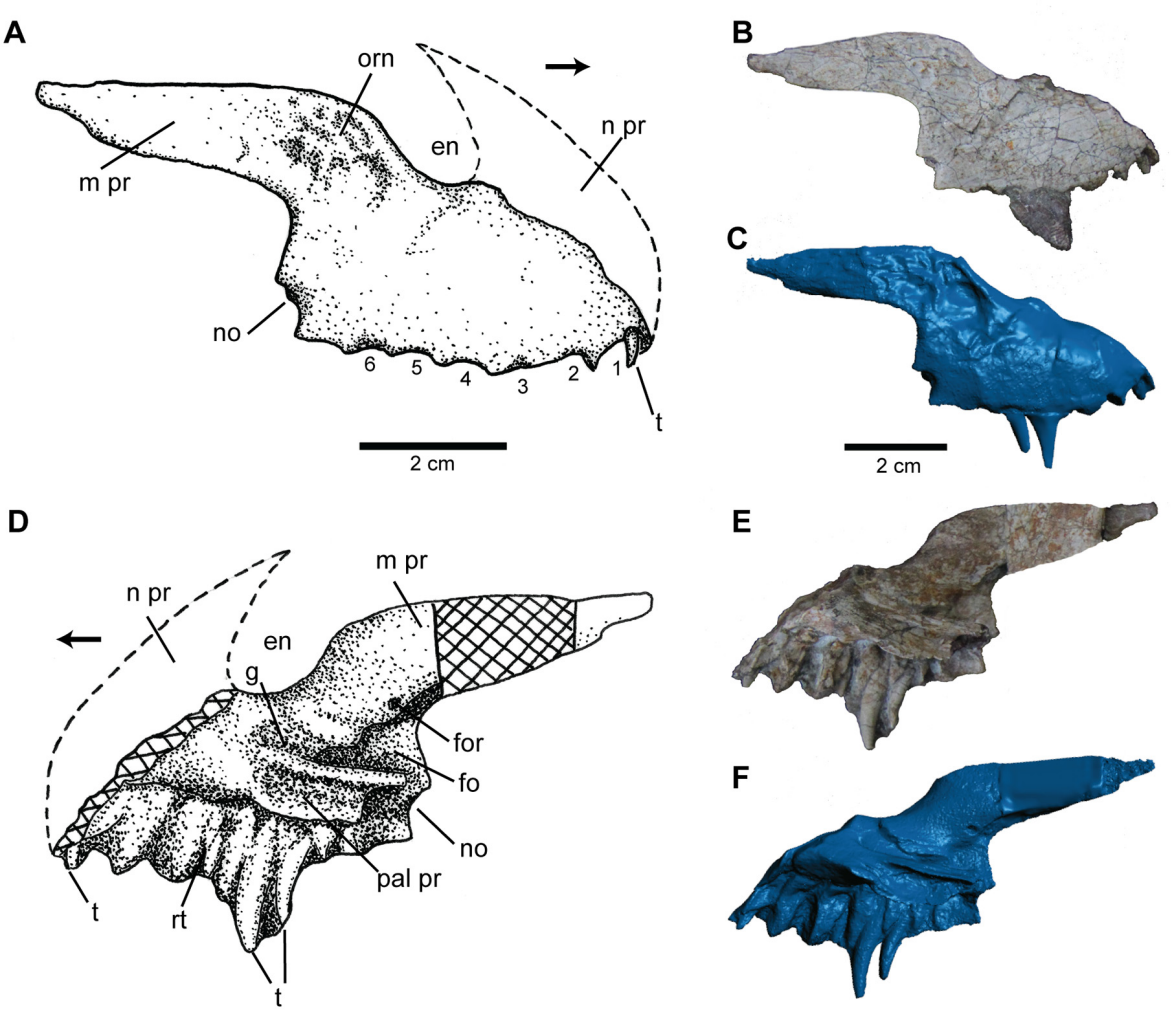
Premaxilla of *Carnufex carolinensis*. NCSM 21558 (holotype), right premaxilla in (A), (B), (C), lateral and (D), (E), (F), medial views. (A), (D), stipple drawing, (B), (E), photograph, and (C), (F), 3D surface scan rendering. Abbreviations: en, external naris; fo, fossa; for, foramen; g, groove; m pr, maxillary process; no, notch; n pr, narial process; orn, ornamentation; pal pr, palatal process; rt, replacement tooth; t, tooth. Alveoli numbered from anterior to posterior. Arrow denotes anterior direction.

The majority of the right premaxilla (Fig 3) is preserved, except for the anterior-most portion, which includes the anterodorsal process (nasal process = prenarial process). The main body is subrectangular and is at least twice as long anteroposteriorly as it is dorsoventrally tall, as preserved. A minimum of six alveoli (Fig 3A) are preserved in NCSM 21558, as may be the case in *Dromicosuchus* (NCSM 13733) and *Sphenosuchus* [45]. The fourth and fifth alveoli preserve their respective teeth and several replacement teeth are also present in various stages of eruption. The first four alveoli are roughly equal in size with the fifth and six being subsequently reduced relative to the other alveoli. The preserved portion of the premaxilla forms the ventral and posteroventral margins of the naris.

Posterior to the naris, the maxillary process (postnarial process) projects posteriorly, parallel to the alveolar margin, from the posterodorsal corner of the main body of the premaxilla. The maxillary process is mediolaterally thin and tapers posteriorly, such that the dorsal margin of the maxillary process and the ventral margin of the premaxillary body form an acute angle. The anterior portion of the maxillary process possesses fine, irregular ornamentation, which contributes to a slender ridge along the posteroventral margin of the naris. The posterior border of the premaxilla is smooth and produces a gentle curve as it meets the maxillary process. Also included in the posterior edge of the premaxilla is a small notch at the posterior-most portion of the alveolar margin. This suggests a restricted or loose articulation with the maxilla, and possibly a small subnarial foramen between the two elements, similar to that seen in *Batrachotomus* [46], *Dromicosuchus* (NCSM 13733), and “*Hesperosuchus”* (CM 29894). However, in the absence of the anterior-most portion of the maxilla, this configuration cannot be confirmed.

On the medial surface of the premaxilla (Fig 3D), the palatal process extends the full anteroposterior length of the main body. Posterior to the naris and the 4^th^ alveolus, the palatal process is separated from the main body of the premaxilla by a sulcus. The medial face of the main body of the premaxilla in this region also possesses a posteromedial-facing fossa (Fig 3D). These features are interpreted as part of the articulation with the maxilla. This portion of the palatal process is exceptionally thin (~1 mm) and forms a U-shaped concavity that opens medially with a slight dorsal overhang. A small groove is present ventral to the naris and stretches posteriorly to the area dorsal to where the palatal process becomes entirely separated from the main body of the premaxilla. An identical groove is seen in *Batrachotomus* [46]; however the extent to which the process is entirely separated from the main body of the premaxilla of *Batrachotomus* in the region of this contact is unclear.

The posteromedial projection of the palatal process and its subsequent detachment from the main body of the premaxilla in NCSM 21558 may have formed a small fenestra in the palate. Such a fenestra, formed anteriorly by the premaxilla and posteriorly by the maxilla, has been called an anterior palatal foramen [47] or a fenestra for the fourth dentary tooth [48] in *Terrestrisuchus* and *Dibothrosuchus* respectively. Such a fenestra may also be present in *Redondavenator* [3] and *Sphenosuchus* [45]. This configuration cannot be evaluated in articulated specimens (e.g., *Dromicosuchus*, “*Hesperosuchus*”, and *Junggarsuchus*), which now account for much of the diversity of basal crocodylomorphs, nor does it appear to be present in rauisuchids like *Postosuchus kirkpatricki* ([49]; UCMP 27572) and *Polonosuchus* [50]. In basal crocodylomorphs, this palatal fenestra appears to correlate with either a subnarial fenestra (e.g., *Dibothrosuchus*) or a lack of a distinct diastema between the maxillary and premaxillary teeth (e.g., *Terrestrisuchus*), yet not with a fully open notch between the maxilla and premaxilla (e.g., *Orthosuchus*), each condition appearing to accommodate a large fourth dentary tooth.

#### Maxilla

**Fig 4:**
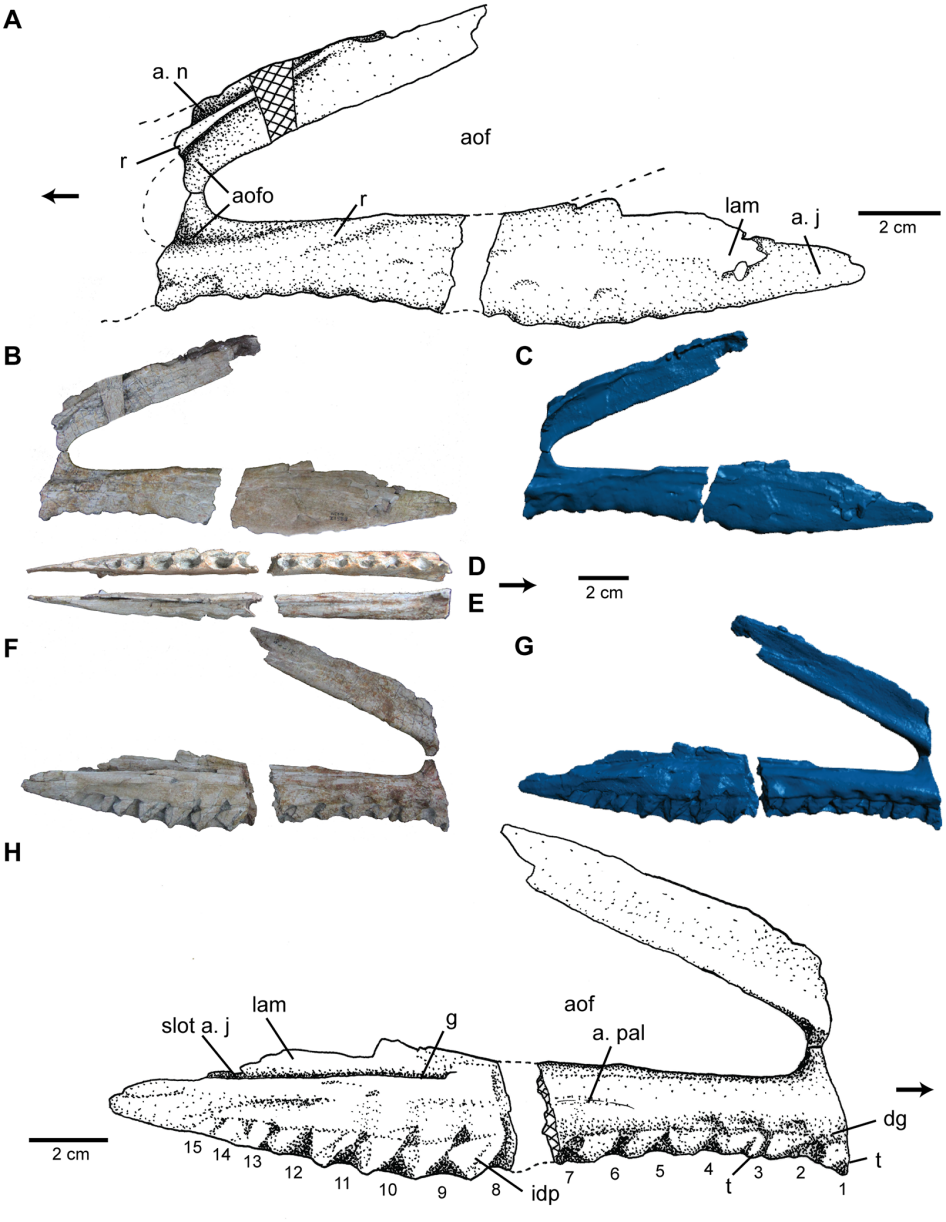
Maxilla of *Carnufex carolinensis*. NCSM 21558 (holotype), left maxilla in (A), (B), (C), lateral, (D), ventral, (E), dorsal (posterior process), and (F), (G), (H), medial views. (A), (H), stipple drawing, (B), (D), (E), (F), photograph, and (C), (G), 3D surface scan rendering. Abbreviations: a., articulation with; aof, antorbital fenestra; aofo, antorbital fossa; dg, dental groove; g, groove; idp, interdental plates; j, jugal; lam, lamina; n, nasal; pal, palatine; r, ridge; t, tooth. Alveoli numbered from anterior to posterior. Arrow denotes anterior direction.

The posterior portion of the left maxilla (Fig 4) is preserved, including a small area of bone anterior to the antorbital fenestra. The ascending process is complete, while the posterior process is broken into two pieces. A missing section, likely only 1–2 alveoli in length, separates the two preserved pieces of the posterior process. The facial portion of the maxilla anterior to the antorbital fenestra is not preserved. The maxilla possesses a minimum of 15 alveoli (Fig 4H). The maxilla forms the ventral and anterodorsal margin of a large triangular antorbital fenestra.

The ascending process of the maxilla is an elongate, spatulate process which projects posterodorsally at approximately 45° to the long axis of the element, similar to the condition in *Batrachotomus* [46] and *Postosuchus kirkpatricki* [49]. The ascending process of the maxillae of *Teratosaurus* and *Polonosuchus* have both been reported as projecting at 35° [51] and a number of poposauroids (*Xilousuchus*, *Arizonasaurus*, *Effigia*, and *Qianosuchus*) are reported at 50° [52]. The ascending process is mediolaterally thin (<3 mm) and much of it is included in the antorbital fossa. A sharp lateral ridge (Fig 4A) on the anterodorsal margin of the ascending process forms the anterodorsal portion of the margin of the antorbital fossa and the articulation with the nasal. The ascending process maintains the same width for nearly its entire length, except for a slight tapering where it contacts the lacrimal and/or nasal. The constant width and elongate nature of the ascending process is unique among Triassic and Early Jurassic paracrocodylomorphs. The ascending process in NCSM 21558 spans nearly 2/3 the length of the antorbital fenestra, whereas other basal crocodylomorphs such as “*Hesperosuchus*” (CM 29894, YPM 41198) and *Dromicosuchus* (NCSM 13733) possess ascending processes less than half the length of the antorbital fenestra. The ascending processes of *Sphenosuchus* and *Dibothrosuchus* show almost no posterior projection. In rauisuchids (e.g., *Postosuchus*), the ascending process of the maxilla tends to expand posteriorly and is long only in comparison to the otherwise short features of the face. On the other hand, basal paracrocodylomorphs (e.g., *Batrachotomus* and *Arizonasaurus*) tend to possess short, tapering ascending processes.

The posterior process of the maxilla is more than twice the length of the ascending process. It is sub-circular in cross-section anteriorly, but becomes mediolaterally thin and expands dorsoventrally at its posterior extent where it articulates with the jugal and meets the lacrimal to form the posterior border of the antorbital fenestra. This articulation is accomplished through a complex prong-like configuration formed by a tapering ventromedially arm that articulates with the medial surface of the jugal and a mediolaterally thin, dorsoventrally tall lamina that articulates with the lateral side of the jugal. This lamina is not well preserved, but evidence of its posterodorsal extent is visible on the articular face of the jugal. Such an articular configuration is difficult to diagnose in other taxa, since the thin laminar portion of the posterior process of the maxilla is not easily preserved in disarticulated specimens and is not always visible in articulated specimens. However, this arrangement may be present in a number of basal crocodylomorphs including *Dromicosuchus* (NCSM 13733), “*Hesperosuchus*” (CM 29894, YPM 41189), *Junggarsuchus* (IVPP V14010), and several undescribed basal crocodylomorph taxa.

The antorbital fossa is present on the anterior extant of the posterior process and is defined by a lateral ridge trending posterodorsally to project slightly into the antorbital fenestra before terminating (Fig 4A), dorsal to the 5^th^ preserved alveolus (4^th^ alveolus on the posterior process). This condition appears to be unique among suchian archosaurs. In other taxa, the lateral exposure of the antorbital fossa either terminates anterodorsal to the posterior process of the maxilla (e.g., *Protosuchus*, *Sphenosuchus*, and *Dibothrosuchus*), continues for the full length of the posterior process (e.g., *Dromicosuchus* and “*Hesperosuchus*”), or is poorly defined on the lateroventral face of the maxilla (e.g., *Postosuchus* and *Polonosuchus*).

On the dorsomedial surface of the posterior process is a groove (Fig 4H), which deepens posteriorly, eventually forming a slot between the thin lamina of the lateral surface and the main body of the process, just dorsal to the alveoli, for articulation with the jugal. On the medial surface of the posterior process, a slight step along the dorsal extent of the alveoli forms the dental groove (Fig 4H). Interdental plates (Fig 4H) are present between all but the last two alveoli. These interdental plates are fused to the maxilla but separate from each other, like most other paracrocodylomorph taxa, except for members of Rauisuchidae including *Postosuchus* [4]. Midway along the medial surface of the posterior process is a shallow, striated depression interpreted as the articular surface for the palatine (Fig 4H).

Within basal paracrocodylomorphs, a posteriorly facing foramen—often referred to as an “infraorbital foramen” [53]—on the medial side of the posterior process, dorsal to the palatine contact and approximately three alveoli posterior to the anterior extent of the antorbital fenestra, has been reported for many taxa, including *Postosuchus kirkpatricki* [49] *Polonosuchus* [50,51], *Teratosaurus* [50,51], *Batrachotomus* [46], *Decuriasuchus* [54]; *Arganasuchus* [55], and *Arizonasaurus* [56]. Such a foramen is clearly absent in NCSM 21588, but the phylogenetic or functional implications for its absence are difficult to assess because few basal crocodylomorph maxillae are preserved with the medial aspect exposed.

#### Lacrimal

**Fig 5:**
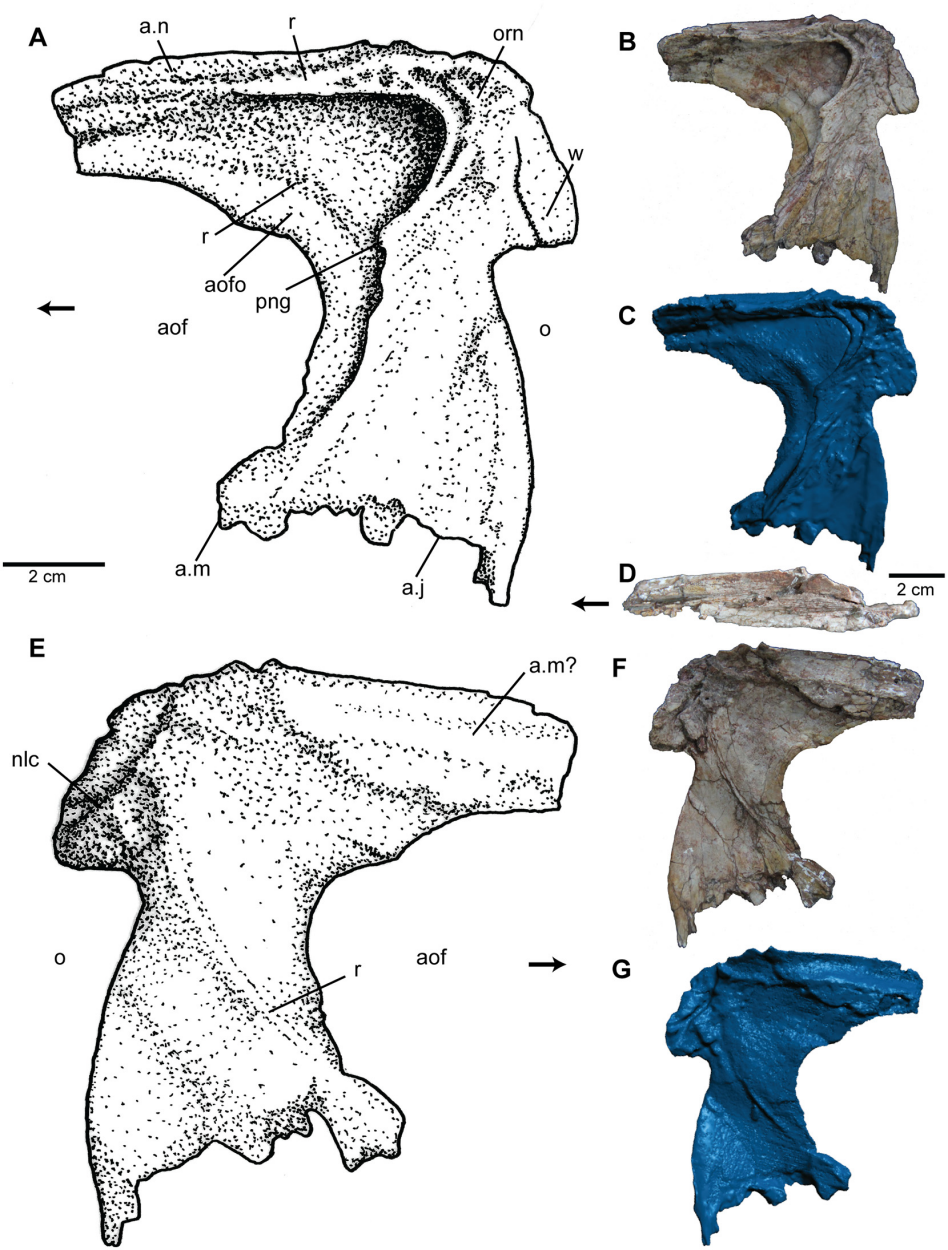
Lacrimal of *Carnufex carolinensis*. NCSM 21558 (holotype), left lacrimal in (A), (B), (C), lateral, (D), dorsal, and (E), (F), (G), medial views. (A), (E), stipple drawing, (B), (D), (F), photograph, and, (C), (G), 3D surface scan rendering. Abbreviations: a., articulation with; aof, antorbital fenestra; aofo, antorbital fossa; j, jugal; m, maxilla; n, nasal; nlc, nasolacrimal canal; o, orbit; orn, ornamentation; png, prong; r, ridge; w, wedge. Arrow denotes anterior direction.

The left lacrimal (Fig 5) is fairly complete, in which portions of the anterior process are poorly preserved and the distal end of the descending process is damaged. It is an exceptionally thin (2–4 mm in many areas), L-shaped bone. The lacrimal forms the posterodorsal margin of the antorbital fenestra and the entire anterior aspect and a small fragment of the dorsal margin of the orbit. The presence of a prefrontal is unclear in NCSM 21588 due to poor preservation on the medial side of the lacrimal; however, it is likely that a prefrontal contributed to the preorbital bar and played a role in the shape of the anterior margin of the orbit based on the configuration of these elements in related taxa (e.g. *Dromicosuchus grallator*, CM 29894). Attached to the posterodorsal margin of the left lacrimal is a small wedge of bone (Fig 5A), which may represent either a broken piece of the lacrimal or the left prefrontal. A groove and slight medial offset distinguishes this wedge from the main body of the lacrimal, but poor preservation makes it difficult to determine the presence of a suture. If this wedge of bone is the prefrontal, then it overlaps the lacrimal medially.

In dorsal view (Fig 5D), a laterally expanded ridge on the dorsal surface of the anterior process produces a narrow dorsal surface for articulation with the nasal and the posterodorsal margin of the antorbital fossa. The lateral surface of the lacrimal, excluding the antorbital fossa, is ornamented (Fig 5A). The dorsal portion of the lateral surface possesses a more rugose and irregular pattern of ornamentation with extensive pitting. Pitting becomes shallower and more linear on the descending process and is oriented posterodorsally.

The anterior and descending processes are approximately equal in size and length. Although most non-crocodyliform loricatans possess a lacrimal with an anterior process much longer than the descending process, *Sphenosuchus* [45] and *Batrachotomus* [46] bear a configuration more similar to NCSM 21558. The anterior process is wider posteriorly and tapers anteriorly. Approximately three centimeters of the anteriormost articulation with the maxilla is thought to be absent, whereas articulation with the nasal is slightly obscured. It appears as though the lacrimal overlaps the maxilla laterally and the nasal fits into a shallow dorsal groove on the anterior process of the lacrimal. The descending process is slightly constricted near the body of the lacrimal, whereas the ventral portion expands anteroposteriorly to overlap the jugal laterally and possibly contact a portion of the maxilla, as in other basal crocodylomorphs generally. This distal flaring of the descending process may also be present in *Dromicosuchus* (NCSM 13733). The posterior margin of the descending process in convex, but was likely modified by the prefrontal to produce a more straight or concave preorbital bar.

The posterodorsal aspect of the antorbital fossa is deeply recessed (Fig 5A) and forms a keyhole shape defined by a rugose ridge. The ridge juts anteriorly near the dorsal portion, producing a lateral prong as in some theropod dinosaurs, yet otherwise unknown in paracrocodylomorphs. The condition seen in NCSM 21558 is unique in that the prong does not extend past the fossa to laterally overlap the antorbital fenestra as it does in dinosaurs. In the region of the lacrimal, the antorbital fossa itself is distinct in that the posterodorsal corner lies directly above the posteroventral corner. In all other crocodylomorphs the posteroventral corner is posterior to the posterodorsal corner resulting in a posterior margin that is inclined forward, while the margin in NCSM 21558 is near vertical. Within the antorbital fossa, a gently raised ridge (Fig 5A) paralleling the concave anteroventral border of the lacrimal defines a shallow accessory fossa.

The medial surface of the lacrimal is relatively featureless except from some warping and fracturing. A canal (Fig 5E) in the posterodorsal region of the medial surface of the lacrimal emerges from the orbit and trends anteriorly and dorsally into a poorly preserved foramen near the dorsal margin of the lacrimal. This canal is interpreted as the nasolacrimal canal, with a similar structure to the canal reported for *Batrachotmous* [46]. A sharp, arc shaped ridge (Fig 5E) on the medial aspect corresponds to the ridge defining the antorbital fossa in lateral view. It is not clear if this ridge is a natural feature or taphonomic artifact. The dorsal-most portion of the medial surface is the most poorly preserved aspect of this element; however, a shallow fossa for the articulation of the maxilla appears to be present.

#### Jugal

**Fig 6:**
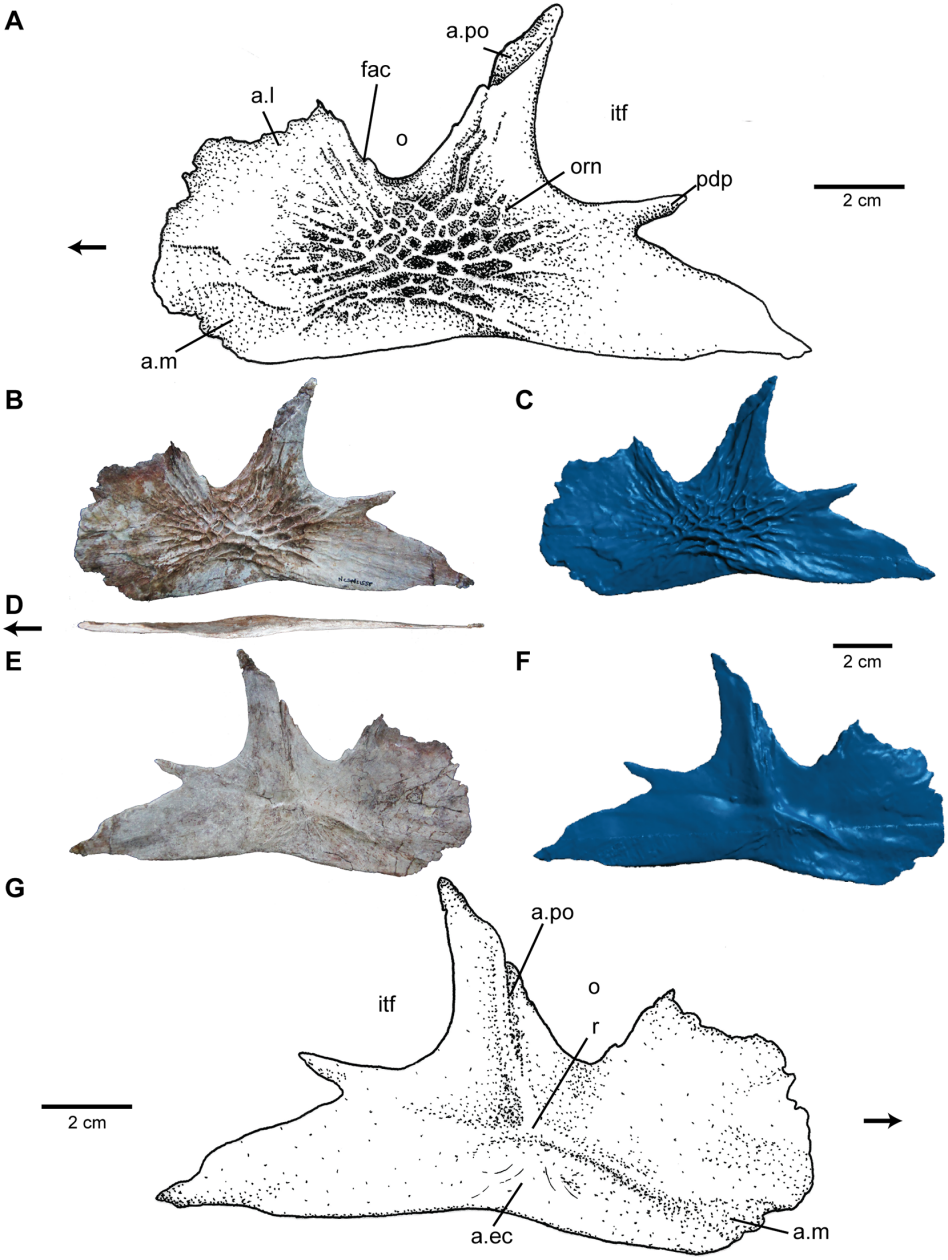
Jugal of *Carnufex carolinensis*. NCSM 21558 (holotype), left jugal in (A), (B), (C), lateral, (D), ventral, and (E), (F), (G), medial views. (A), (G), stipple drawing, (B), (D), (E), photograph, and (C), (F), 3D surface scan rendering. Abbreviations: a., articulation with; ec, ectopterygoid; fac, facet; itf, infratemporal fenestra; l, lacrimal; m, maxilla; o, orbit; orn, ornamentation; pdp, posterodorsal process; po, postorbital. Arrow denotes anterior direction.

The entire left jugal (Fig 6) of NCSM 21558 is preserved and is largely triradiate, although it is not readily comparable to the jugal of any other suchian archosaur, especially of the Triassic. It lacks any mediolateral thickening commonly seen in paracrocodylomorph jugals. The jugal forms the ventral margins of the orbit and infratemporal fenestra. The ventral margin of the jugal is smooth, with a slight dorsal convexity midway along its length, directly ventral to the postorbital process.

Anastomosing ornamentation covers the lateral surface of the jugal. The ornamentation is rugose and defined by random sub-circular pits in the center of the bone, becoming more radial and shallow towards the jugal margins. This pattern likely reflects the growth of the jugal, as elongation of ornamentation has been shown histologically to indicate direction of growth [57]. The distal ends of the anterior process and posterior process lack ornamentation where the element was covered by the quadratojugal and maxilla respectively.

The anterior process is dorsoventrally tall, flat and mediolaterally thin. The anterior margin of the jugal forms a half circle, from the ventral margin of the jugal to the orbit. Dorsal expansion of the jugal anterior to the orbit is common among non-crocodylomorph paracrocodylomorphs such as *Postosuchus* [49] and *Batrachotomus* [46], yet the semicircular anterior margin appears to be unique to NCSM 21558. The lateral surface of the jugal along the anterior margin bears a lightly striated, irregularly-shaped facet (Fig 6A) for the thin lateral lamina of the maxilla described above. This facet expands the full dorsoventral height of the anterior process. The lacrimal would have also articulated with the anterior process, merging with the maxilla, and laterally overlaping the anterodorsal margin. Much of the articular facet for the lacrimal is indistinguishable from the facet for the maxilla, except for a more defined sutural area within the margin of the orbit (Fig 6A), where a facet for a small arm of the descending process of the lacrimal is clearly visible.

The postorbital process of the jugal is triangular in shape, projects dorsally and slightly posteriorly from the main body, and tapers to a point dorsally, closely resembling the state in basal crocodylomorphs like *Dromicosuchus* (NCSM 13733) and “*Hesperosuchus*” (CM 29894). The articular margin for the postorbital on the postorbital process (Fig 6A) is recessed and faces posterodorsally, towards the orbit, wrapping onto the medial face of the jugal.

The posterior process of the jugal tapers to a point posteriorly and angles ventrally. Midway along the length of the dorsal edge of posterior process is a small (1.5 cm in length) process projecting posterodorsally. This posterodorsal process (Fig 6A) is seen in numerous dinosaurs [4], yet appears to be autapomorphic within Crocodylomorpha. In dinosaurs, a forked posterior process of the jugal bears two prongs of equal size, whereas the posterodorsal prong in NCSM 21558 is much smaller than the main ramus of the posterior process. The main arm of the posterior process of the jugal tapers to a point and angles ventrally, likely articulating ventral to the quadratojugal, as in crocodylomorphs and rauisuchids generally. It is not clear if the small posterodorsal process would have also articulated with the quadratojugal, or if it projected into the lower temporal fenestra.

The medial surface of the jugal is generally smooth and flat. A textured and deeply striated area (Fig 6G) extends from the anterodorsal edge of the postorbital process ventrally along its medial surface beside a ridge for articulation with the postorbital. A ridge on the medial side of the ascending process of the jugal is also present in *Batrachotomus* [46], although this is one of the only points of comparison to other taxa, as most basal paracrocodylomorph specimens either lack medial exposure of the jugal, or bear a completely different articular configuration for the ectopterygoid. The striated ridge of the postorbital process contributes to a slightly triradiate ridge (Fig 6G) on the medial surface of the jugal, which is interpreted as the articular junction between the jugal, postorbital, and ectopterygoid. One of the arms (anteroventral trending) of this three-pronged ridge forms the dorsal border of the maxillary facet (Fig 6G), whereas the other two (dorsal trending and posteroventral trending) form part of a subtriangular sutural region (Fig 6G) textured with elongate ridges for the ectopterygoid. Anterior to this, the anterior arm of the triradiate ridge fades out just dorsal to a lightly striated facet for articulation with the main body of the maxilla. The articulation for the quadratojugal is not clear in this specimen.

#### Angular

**Fig 7:**
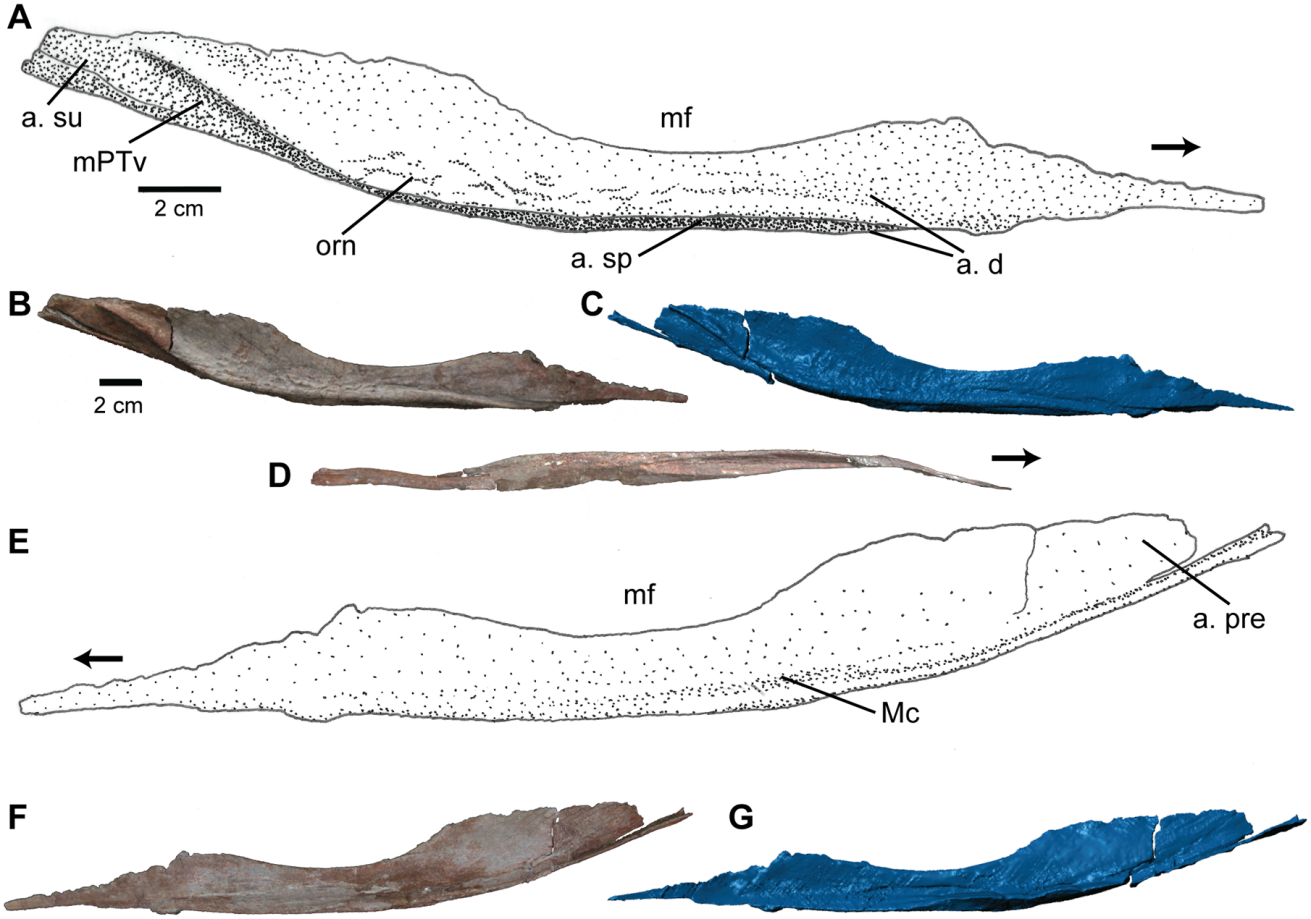
Angular of *Carnufex carolinensis*. NCSM 21558 (holotype), right angular in (A), (B), (C), lateral, (D), ventral, and (E), (F), (G), medial views. (A), (E), stipple drawing, (B), (D), (F), photograph, and (C), (G), 3D surface scan rendering. Abbreviations: a., articulation with; d, dentary; Mc, Meckelian canal; mf, mandibular fenestra; mPTv, insertion site of the m. pterygoideus ventralis; orn, ornamentation; pre, prearticular; sp, splenial; su; surangular. Arrow denotes anterior direction.

The complete right angular (Fig 7) of NCSM 21558 is well preserved. The angular is a long slender bone, which curves upward posteriorly in lateral view, forming most of the posteroventral margin of the mandible. Like many of the other bones of the skull, the angular is mediolaterally thin. A concavity in the dorsal edge of the angular forms the ventral margin of the elongate external mandibular fenestra (Fig 7). The lateral surface bears shallow anastomosing ornamentation, especially ventral to the mandibular fenestra. A prominent ridge (Fig 7A) extends from the anteroventral margin, beginning anterior to the mandibular fenestra, along the ventrolateral edge for nearly the entire length of the bone and then curves dorsally onto the lateral surface of the angular near its posterior termination. This ridge becomes more pronounced on the posterolateral surface, and likely represents the muscle-insertion site for the *M*. *pterygoideus ventralis* (Fig 7A). Amongst basal crocodylomorphs, a laterally exposed ridge on the posteroventral region of the angular is otherwise only seen in *Junggarsuchus* (IVPP V14010).

The ventral surface of the angular forms a rugose medial shelf that contributes to much of the posteroventral border of the mandible. On the medial surface of the angular, between the mandibular fenestra and the ventral shelf, a deep anteroposteriorly-trending groove forms the lateral portion of the Meckelian canal (Fig 7E), as in many other paracrocodylomorphs, including *Postosuchus* [49] and *Sphenosuchus* [45].

Anteriorly, beginning midway along the length of the mandibular fenestra, the angular is overlapped laterally by the dentary. The ventral margin of the angular in this region includes a deep groove (Fig 7A), likely for articulation with the splenial, and can be seen in lateral view, which is atypical for paracrocodylomorphs. However, this configuration may be influenced by slight distortion of the angular in this region. Posteriorly, the angular meets the surangular and prearticular. The posteriormost portion of the ventral face of the angular bears striations for articulation with the prearticular. The posterodorsal margin is not well preserved, but striations on the lateral surface suggest articulation with the surangular. The articular configuration for the angular is similar to that discussed by Walker [45] for *Sphenosuchus*.

#### Articular

**Fig 8:**
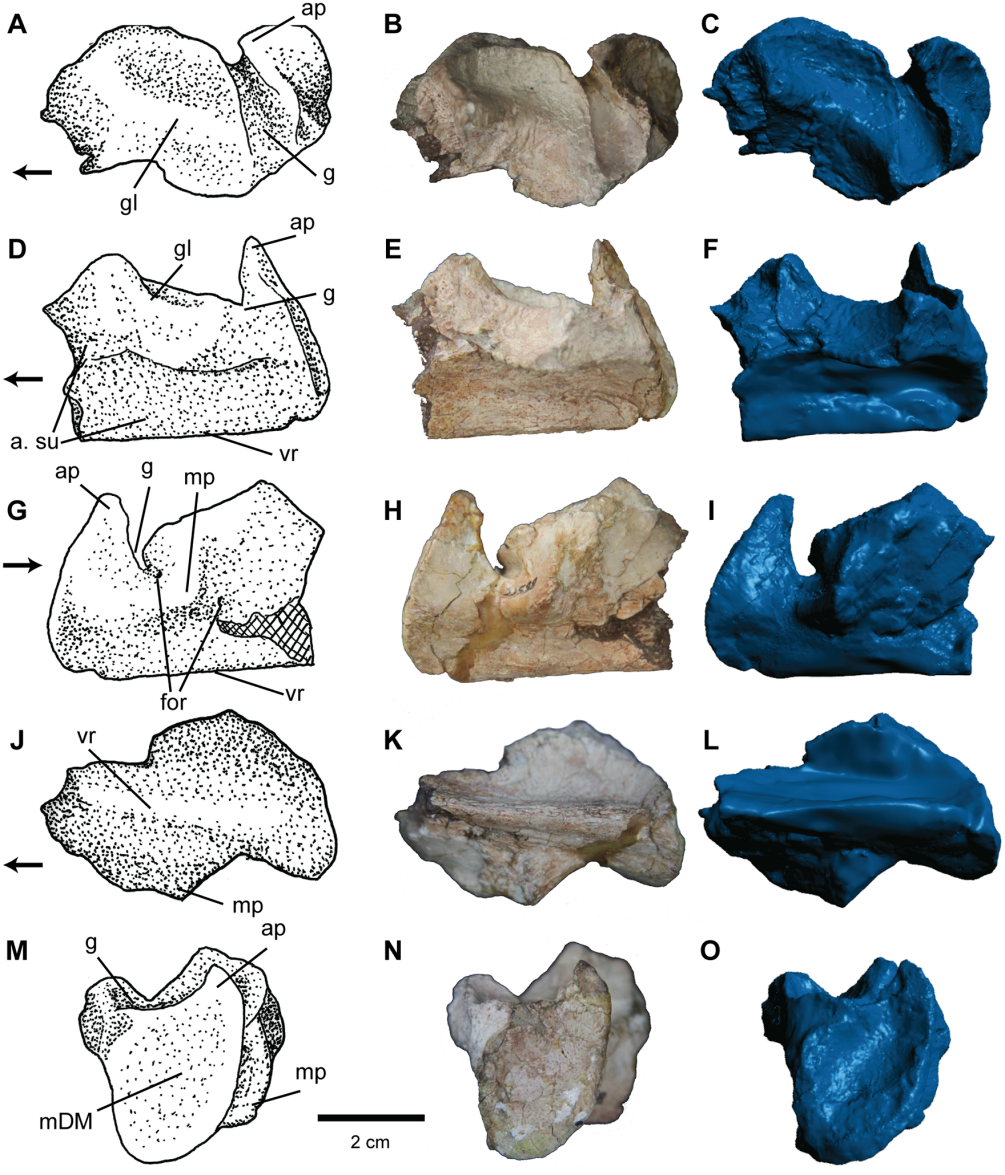
Articular of *Carnufex carolinensis*. NCSM 21558 (holotype), left articular in (A), (B), (C), dorsal, (D), (E), (F), lateral, (G), (H), (I), medial, (J), (K), (L), ventral, and (M), (N), (O), posterior views. (A), (D), (G), (J), (M), stipple drawing, (B), (E), (H), (K), (N), photograph, and (C), (F), (I), (L), (O), 3D surface scan rendering. Abbreviations: a., articulation with; ap, ascending process; for, foramen; g, groove; gl, glenoid; mDM, insertion site of the m. depressor mandibulae; mp, medial process; su, surangular; vr, ventral ridge. Arrow denotes anterior direction.

The articular is represented by the complete left element (Fig 8). The dorsal surface is dominated by a deep, saddle-shaped glenoid facet. The lateral edge of the glenoid and lateral surface of the articular ventral to the glenoid form the articular surface for the surangular. The lateral side of the articular forms a relatively flat rectangular facet and the posterolateral corner of the glenoid producing an overhang. Anterior to the glenoid is an elongate, textured concavity that marks the articulation with the surangular (Fig 8D). A deep transverse groove (Fig 8) separates the glenoid and posterior process of the articular. Laterally, the groove is more open, but becomes slightly constricted between the glenoid and the retroarticular process as it dives medially and curves anteriorly to meet a foramen (Fig 8G). Walker [45] considered such a foramen in crocodylomorphs to be the foramen aerum, but others [4,46] have argued that the term should be abandoned for use among paracrocodylomorphs because the foramen is not pneumatic and therefore not homologous. Chatterjee [58], along with subsequent authors (e.g., [46,49]), interpreted such a foramen as the passage of the chorda tympani branch of the facial nerve, and this interpretation is followed here. This nerve inserts just lateral to the medial process and exits the articular through a foramen (Fig 8G) anterior to the medial process. The medial process of the articular is small and sub-pyramidal in shape, unlike many other paracrocodylomorphs like *Postosuchus kirkpatricki* [49], *P*. *alisonae* (NCSM 13731), and *Batrachotomus* [46] that have a larger, tongue-like medial process. The ventral process forms a ridge running the full anteroposterior length of the articular and produces the ventral portion of the posterior process (retroarticular process). The posterior surface of the posterior process of the articular forms an ovate concavity (Fig 8M), similar to that seen in “*Hesperosuchus”* (CM 29894) and *Dromicosuchus* (NCSM 13733). This shallow concavity is interpreted as the attachment site for the *M*. *depressor mandibulae* [59]. The apex of the posterior surface forms a finger-like dorsomedial projection (Fig 8D), similar to the process seen in “*Hesperosuchus agilis”* (CM 29894), *Dromicosuchus* (NCSM 13733), *Sphenosuchus* [45] and many other basal crocodylomorphs. Although broken, this process appears to be much shorter in NCSM 21588 and *Sphenosuchus* [45] compared to *Hesperosuchus* (CM 29894) and *Dromicosuchus* (NCSM 13733), where it is nearly twice as long.

#### Dentition

**Fig 9:**
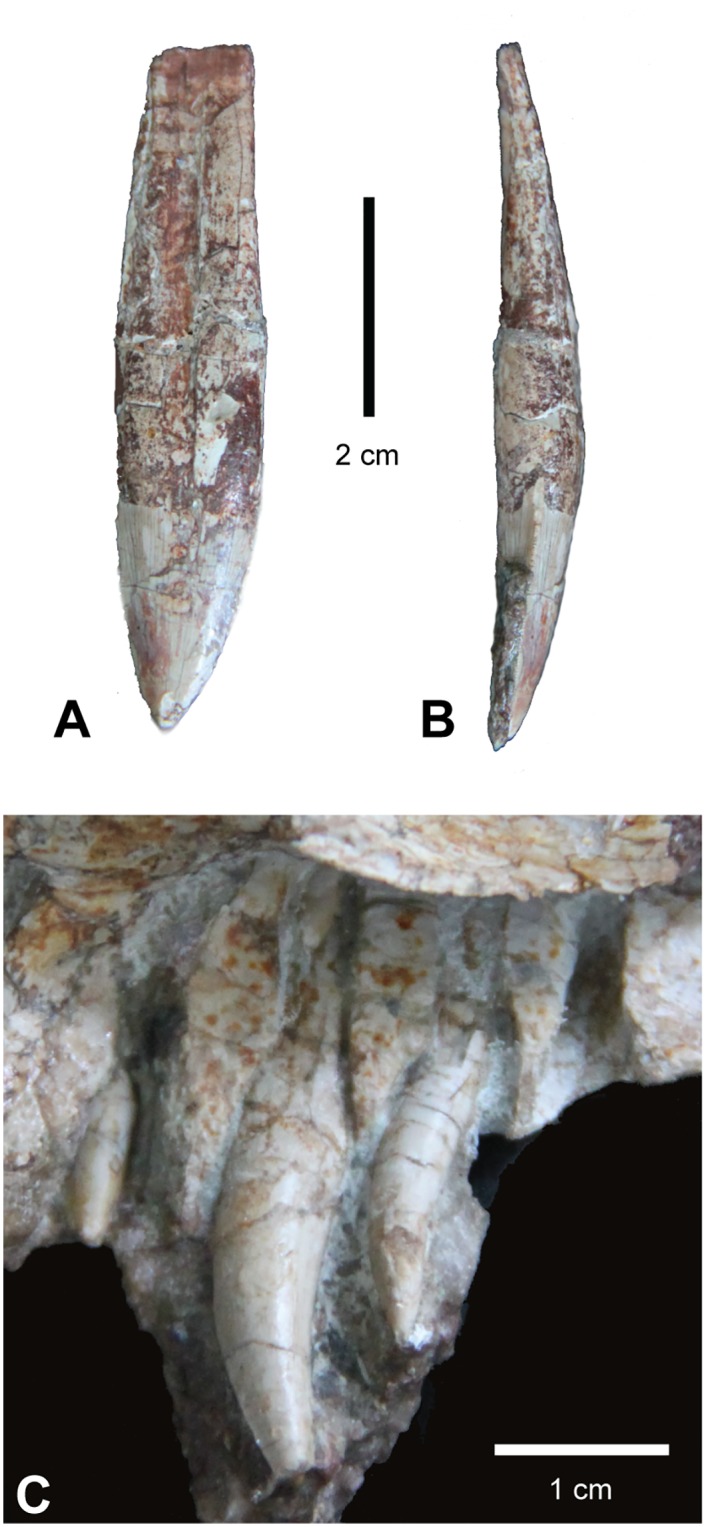
Dentition of *Carnufex carolinensis*. NCSM 21558 (holotype), dentition. Maxillary tooth in (A), lateral and (B), distal views. (C), In situ premaxillary teeth in lingual view.

Five premaxillary teeth are preserved in situ; however it is clear from the alveolar count that six premaxillary teeth were originally present. The two fully erupted premaxillary teeth are recurved and slender (Fig 9C), with the anterior of the two (presumably the 4^th^ premaxillary tooth) being slightly larger than the other (the 5^th^ premaxillary tooth). Serrations are present on both mesial and distal edges. Serration densities on the premaxillary teeth are approximately 8–9 per mm. Three addition replacement teeth are visible in the premaxilla (in alveoli 1, 2, and 4) in various stages of eruption.

Two in situ maxillary teeth and one isolated tooth (Fig 9A and 9B) are preserved for NCSM 21558. The isolated large tooth with its root was found in the same block as the jugal. All aspects of this tooth are consistent with the shape and size of the alveoli in the posterior portion of the posterior process of the maxilla. The tooth is convex on both mesial and distal carinae, which is consistent with being a posterior maxillary tooth of a crocodylomorph [4]. Taxa such as “*Hesperosuchus agilis*” (CM 29894) and *Dromicosuchus* (NCSM 13733) also possess posterior maxillary teeth with a convex distal edge, whereas non-crocodylomorph paracrocodylomorphs maintain straight or concave distal edges in all their maxillary teeth [4]. Although both carinae are convex, asymmetry in the degree of curvature results in a slightly recurved tooth. This, along with a bend in the root, suggests that the tooth came from the right side of the skull. The root of this tooth comprises approximately 70% of its total length. The tooth is mediolaterally compressed with serrations on both the mesial and distal edges. Serration densities on this isolated tooth are approximately 4–5 per mm.

#### Atlas

**Fig 10:**
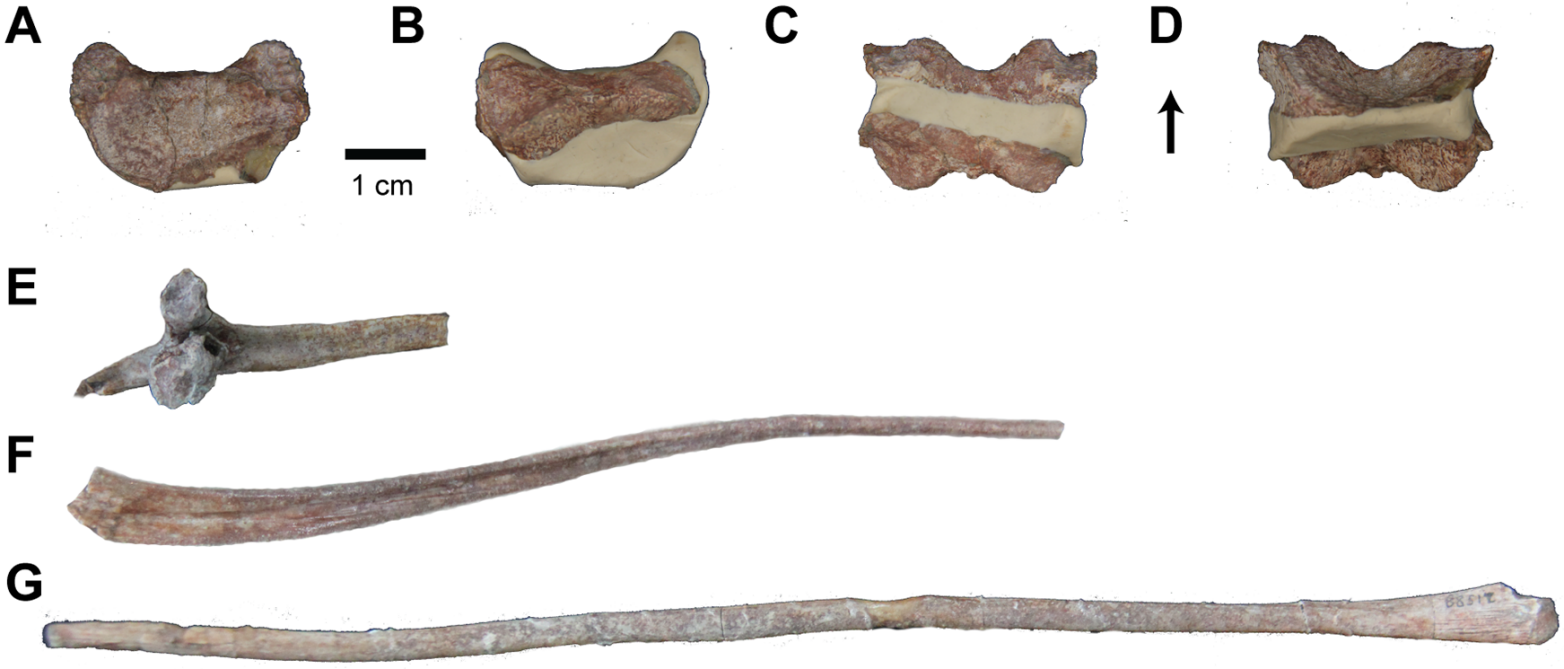
Atlas and Ribs of *Carnufex carolinensis*. NCSM 21558 (holotype). Atlas intercentrum in (A) anterior, (B) posterior, (C) dorsal, and (D) ventral views. (E), Cervical rib fragment; (F), Partial rib shaft; (G), gastralium. Arrow denotes anterior direction.

The intercentrum of the atlas (Fig 10) is preserved; although it was obliquely bisected by a rock saw during collection. The atlas has been described for three basal crocodylomorphs—*Hesperosuchus* [60], *Sphenosuchus* [45], and *Dibothrosuchus* [48]—yet is unknown amongst non-crocodylomorph paracrocodylomorphs except for a neural arch in *Effigia* [61] and an intercentrum in *Arizonasaurus* [56]. It should be noted that anatomical orientation as discussed here is based on limited comparative material. The intercentrum is crescent-shaped in anterior view, with the shallowly concave dorsal surface forming the floor of the neural canal. The anteroventral surface forms semicircular concavity for the dorsal portion of the occipital condyle. From this depression, if the occipital condyle occupied the entire facet, then its width is inferred to be 27mm. From the dorsal rim of the occipital facet, two short prongs project anteriorly, just lateral of the midline on either side. Dorsolateral to these prongs are triangular articular facets for the neural arch of the atlas.

#### Cervical Vertebrae

**Fig 11:**
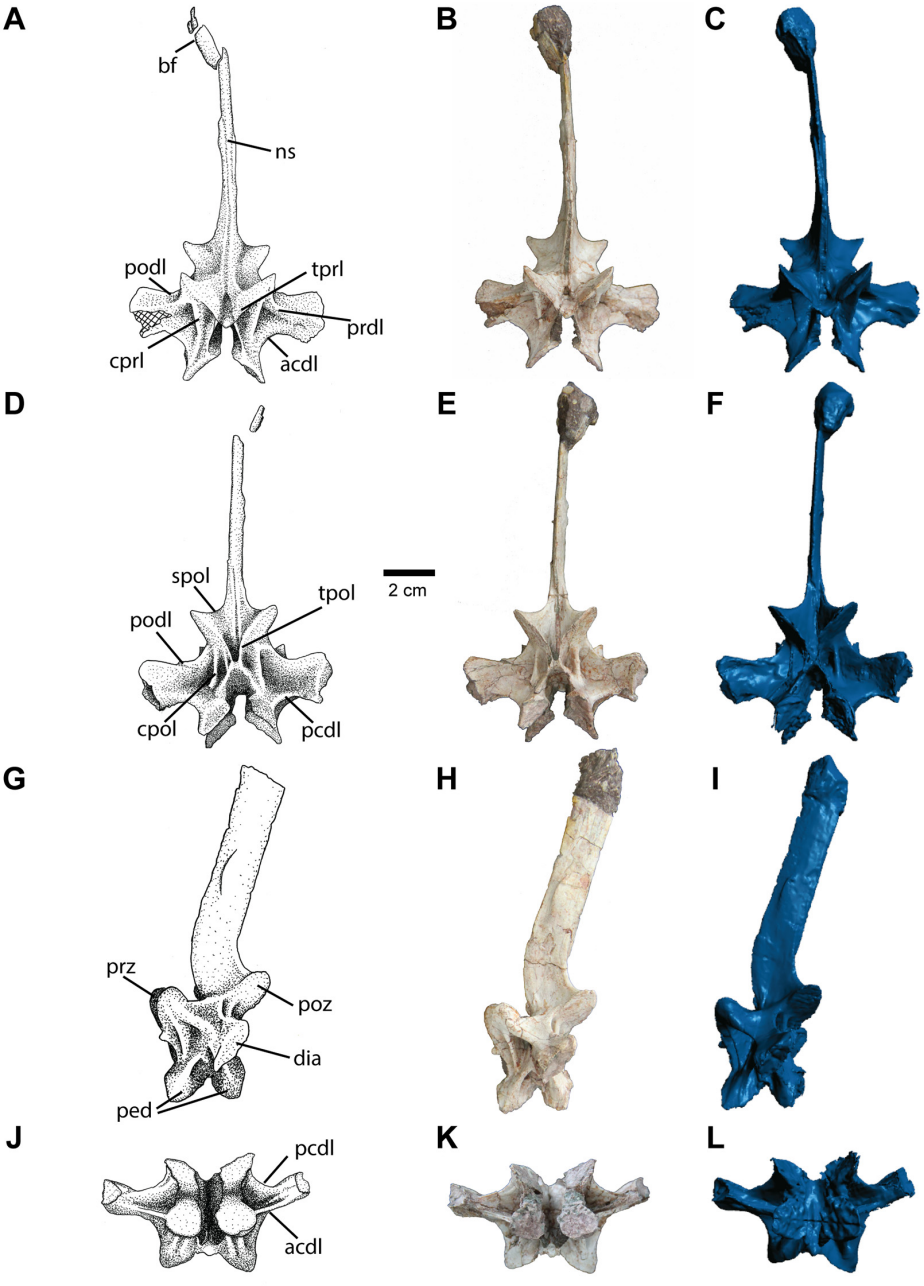
Cervical vertebra of *Carnufex carolinensis*. NCSM 21558 (holotype), cervical neural arch in (A), (B), (C), anterior, (D), (E), (F), posterior, (G), (H), (I), left lateral, and (J), (K), (L), ventral views. (A), (D), (G), (J), stipple drawing, (B), (E), (H), (K), photograph, and (D), (F), (I), (L), 3D surface scan rendering. Abbreviations: bf, bone fragments; dia, diapophysis; ns, neural spine; ped, pedicle; poz, postzygapophysis; prz, prezygapophysis. See text for lamina abbreviations.

A single posterior cervical neural arch (Fig 11), comprised of all but the distal tip of the spine, is the only element preserved of the cervical series. The neural arch does not appear to be broken, rather it is separated along the pedicles, suggesting that the neurocentral suture was completely open, supporting the interpretation of NCSM 21558 as a skeletally immature individual [44]. Paired pedicles form the ventral portion of the neural arch on each side of the vertebra. The pedicles are well separated, forming a deep W-shaped ventral margin in lateral view. The ventromedial facing articular surfaces of the pedicles are irregular, as expected for later fusion with the centrum.

The neural arch is slender with at least nine laminae (Fig 11) as defined by Wilson [62] present on each side of the neural arch along with a total of twelve fossae (*sensu* [63])—five on each side and two along the midline—which are described as follows. Laminae and corresponding fossae have been reported in a number of non-crocodylomorph paracrocodylomorphs, including, *Effigia* [61], *Arizonasaurus* [56], *Batrachotomus* [64], and *Postosuchus alisonae* [65], in varying configurations. The neural canal is roughly triangular in cross-section and comparable in size to the neural canals of other large-bodied paracrocodylomorphs (e.g., *Postosuchus kirkpatricki* and *Batrachotomus*). The neural spine maintains the same anteroposterior width for its entire height. The height of the neural spine is at least twice that of the neural arch, although the dorsalmost portion of the spine is missing, making the total height and the presence or absence of a distal expansion unclear. The neural spine rises anterodorsally from directly anterior to the postzygaphyses at first, then curves posterodorsally, forming an approximately 30° incline relative to vertical (Fig 11G).

The transverse processes (diapophyses) are relatively short and originate midway along the anteroposterior length of the neural arch. The diapophyses are dorsoventrally elongate in cross-section with a slight posterior incline ending in an ovate articular surface (Fig 11G). The diapophyses in NCSM 21558 are similar in shape to those of the dorsal vertebrae of *Postosuchus alisonae* (NCSM 13731). Unlike the diapophyses of cervical vertebrae of many paracrocodylomorphs such as *P*. *alisonae* (NCSM 13731), *Batrachotomus* [64], and *Dromicosuchus* (NCSM 13733), which project ventrolaterally, the diapophyses of the cervical vertebra of NCSM 21558 project only horizontally (at a right angle to the neural spine).

The pre- and postzygapophyseal facets are ovate (longer mediolaterally) and steeply inclined. The steep incline contributes to a shallow spinoprezygapophyseal fossa (sprf) along the midline at the confluence of the prezygapophyses at the base of the neural spine. A deeper spinopostzygapophyseal fossa (spof) is present in the equivalent location on the posterior aspect of the neural arch.

Accessory articulations appear to be present, although with differing morphology compared to the more typical hyposphene-hypantrum articulations seen in many non-crocodylomorph paracrocodylomorphs including *Arizonasaurus* [40,56], *Effigia* (AMNH FR 30587, [61]), *Batrachotomus* [64], *Postosuchus kirkpatricki* [5], and *Postosuchus alisonae* (NCSM 13731). In NCSM 21558, a small tubercle is present between the prezygapophyses, just above the neural canal. Posteriorly, a U-shaped gap is formed between the base of the postzygapophyses. This gap is created by the laminar posterior edges of the postzygapophyses bending ventrally to meet the roof of the neural canal, rather than continuing ventromedial to contact each other directly. This condition is distinct from the more typical hyposphene seen in taxa like *P*. *alisonae* (NCSM 13731), which appears as a vertical lamina projecting from the convergence of the postzygapophyses; the “hyposphene” in NCSM 21558 is instead separated into two laminae by a small gap.

An anterior centrodiapophyseal lamina (acdl) rises dorsolaterally from the anterior pedicle (neurocentral junction) to the ventral surface of the diapophysis. A posterior centrodiapophyseal lamina (pcdl) rises in the same manner from the posterior pedicle to meet the diapophysis at the equivalent posterior location as the acdl. These laminae define a deep centrodiapophyseal fossa (cdf) ventral to the diapophysis. A small wrinkle rising from the ventral aspect of this fossae along the pedicle may represent the incipient development of an additional accessory lamina, as it is present on both the right and left side of the vertebra. Such a case is present in *P*. *alisonae* (NCSM 13731), in which the sixth cervical vertebra possess three centrodiapophyseal laminae resulting in two fossae ventral to the diapophysis. The vertebrae of *Batrachotomus* [64] and *Arizonasaurus* [56] also possess deep fossae ventral to the diapophyses.

A prezygadiapophyseal lamina (prdl) connects the ventrolateral side of the prezygapophysis to the anterodorsal surface of the diaphysis. On the anterior side of the acdl, a centroprezygapophyseal lamina (cprl) rises dorsally from the anterior pedicle to meet the prdl on the ventrolateral surface of the prezygapophysis. The cprl projects dorsoventrally between two deep fossae ventral to the prezygapophysis—a prezygaphyseal centrodiapophyseal fossa (prcdf) laterally and centroprezygapophyseal fossa (cprf) medially. An intraprezygapophyseal lamina (tprl) defines the ventromedial margin of the prezygapophysis, beginning at the cprl, and the lateral margin of the neural canal. The tprl can be divided into two sublaminae—the prezygapophyseal portion and the portion bordering the neural canal—by the hypantrum, which also separates the tprl from its mirrored counterpart on the opposite side of the vertebra.

On the posterior aspect of the neural arch, a postzygadiapophyseal lamina (podl) defines the dorsal edge of the diapophysis from its lateral extent to the ventrolateral edge of the postzygapophysis. A short centropostzygapophyseal lamina (cpol) connects the ventrolateral portion of the postzygapophysis to the posterior pedicle and lies between two deep fossae ventral to the postzygapophysis—a postzygaophyseal centrodiapophyseal fossa (pocdf) laterally and centropostzygapophyseal fossa (cpof) medially. A small accessory fossa formed by a thin lamina sits on the medial wall of the left cpof, just lateral to the hyposphene. Similar to the configuration on the anterior portion of the neural arch, an intrapostzygapophyseal lamina (tpol) defines the ventromendial edge of the postzygapophysis and the lateral edge of the neural canal and is separated into two sublaminae by the hyposphene. Finally, a spinopostzygapophyseal lamina (spol) extends from the anteromedial face of the postzygapophysis to the base of the neural spine, just lateral of the midline.

#### Dorsal Vertebrae

**Fig 12:**
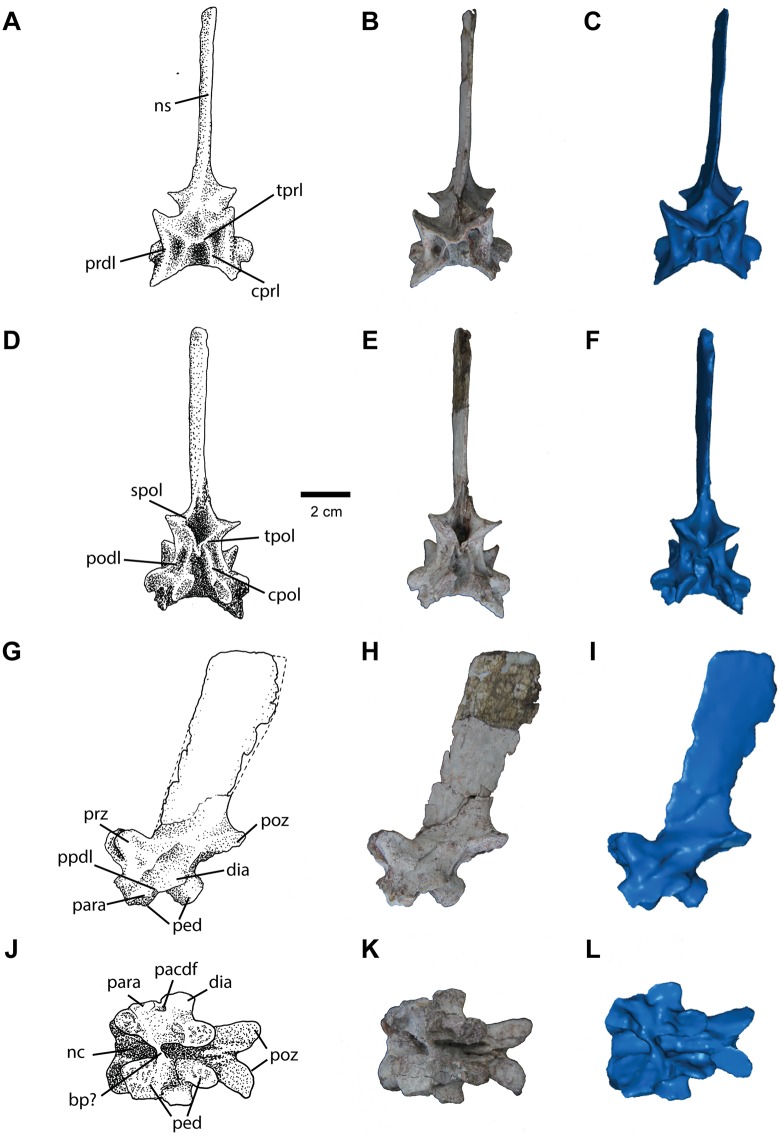
Dorsal vertebra of *Carnufex carolinensis*. NCSM 21558 (holotype), dorsal neural arch in (A), (B), (C), anterior, (D), (E), (F), posterior, (G), (H), (I), left lateral, and (J), (K), (L), ventral views. (A), (D), (G), (J), stipple drawing, (B), (E), (H), (K), photograph, and (D), (F), (I), (L), 3D surface scan rendering. Abbreviations: bp, bone pathology; dia, diapophysis; ns, neural spine; para, parapophysis; ped, pedicle; poz, postzygapophysis; prz, prezygapophysis. See text for lamina/fossa abbreviations.

A complete neural arch with spine (Fig 12) is the only element preserved of the dorsal series of the vertebral column. As with the cervical vertebra, the neural arch appears to have separated from the centrum along the neurocentral suture, preserving the delicate pedicles unbroken. The paired pedicles form the lower portion of the neural arch, with the lateral surface of the anterior pedicles forming the small, ovate parapophyses. The parapophyses are relatively low, suggesting that this was an anterior dorsal vertebra. The diapophyses are extremely short (<1cm) and situated midway between the two pedicles. The diapophyses are nearly flat dorsoventrally. They form a nearly continuous articular surface with the parapophyses except for a small notch between the two processes.

The neural arch is similar in size and shape to the cervical vertebra except that the postzygapophyses are considerably longer. The neural spine is slightly shorter and anteroposteriorly longer than that of the cervical vertebra, and is complete (Fig 12G). The spine is inclined posterodorsally at an angle similar to the cervical neural spine (approx. 20–30° from vertical). The neural spine is situated posteriorly on the neural arch, with the anterior extent at the base of the spine not extended anterior to the posterior extent of the prezygapophyses and the posterior portion of the base of the neural spine extending nearly to the posterior extent of the postzygapophyses. Dorsally, the neural spine expands and is longer anteroposteriorly than at its base, similar to the dorsal vertebrae known for many non-crocodylomorph loricatans (e.g., *P*. *kirkpatricki*, *P*. *alisonae*, *Batrachotomus*) and lacks a transverse distal expansion or “spine table”.

The pre- and postzygapophyses are arranged close to the midline, with ovate articular facets, and are more horizontally inclined compared to the cervical vertebra. The prezygapophyses are positioned directly dorsal to the anterior pedicles, whereas the entire articular face of the postzygapophyses extends posterior to the posterior pedicles. Medial laminae extend ventrally from the postzygapophyses to meet along the midline, projecting ventrally into the neural canal. A groove lies between them mirroring the condition in the cervical neural arch. However, this feature is restricted to the area between the postzygapophyses and does not project posteriorly; it is not clear if these laminae would have contacted the anterior face of the following vertebrae. Although clearly distinct, this condition may be homologous with the hyposphene of other non-crocodylomorph loricatans, or may reflect incipient loss of the hyposphene-hypantrum articulations as in other crocodylomorphs. A small, anteriorly directed tubercle is present between the prezygapophyses, also as in the cervical vertebra.

The dorsal vertebra displays fewer laminae (as defined by [62]) and fossae (as defined by [63]) compared to the cervical vertebra. Three fossae are preserved on each side of the arch as well as two along the midline. A broad, shallow spinoprezygapophyseal fossa (sprf) is present between the two prezygapophyses along the midline. Ventral to the prezygapophysis, a deep centroprezygapophyseal fossa (cprf) is formed by a thin centroprezygapophyseal lamina (cprl) medially and a thick cprl laterally. A deep spinopostzygapophyseal fossa (spof) is formed between the two postzygapophyses and the overhanging neural spine. This fossa is bordered by a spinopostzygapophyseal lamina (spol) on each side. On either side of the neural arch, ventral to the postzygapophyses, a shallow fossa is formed by two parallel centropostzygodiapophyseal laminae (cpol). A short paradiapophyseal lamina (ppdl) is present between the parapophysis and diapophysis, overhanging a shallow parapophyseal centrodiapophyseal fossa (pacdf).

Along the ventral side of the neural arch, ventral to the neural canal, a small region of bone is preserved stretching between the parapophyses (Fig 12J). The lack of broken surfaces suggests that the bone is not a piece of the centrum that failed to detach. The lack of symmetry in this feature suggests that it is not a normal part of the neural arch and may instead represent a pathology.

#### Ribs

The head of a cervical rib, a partial shaft of a rib, and a complete gastralium represent the remainder of the axial skeleton (Fig 10). All are especially slender elements. The gastralium is elongate and circular in cross-section. The lateral end is expanded with an ovate cross-section and striations for articulation. The shaft tapers towards the medial end, which is also striated. The partial rib is expanded and V-shaped in cross-section proximally and becomes more slender and circular distally. The proximal end, including the tuberculum and capitulum, is not preserved. The cervical rib is typical of paracrocodylomorphs, with two heads oriented at approximately 90° to each other, sub-circular articular facets, and an anterior flange-like projection. The distal end is not preserved.

#### Humerus

**Fig 13:**
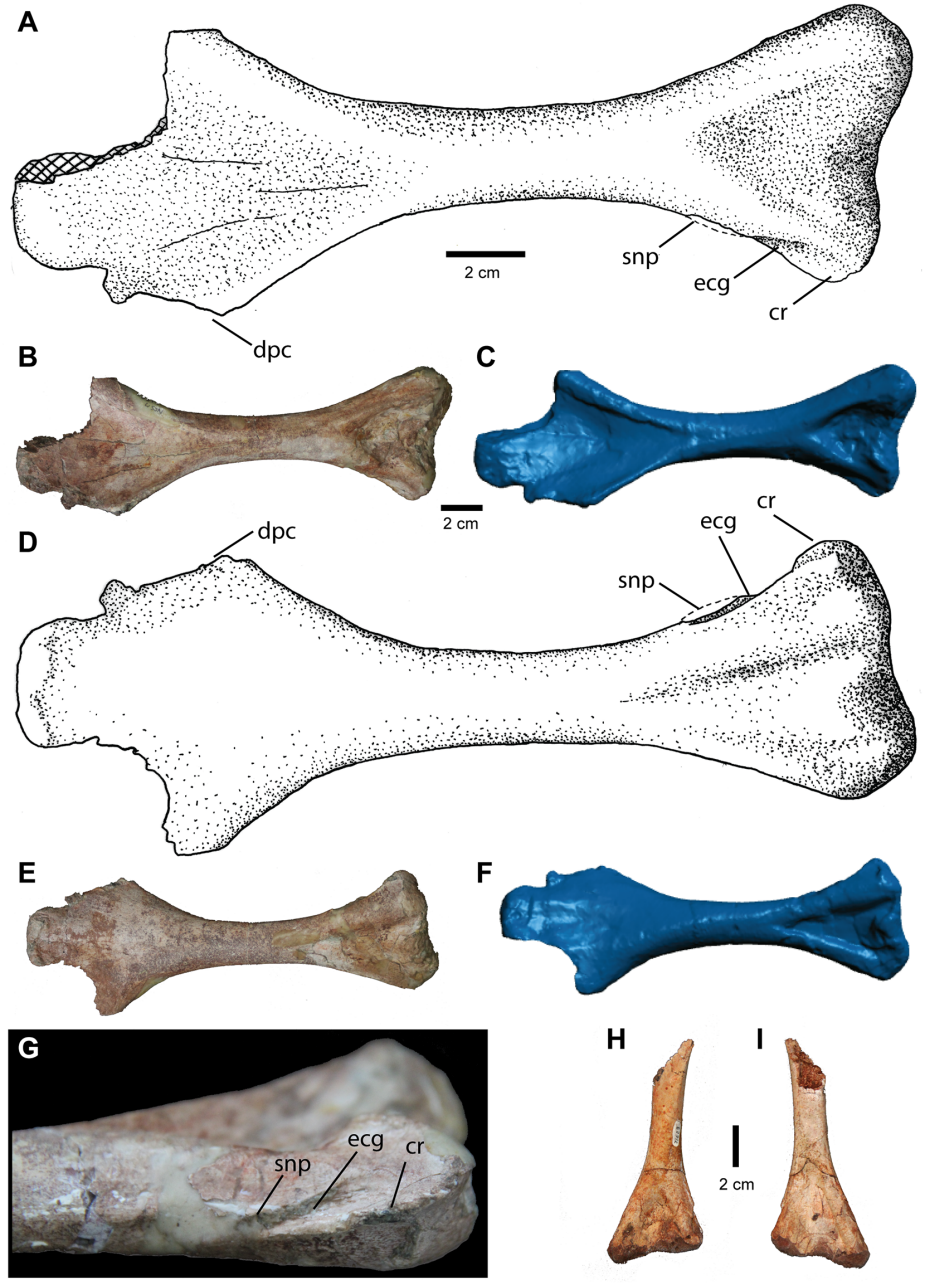
Humerus of *Carnufex carolinensis*. NCSM 21558, right humerus in (A), (B), (C), anterior view; (D), (E), (F), posterior view. (A), (D), stipple drawing, (B), (E), photograph, and (C), (F), 3D surface scan rendering. (G) Close-up photograph of ectepicondylar region in NCSM 21558. Left humerus (NCSM 21623, referred) in (H), anterior and (I), posterior view. Abbreviations: cr, crest; dpc, deltopectoral crest; ecg; ectepicondylar groove; snp, supinator process.

Two humeri (Fig 13) are known for this taxon—a nearly complete right humerus from the holotype specimen (NCSM 21558) and a referred partial left humerus (shaft and distal end) from a smaller individual (NCSM 21623). The distal end of the humerus is twisted laterally by approximately 30° relative to the proximal end, as is the humerus of *Postosuchus kirkpatricki* [5]. The proximal end of the humerus expands mediolaterally to at least 4 times the width of the shaft and thins anteroposteriorly to less than one centimeter. The posterior surface of the proximal region is slightly mediolaterally concave. Other distinct features of the proximal humerus, such as the deltopectoral crest, internal tuberosity, and humeral head are missing in this specimen, except for a slight expansion (Fig 13) on the proximal surface, which likely represents part of the humeral head. The shaft of the humerus is round in cross section, and is slightly wider mediolaterally than anteroposteriorly.

The distal end is expanded mediolaterally to approximately three times the width of the mid-shaft diameter. This proportion is typical for rauisuchids, such as *Postosuchus alisonae* (NCSM 13731) and *Postosuchus kirkpatricki* [5], yet differs from the distal humerus in basal crocodylmorphs such as *Dromicosuchus* (NCSM 13733) and *Hesperosuchus* (CM 29894), which exhibit a distal humerus less than twice the width of the shaft. The distal end is separated into two condyles, the radial condyle laterally, and the ulnar condyle medially. The two condyles are roughly equal in size and are separated by a wide, shallow trochlear groove. The ulnar condyle projects further ventrally than the radial condyle, offsetting the transverse axis of the distal condyles ventromedial to dorsolateral.

Dorsolateral to the radial condyle is a groove (Fig 13) for the passage of the radial nerve (ectepicondylar groove). This groove is bordered posteriorly by a small flange (supinator process), most of which is broken off in the holotype (Fig 13). The ectepicondylar groove and supinator process are present in several rauisuchids and basal loricatans, including *Postosuchus* ([5]; NCSM 13731) and *Batrachotomus* [63], but not in crocodylomorphs [4]. Also emanating from the lateral surface of the radial condyle is a thin crest (Fig 13) of bone, which begins at the distal-most end of the radial condyle and borders the ectepicondylar groove posteriorly. This crest tapers dorsally, terminating along with the ectepicondylar groove. This second crest associated with the ectepicondylar groove appears to be unique to this taxon and is present in both the holotype (NCSM 21558) and referred specimen (NCSM 21623).

## Basal Paracrocodylomorph Phylogenetics

### Analytical Results

Analysis of 251 characters and 41 taxa recovered 60 most parsimonious trees (MPTs), with a tree length (TL) of 654 steps. Subsequent *a priori* pruning of 10 parsimony uninformative characters (for which only one taxon was scored for the derived state) resulted in 60 MPTs, TL 644. A permutation tail probability test of 100 replicates was conducted, resulting in P = 0.01 and demonstrating that the signal within the data was more significant than a random dataset [66,67]. Parsimony scores for the MPTs include a consistency index of 0.450, retention index of 0.743, rescaled consistency index of 0.335, and homoplasy index of 0.564.

**Fig 14:**
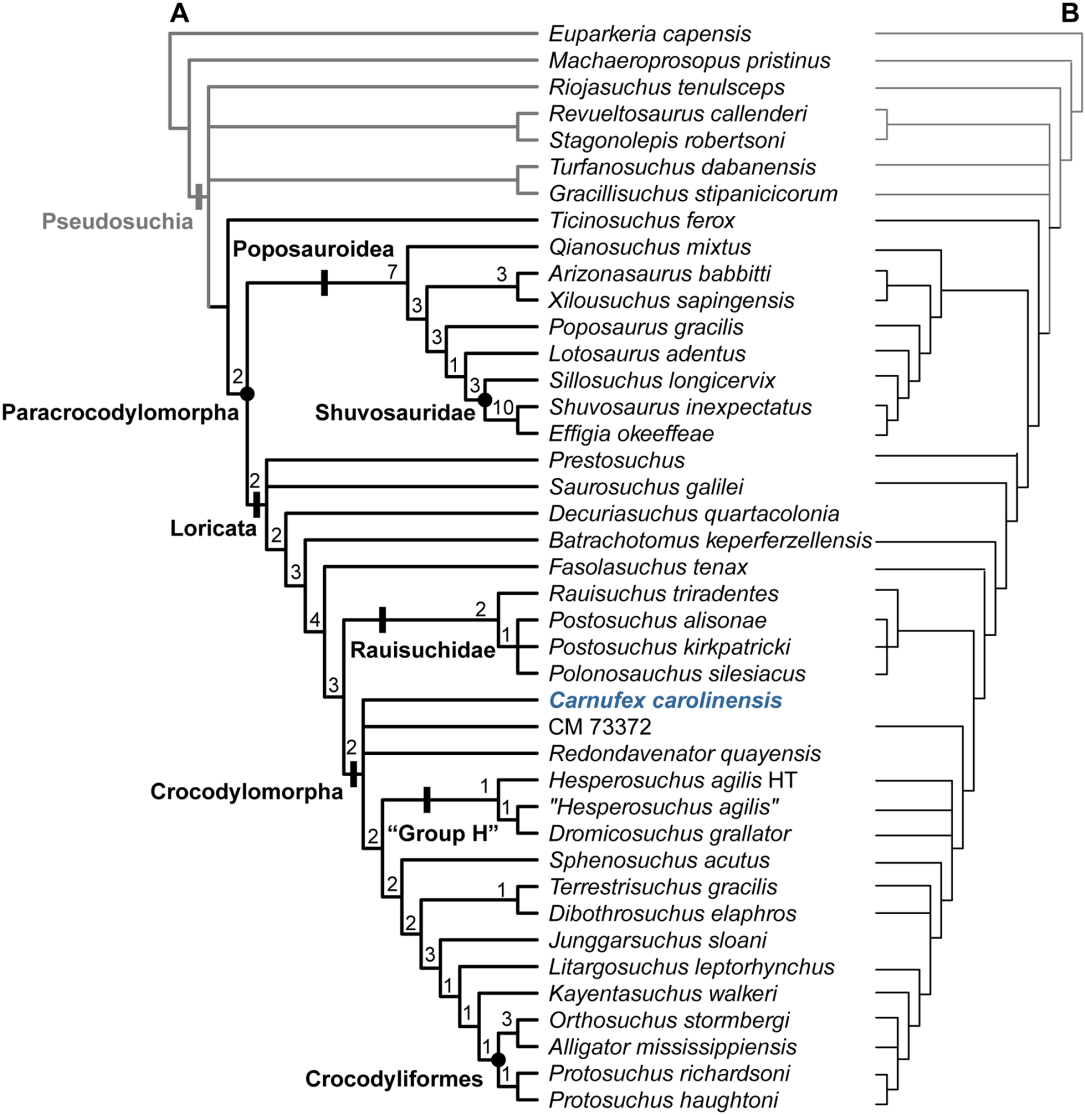
Phylogenetic relationships of *Carnufex carolinensis* within Paracrocodylomorpha. Strict consensus of 60 MPTs (TL 654, 41 taxa, 251 characters) with Bremer support values (A) compared with the corresponding portion of the tree produced by Nesbitt (2011) (B). Gray denotes outgroup assigned in present analysis. Circles = node-based phylogenetic definition; lines = stem-based phylogenetic definition. HT = holotype.

The strict consensus tree (Fig 14A) is well resolved, with only three polytomies within the ingroup. The three polytomies occur within Loricata, one including *Polonosuchus silesiacus* and the two species of *Postosuchus*; another at the base of Crocodylomorpha, including *Redondavenator quayensis*, CM 73372, and *Carnufex carolinensis* (large bodied basal crocodylomorphs); and the last one at the base of Loricata, including *Prestosuchus* and *Saurosuchus*. A semistrict consensus tree resulted in the same topology as the strict consensus. Intersection consensus methods resulted in increased resolution at the base of Crocodylomorpha. An Adams consensus retained the polytomy between *R*. *quayensis* and CM 73372, but *Carnufex* was found to be more closely related to all other crocodylomorphs than the other two basal-most members.

This analysis provides increased resolution within Crocodylomorpha when compared to previous studies (e.g., [4,8]). All polytomies within Crocodylomoroha recovered in previous analyses are resolved, and other species level relationships remain consistent, except for the basal loricatan polytomy (Fig 14). The present analysis recovers a novel clade comprising the holotype of *Hesperosuchus agilis*, specimens referred to *Hesperosuchus* (CM 29894 and YPM 41198), and *Dromicosuchus grallator*. Until more basal crocodylomorph taxa are included in a phylogenetic analysis and more robust support achieved for this clade, we regard it premature to diagnose and define the new referring to this novel grouping as “Group H.” In our analysis “*Hesperosuchus*” is posited as the sister taxon to *Dromicosuchus* and the two *Hesperosuchus* OTUs are recovered as paraphyletic with respect to *Dromicosuchus*. *Hesperosuchus* and *Dromicosuchus* are united by a well-defined antorbital fossa that completely surrounds the full circumference of the antorbital fenestra (char. 101:2) and the presence of a medial process on the proximal end of the radius (char. 176: 1). The holotype of *Hesperosuchus agilis* was recovered as the basal-most member of a clade containing *Dromicosuchus*, and all specimens currently referred to *Hesperosuchus*. This exclusive clade is supported by the presence of a distal expansion of the neural spine in the dorsal vertebrae (char. 132:0), a rod-shaped postglenoid process less than 50% of the total length of the coracoid (char. 162:2), and a convex ventral margin of the acetabulum (char. 189:0). This study fails to find support for a monophyletic clade of specimens previously referred to *Hesperosuchus*, underscoring the need to reevaluate CM 29894 and YPM 41198 with respect to the holotype (AMNH FR 6758) and other close relatives, such as *Dromicosuchus* (NCSM 13733).

Nested deeper within Crocodylomorpha, *Terrestrisuchus gracilis* and *Dibothrosuchus elaphros* form a clade, united by a posterior process of the maxilla that tapers posteriorly (char. 18:1), and a gently rounded anterior margin of the antorbital fenestra (char. 20:0). Among the representatives of Crocodyliformes included in the analysis, *Orthosuchus stormbergi* was found to be more closely related to *Alligator* than to *Protosuchus*. This suggests support for the monophyly of the genus *Protosuchus* and that *Orthosuchus* is not a member of “Protosuchia” (sensu [68]), although these relationships may be an artifact of limited taxonomic and character sampling in this apical portion of the tree. In addition, at the base of Loricata, *Prestosuchus* and *Saurosuchus galilei* form a polytomy. This differs from topologies in previous analyses (e.g., [4,6,8]) because *Prestosuchus* and *Saurosuchus* are sister-taxa in 50% of the MPTs.

### Morphological Trends in Early Crocodylomorph Evolution

Crocodylomorpha has long been recognized as monophyletic and is supported by numerous synapomorphies, including contact between the quadrate and prootic, a highly pneumatized braincase, a squamosal that overhangs the rear of the skull, and elongate radiale and ulnare [2,4]. The recent consensus on the paraphyletic nature of a non-crocodyliform Crocodylomorpha [4,14,17] has illuminated the stepwise acquisition of character states typifying crocodyliform taxa as they became increasingly specialized [2]. For instance, the work of Pol et al. [17] illustrates this pattern in aspects of the braincase in basal crocodylomorphs. The phylogenetic characters utilized herein highlight several additional key features commonly present in basal crocodylomorphs, especially those found in the lateral facial portions of the skull, the palate, the articular region of the mandible, and the forelimb and shoulder girdle.

Irmis et al. [2] suggested that basal crocodylomorphs represent a time of evolutionary experimentation with the gradual acquisition of character states typifying Crocodyliformes. In addition to the general trends identified here we present specific characteristics that can be identified as defining increasingly derived subclades. These data support an emerging picture of non-crocodyliform crocodylomorphs as part of a stepwise accumulation of key crocodyliform apomorphies. Nesbitt [4] thoroughly reviewed synapomorphies supporting several nodes within basal Crocodylomorpha. The characters identified by Nesbitt [4] are supplemented here with discussion of newly recovered nodes and the addition of newly identified features. Nesbitt [4] described morphological trends in relation to the following nodes: Crocodylomorpha; *Sphenosuchus* + Crocodyliformes; *Dibothrosuchus* + Crocodyliformes; *Litargosuchus* + Crocodyliformes; *Kayentasuchus* + Crocodyliformes; and Crocodyliformes. In the case of Crocodylomorpha, synapomorphies were discussed to the exclusion of CM 73372 and is therefore equivalent to “Group H” + Crocodyliformes. Herein, discussion of Crocodylomorpha follows the stem-based definition and therefore includes the three large-bodied taxa. In cases for which data is limited, characteristics are identified for the least inclusive clade for which the characteristic can be identified (delayed transformation).

Features present within Crocodylomorpha as recovered by Nesbitt [4] and this analysis include: a posterior process of the premaxilla that loosely overlaps the nasal/ anterodorsal margin of the maxilla laterally (char. 3:2); five or more premaxillary teeth (char. 5:3/4); a subnarial gap between the maxilla and premaxilla in lateral view (char. 7:1); an elongate anterior portion of the maxilla (char. 8:1); reduced size of the first two maxillary alveoli (char. 12:2); expanded palatal processes of the maxilla that meet at the midline but anterior and posterior expansion is restricted to along the midline (char. 22:2); small, subcircular external nares (char. 98:0); the region of the articular posterior to the glenoid is restricted, terminating just posterior to the glenoid with a flat or slightly concave posterior face (char. 109:1); elongate preacetabular process of the ilium extending anterior to the pubic peduncle, but shorter than the postacetabular process (char. 186:1); and a concave ventral margin of the acetabulam (char. 189:1).

Newly identified features present in “Group H” and more derived crocodylomorphs include: ventromedial process of the prefrontal present (char. 28:1); quadratojugal forms more than 80% of the posterior border of the lower temporal fenestra (char. 34:1); broad lateral expansion of the squamosal overhanging the lower temporal region (char. 44:1); quadratojugal-postorbital contact (char. 49:1); surangular foramen absent (char. 116:2); deltopectoral crest thin, projecting at about 90° to the long axis of the proximal head of the humerus (char. 171:1); width of the distal end of the humerus is less than 25% of the length of the humerus (char. 172:1); and metatarsal V tapers to a point and lacks phalanges (char. 250:2).

Newly identified features present in *Sphenosuchus* and more derived crocodylomorphs include: a convex posterior border of the postorbital process of the jugal (char. 56:1); and a postglenoid process of the coracoid greater than 50% of the total coracoid length (char. 162:3/4).

Newly identified features present in *Terrestrisuchus*, *Dibothrosuchus*, and more derived crocodylomorphs include: a broad dorsal exposure of the squamosal (char. 37:1); ulnare longer than the longest metacarpal (char. 180:1); roughly vertical orientation of the ilium (char. 187:0); pubic boot absent (char. 196:0); plate-like cross-section of the distal portion of the ischium (char. 203:0); articular surfaces of the ischium with ilium and pubis separated by non-articulating concave surface (char. 206:1); ischium about the same length or shorter than the dorsal margin of the iliac blade (char. 207:0); distal end of the fibula symmetrical (round or flat) in lateral view (char. 229:1); and four or fewer phalanges on pedal digit IV.

Features present in *Junggarsuchus* and more derived crocodylomorphs as recovered by Nesbitt (2011) and this analysis include: posterior portion of the nasals convex or flat at the midline (char. 23:0); posteroventral edge of parietals extends less than half the width of the occiput (char. 47:1); and a fenestrated body of the quadrate (char. 61:1).

Newly identified features present in *Litargosuchus* and more derived crocodylomorphs not discussed by Nesbitt [4] include: lateral margin of the paraoccipital process medial to the lateral extent of the upper temporal fenestra.

Newly identified features present in Crocodyliformes include: basipterygoid process of parabasisphenoid absent (char. 70:1); parabasisphenoid recess absent (char. 71:0); Eustachian tubes fully enclosed by bone (char. 87:2); supraoccipital excluded from the dorsal border of the foramen magnum (char. 91:1); diapophyses and parapophyses of middle dorsal vertebrae expanded on stalks (char. 134:1); flat presacral paramedian osteoderms (char. 147:0); appendicular osteoderms present (char. 148:1); anterior bar present on osteoderms (char. 150:1); abdominal osteoderms present (char. 151:1); blade-shaped postglenoid process of coracoid (char. 162:4); posterolaterally oriented glenoid (char. 165:0); pubis less than 70% of the length of the femur (char. 193:0); and obturator foramen of the pubis absent (char. 194:0).

Newly identified features present in members of “Group H” include a well-defined antorbital fossa that fully encompasses the antorbital fenestra (char. 99:2) and a medial expansion of the proximal head of the radius (char. 176:1).

## Evolutionary Implications and Paleoecology of Large-Bodied Crocodylomorphs

The large-bodied basal crocodylomorph, *Carnufex carolinensis* is unique among Triassic paracrocodylomorphs in possessing a large, slender, and ornamented skull and a mosaic of traits typifying other archosaur clades in addition to its numerous novel morphological traits. The latter include not only a large number of discrete autapomorphies, but also more subtle modifications of more widespread traits, such as the hyposphene-like laminations found in the cervical vertebra. As one of the earliest (Late Carnian) known loricatans—a clade occupying similar paleoecological roles as contemporary theropod dinosaurs [1]–and as a taxon representing a transitional position phylogenetically, it is not surprising that *Carnufex* exhibits features characteristic of other paracrocodylomorph subclades as well as theropod dinosaurs. However, the large number of uniquely derived characteristics within such a basal taxon is unexpected, raising additional questions about early crocodylomorph evolution and the morphological transition from early loricatans. More in-depth analyses of morphological change in this area of the archosaur phylogeny and renewed study on the impact of this variation in interpreting the various paleoecological roles of Late Triassic paracrocodylomorphs is needed.

Large-bodied crocodylomorphs encompass a range of variation that includes heavy ornamentation and highly autapomorphic skeletons, particularly in skull shape. This analysis represents the first study to find a distinct clade of crocodylomorphs with this bauplan. Recovery of these taxa as the earliest diverging members of Crocodylomorpha implies greater morphological diversity in the transitional grade between the small-bodied, gracile early crocodylomorphs, and their sister taxa, the robust, large-bodied rauisuchids at the base of Loricata than previously appreciated, moreover, its adds important new information to the evolution of body mass within the clade. The origin of Crocodylomorpha was hypothesized to occur along with a drop in body size [9]; however, *Carnufex*, *Redondavenator*, and CM 73372 were not included in the dataset that generated this result. If correctly posited by this study, these taxa suggest that morphological features typifying Crocodylomorpha appeared before a reduction in body size rather than subsequently.

Recent work by the authors [6] was the first to incorporate the large-bodied taxon *Redondavenator* into a phylogenetic analysis. This preliminary work recovered *Redondavenator* as the sister taxon to *Sphenosuchus*, as opposed to the position recovered here (in a polytomy at the base of Crocodylomorpha composed of the large-bodied taxa *Carnufex carolinensis*, *Redondavenator quayensis*, and CM 73372) based on a more targeted and expanded paracrocodylomorph dataset. However, a monophyletic Crocodylomorpha containing these taxa is only weakly supported (Bremer value 2) largely due to limited overlap in preserved elements between these taxa—premaxillae in *Carnufex* and *Redondavenator*, and a few vertebrae in CM 73372 and NCSM 21558. *Rendondavenator*, in particular is problematic. The extremely fragmentary nature of the single known specimen suggests the phylogenetic relationships of this enigmatic taxon will remain tentative until further skeletal material is recovered.

The lack of resolution in this region of the tree along with the broadly spanning temporal range of the currently known specimens of *Carnufex*, *Redondavenator*, and CM 73372 implies a large amount of missing data. *Redondavenator* and CM 73372 were recovered from late Norian/Rhaetian (approx. 208 Ma) strata of New Mexico [2], whereas *Carnufex carolinensis* comes from the Pekin Formation of North Carolina, which is now considered late Carnian or roughly 231 Ma in age [28–31]. Although missing skeletal material from known taxa is clearly an issue, these taxa are also separated in time by more than 20 million years, implying a large number of unsampled species may await discovery.

*Carnufex carolinensis* is the largest archosaur yet to be discovered in the Pekin Formation. With its ziphodont dentition and large size, this taxon likely occupied a position within the top predator guild in this ecosystem [6]. If correctly interpreted as a top terrestrial predator in the Pekin assemblage, *Carnufex* marks a rare and early instance of crocodylomorphs as top tier predators, a role more typically filled by other large basal archosaurs and later filled by theropod dinosaurs [69,70]. Indeed, ecosystems seem to have been in a state of flux for much of the Triassic, with an abundance of diverse predators available to fill vacated top predator roles [71–73]. Carnivorous pseudosuchians were significantly morphologically disparate at this time, and a variety of them were able to successfully invade top predator niches [1,7,74]. Further study into the biology of the basalmost crocodylomorphs and their close relatives will prove essential to understanding the factors that contributed to the survival of crocodylomorphs after the end-Triassic extinction and their subsequent radiation during the Jurassic.

## Supporting Information

S1 File Discussion of Operational Taxonomic Units. (DOCX)

S2 File Character list with discussion of select characters. (DOCX)

S3 File Text file of phylogenetic data matrix. (DOCX)

S4 File Nexus file of phylogenetic data matrix. (NEX)
