# Supplementary material for: Osteology of Carnufex carolinensis (Archosauria: Psuedosuchia) from the Pekin Formation of North Carolina and Its Implications for Early Crocodylomorph Evolution
Source: PLoS One. 2016 Jun 15;11(6):e0157528. doi: 10.1371/journal.pone.0157528 (PMC4909254; doi:10.1371/journal.pone.0157528)
Supplement: S1 File — (DOCX) [file pone.0157528.s001.docx]

**S1 File. Discussion of Operational Taxonomic Units.**

A total of 41 operational taxonomic units (OTUs) were included in this analysis. Taxa taken from the Nesbitt (2011) data set include all taxa found to be members of Paracrocodylomorpha (29 total) and 8 non-paracrocodylomorph basal archosaurs. *Ticinosuchus ferox*, although posited as the sister taxon to Paracrocodylomorpha and not member of the clade by Nesbitt (2011), was included in the ingroup since its position outside Paracrocodylomorpha is only weakly supported (only 2 steps are required to move *Ticinosuchus* into the clade) and additional data may indeed support *Ticinosuchus* as a member of Paracrocodylomorpha (Nesbitt, 2011). A total of 7 outgroup taxa, representing numerous basal archosaur clades, were chosen. These consist of *Euparkeria* *capensis*, *Machaeroprosopus* *pristinus*, *Riojasuchus* *tenulsceps*, *Revueltosaurus* *callenderi*, *Stagonolepis* *robertsoni*, *Turfanosuchus dabanensis*, and *Gracilisuchus* *stipanicicorum*. These taxa were chosen because *Euparkeria* and *Machaeroprosopus* were found as proximal outgroup taxa to Archosauria and the other five were found as basal members of Pseudosuchia, with their relationships poorly resolved, by Nesbitt (2011). In the analysis by Butler et al. (2014), *Turfanosuchus* and *Gracilisuchus* were found to belong to Gracilisuchidae, the sister clade to *Ticinosuchus* + Paracrocodylomorpha.

Although assigned to “*Pseudopalatus*” previously (Nesbitt, 2011), phytosaurs recently considered to belong to the genus *Pseudopalatus* are now referred to the genus *Machaeroprosopus* due to recent taxonomic revisions (Parker et al., 2013), and so the specimens (U. of Mo. 525 VP, UCMP 27235, UCMP 34249, UCMP V2816, UCMP 34253) coded by Nesbitt (2011) as *Pseudopalatus pristinus* are referred to here as *Machaeroprosopus pristinus*. The holotype (FMNH 357) and Yale specimen (YPM 57100) of *Poposaurus gracilis* were scored separately by Nesbitt (2011), but are combined here because Nesbitt (2011) found them to form a clade. Nesbitt (2011) also scored several specimens of *Prestosuchus* separately, but also included a combined *Prestosuchus* OTU; only the combined *Prestosuchus* taxon is included here following the results of Nesbitt (2011), in which all *Prestosuchus* OTUs fell into a polytomy together. The “*Hesperosuchus*” OTU follows Nesbitt (2011) in combining the two specimens (CM 29894 and YPM 41198) from the *Coelophysis* Quarry that have been assigned to *Hesperosuchus agilis*. Discussion of “*Hesperosuchus*” refers to both specimens unless otherwise indicated. It should be noted that the type specimens of *Postosuchus alisonae* and *Dromicosuchus grallator*, originally accessioned at the University of North Carolina, Chapel Hill, are now housed at the North Carolina Museum of Natural Sciences and that the specimen numbers for these type specimens have been changed. The new number for the type specimen of *Postosuchus alisonae* is NCSM 13731 (formerly UNC 15575) and *Dromicosuchus grallator* is now NCSM 13733 (formerly UNC 15574). Four newly added taxa are included in this analysis and are detailed below.

*Decuriasuchus quartacolonia* França, Ferigolo, and Langer 2011

**Age—**Ladinian, Middle Triassic (Abdala and Ribeiro, 2010).

**Occurrence—**“Posts” Site, Alemoa Member, Santa Maria Formation, Rosário do Sul Group, Brazil (Langer et al., 2007).

**Holotype—**MCN PV10105a, articulated partial skeleton.

**Paratype**—MCN PV10105b, nearly complete skeleton lacking skull and forelimb elements; MCN PV10105c, skull and nearly complete postcranial skeleton; MCN PV10105d, skull and presacral vertebrae; MCN PV10105e, trunk and caudal vertebrae, pelvic girdle and hind limbs; MCN PV10105f, distal half of the tail; MCN PV10105g, caudal vertebrae, pelvic girdle, and femora; MCN PV10105h, caudal vertebrae, pelvic girdle, and hind limbs; MCN PV10105i, presacral vertebrae. MCN PV10004, partial skull.

**Remarks**—With a total of ten specimens representing nearly the entire skeleton (França et al., 2011), *Decuriasuchus* is the most complete prestosuchid (*sensu* Brusatte et al., 2010a) currently known, making it an important addition to a paracrocodylomorph dataset. Thorough descriptions of *Decuriasuchus* from the literature (França et al., 2011; França et al., 2013) were used to code this taxon.

**Key References—**França et al., 2011; França et al., 2013.

*Redondavenator quayensis* Nesbitt, Irmis, Lucas, and Hunt, 2005

**Age—**Norian/Rhaetian, Late Triassic.

**Occurrence—**NMMNH locality 2671, uppermost Redonda Formation, USA.

**Holotype—** NMMNH P-25615, premaxillae and anterior portions of nasals and maxillae, incomplete pectoral girdle.

**Remarks**—Although *Redondavenator* was one of the first discovered large-bodied basal crocodylomorph, its phylogenetic position has yet to be tested. This is likely due to the fact that only the snout and a partial shoulder girdle are known, severely limiting the number of characters that can be coded for this taxon. Nesbitt et al. (2005) listed several characteristics in support of *Redondavenator’s* position at the base of Crocodylomorpha. The size of the type specimen (minimum skull length = 60cm) and its suggested phylogenetic position makes *Redondavenator* an important addition to this dataset and point of comparison with *Carnufex carolinensis*.

**Key References—**Nesbitt et al., 2005.

*Carnufex carolinensis* Zanno, Drymala, Nesbitt, and Schneider 2015

**Age—**~231 Ma, Carnian, Late Triassic.

**Occurrence—**NCPALEO 1902 locality, Pekin Formation, Chatham Group, Deep River Basin, Newark Supergroup, USA.

**Holotype**—NCSM 21558, several elements of the skull and postcranial skeleton.

**Referred Material**—NCSM 21623, distal humerus.

**Remarks –** As a large-bodied taxon, exhibiting a mosaic of traits present across Paracrocodylomorpha, this is a critical taxon for examining the early divergence of Crocodylomorpha. Although highly autapomorphic, several elements (e.g. premaxilla, articular, humerus) are phylogenetically informative and capable of placing this new taxon in a solid phylogenetic context.

**Key References—**Zanno et al., 2015

*Junggarsuchus sloani* Clark, Xu, Forster, and Wang, 2004

**Age—** Bathonian–Callovian, Middle Jurassic.

**Occurrence—** Lower Shishugou Formation, China.

**Holotype—** IVPP V14010, a nearly complete skull and anterior half of the postcranial skeleton.

**Remarks**—Although Clark et al. (2004) recovered *Junggarsuchus* as the sister taxon to Crocodyliformes, this taxon bears certain similarities with NCSM 21558, such as a strong ridge on the lateral surface of the angular*.* In addition to certain shared characters with NCSM 21558, *Junggarsuchus* was included in this study because I was able to examine a cast of the holotype skull in person, scoring many characters directly.

**Key References—**Clark et al., 2004.

**References**

Abdala NF, Ribeiro AM. Distribution and diversity patterns of Triassic cynodonts (Therapsida, Cynodontia) in Gondwana. Palaeogeography, Palaeoclimatology, Palaeoecology. 2010; 286: 202–217.

Butler RJ, Sullivan C, Ezcurra MD, Liu J, Lecuona A, Sookias RB. New clade of enigmatic early archosaurs yields insights into early pseudosuchian phylogeny and biogeography of the archosaur radiation. BMC Evolutionary Biology. 2014. doi:10.1186/1471-2148-14-128

Clark JM, Xing X, Forster CA, Wang Y. A Middle Jurassic ‘sphenosuchian’ from China and the origin of the crocodylian skull. Nature. 2004; 430: 1021–1024.

França MAG, Ferigolo J, Langer MC. Associated skeletons of a new middle Triassic ‘Rauisuchian’ from Brazil. Naturwissenschaften. 2011; 98: 389–395.

França MAG, Langer MC, Ferigolo J. The skull anatomy of *Decuriasuchus quartacolonia* (Pseudosuchia: Suchia: Loricata) from the middle Triassic of Brazil. In: Nesbitt SJ, Desojo JB, Irmis RB, editors. Anatomy, Phylogeny and Palaeobiology of Early Archosaurs and their Kin. Geological Society of London Special Publication 379; 2013. pp. 469-501

Nesbitt SJ. The early evolution of archosaurs: relationships and the origin of major clades. Bulletin of the American Museum of Natural History. 2011; 352: 1–292.

Nesbitt SJ, Irmis RB, Lucas SG, Hunt AP. A giant crocodylomorph from the upper Triassic of New Mexico. Paläontolgische Zeitschrift. 2005; 79: 471–478.

Parker WG, Hungerbühler A, Martz JW. The taxonomic status of the phytosaurs (Archosauriformes) *Machaeroprosopus* and *Pseudopalatus* from the Late Triassic of the western United States. Earth and Environmental Science Transactions of the Royal Society of Edinburgh. 2013; 103: 265-268.

Zanno LE, Drymala S, Nesbitt SJ, Schneider VP. Early crocodylomorph increases top tier predator diversity during rise of dinosaurs. Scientific Reports. 2015; 5: 9276. doi:10.1038/srep09276
