# Supplementary material for: Osteology of Carnufex carolinensis (Archosauria: Psuedosuchia) from the Pekin Formation of North Carolina and Its Implications for Early Crocodylomorph Evolution
Source: PLoS One. 2016 Jun 15;11(6):e0157528. doi: 10.1371/journal.pone.0157528 (PMC4909254; doi:10.1371/journal.pone.0157528)
Supplement: S2 File — (DOCX) [file pone.0157528.s002.docx]

**S2 File. Character list with discussion of select characters.**

The character list was modified from Nesbitt (2011) to include additional characters from the literature and several newly formulated characters. Modifications (modified characters and revised scorings) to Nesbitt’s (2011) dataset by Butler et al. (2014) were accepted, but the new character used by Butler et al. (2014) was not incorporated. The new character describes the region of the maxilla posterior to the antorbital fenestra and, based on my own observations, it appears that the morphology in this region of the skull – in which the maxilla, lacrimal, and jugal all meet – is far more complicated and often poorly preserved in many suchians and therefore requires more detailed study before the taxa considered here can be properly scored for a character such as the one used by Butler et al. (2014).

Because of the reduced taxonomic scope of this analysis, characters pertaining exclusively to omitted suprageneric taxa (e.g. Dinosauria) were culled or modified based on their relevance to Paracrocodylomorpha. Several character states were reworded for clarity, rearranged based on outgroup membership, and/or combined/divided to better reflect paracrocodylomorph morphological diversity. Several binary characters were reversed so that the state found primarily in the outgroup was properly reflected as (0) and the derived state found in the ingroup was coded as (1). Reversals are indicated within the list of characters to prevent confusion with scorings from Nesbitt (2011). Characters modified beyond simple rewording or reversal of polarity and new characters are indicated with an asterisk and modifications discussed. Characters with modifications beyond rearrangement of character states through the addition/subtraction of states or the combination/division of existing states are listed as “derived from” a character from Nesbitt (2011).

**Cranial Skeleton**

1. Premaxilla, anterodorsal process, length: (0) short; (1) long (Nesbitt, 2011 – char. 1; Nesbitt and Norell, 2006 – char. 75).

The length of the anterodorsal process of the maxilla is determined relative to the main body of the maxilla. A long anterodorsal process is one of the many characters uniting *Effigia* and *Shuvosaurus*.

2. Premaxilla, posterodorsal process, length: (0) short; (1) long (Nesbitt, 2011 – char. 2).

Like the anterodorsal process, the length of the posterodorsal process is determined relative to the main body of the maxilla. A long posterodorsal process is found in the rauisuchids *Postosuchus* *kirkpatricki*, *Polonosuchus*, and *Rauisuchus*, the basal loricatan *Saurosuchus*, and the crocodylomorphs *Redondavenator* and *Sphenosuchus* (Nesbitt, 2011; Nesbitt et al., 2005).

3.* Premaxilla, posterior process, articulation: (0) loose articulation between maxilla and nasal or fits into a slot in the nasal; (1) laterally overlaps maxilla and may or may not fit into a distinct slot; (2) loosely overlaps anterodorsal border between maxilla and nasal; (3) strongly sutured between maxilla and nasal (modified from Nesbitt, 2011 – char. 4).

This character codes for the mode of articulation for the posterior process of the premaxilla. The posterior process rests loosely (unsutured) between the nasal and maxilla in a number of basal archosaurs (e.g. *Euparkeria*). In some basal suchians (e.g. *Revueltosaurus*) the posterior process fits into a slot on the lateral surface of the nasal. Both of these configurations are scored as (0), whereas Nesbitt (2011) scored each as separate states in his character 4. In poposauroids (e.g., *Effigia*, *Arizonasaurus*), the posterior process laterally overlaps the anterior portion of the maxilla, sometimes fitting into a distinct slot (as in *Arizonasaurus*). A finger-like posterior process of the premaxilla that projects posteriorly to overlap the anterodorsal process of the maxilla and/or the adjacent region of the nasal has been identified in a number of “sphenosuchias” and taxa with such a configuration, including *Carnufex* (NCSM 21558), *Redondavenator* (Nesbitt et al., 2005), *Dromicosuchus* (NCSM 13733), “*Hesperosuchus* *agilis*” (CM 29894, YPM 41198), *Sphenosuchus* (Walker, 1990), *Dibothrosuchus* (Wu and Chatterjee, 1993), *Kayentasuchus* (Clark and Sues, 2002), *Litargosuchus* (Clark and Sues, 2002), and *Junggarsuchus* (Clark et al., 2004) are scored as (2). In Crocodyliformes, the posterior process rests in the same region as in basal crocodylomorphs, but becomes strongly sutured between the maxilla and nasal and these taxa are scored as (3) following Nesbitt (2011) and Clark et al. (2000).

4.* Premaxilla, teeth: (0) present; (1) absent (Nesbitt and Norell, 2006 – char. 73).

Within Paracrocodylomorpha, *Lotosaurus*, *Shuvosaurus*, and *Effigia* all lack dentition in the premaxilla. The absence of teeth in the premaxilla was scored as character (4) within Nesbitt’s (2011) “premaxillary teeth, number” character, but is separated here as a discrete character following Nesbitt and Norell (2006).

5.* Premaxillary teeth number: (0) 3; (1) 4; (2) 5; (3) 6; (4) 7+. ORDERED (modified from Nesbitt, 2011 – char. 6).

This character has been slightly modified from Nesbitt’s (2011) character for premaxillary teeth number to better reflect the number of teeth in the premaxillae of basal crocodylomorphs. *Carnufex carolinensis* (NCSM 21558) unequivocally possesses six premaxillary alveoli. It is also possible that *Dromicosuchus* (NCSM 13733) and *Sphenosuchus* (Walker, 1990) have six premaxillary teeth instead of five, although the degree of crushing in known specimens makes this difficult to diagnose and herein are code them as having five. With the elimination of a state for no teeth (which is now a separate character) in the premaxilla, the state is now ordered.

6. Premaxilla, narial fossa: (0) absent or shallow; (1) distinct, expanded in the anteroventral corner of the naris (Nesbitt, 2011 – char. 9).

An expanded narial fossa on the anteroventral corner of the naris has typically been associated almost exclusively with Saurischia (Langer and Benton, 2006; Irmis et al., 2007). However, Nesbitt (2011) also scored *Batrachotomus* (Gower, 1999) as possessing an expanded narial fossa. França et al. (2013) identified an expanded narial fossa in *Decuriasuchus quartacolonia*, suggesting that this character may be more prevalent within basal Loricata than previously expected.

7. Premaxilla-maxilla, subnarial gap between the two elements in lateral view: (0) absent; (1) present (Nesbitt, 2011 – char. 11).

Many basal crocodylomorphs possess a gap between the maxilla and premaxilla that accommodates an enlarged dentary tooth. This character currently scores only for the presence or absence of such a gap, although it occurs in several different forms. In many basal crocodylomorphs, such as *Redondavenator* (Nesbitt et al., 2005) and *Sphenosuchus* (Walker, 1990), the lateral surface between the maxilla and premaxilla is indented medially and there is a small diastema between the teeth of the two elements to accommodate a large dentary tooth. In *Protosuchus* *richardsoni* (MCZ 6727), a large opening extending for the entire dorsoventral height between the two elements accommodates the fourth dentary tooth. *Dibothrosuchus* (Wu and Chatterjee, 1993) has a large fenestra along the margin between the maxilla and premaxilla, but the fenestra does not interrupt the alveolar margin.

Nesbitt (2011) distinguished between the presence or absence of a subnarial notch to accommodate the fourth dentary tooth (char. 11) and an enclosed subnarial fenestra to accommodate the fourth dentary tooth (Char. 12). The latter character has a wide distribution in a number of archosaur phylogenies (Nesbitt, 2011; Benton and Clark, 1988; Parrish, 1993; Juul, 1994; Benton, 1999). Nesbitt (2011) discusses the subnarial fenestra at length, suggesting that several instances of a foramen or “slit like gap” in “rauisuchians” were taphonomic features resulting from a loose articulation between the two elements, rather than true morphology. Juul (1994) and Gower (2000) also questioned the validity of the subnarial foramen as a character supporting “Rauisuchia”. Personal examination of several basal crocodylomorphs suggests that many purported instances of a subnarial foramen in this clade are also preservational rather than valid morphological features. In many cases (e.g. NCSM 13733, CM 29894), crushing and other taphonomic processes, along with evolutionary modifications to the morphology for the accommodation of the fourth dentary tooth obscure any clear evidence for a foramen between the maxilla and premaxilla. Because preservation often precludes our ability to discriminate between a subnarial notch and foramen (if the premaxilla and maxilla have separated postmortem or been crushed together) and these conditions serve the same function, occurring in closely related taxa, they are considered homologous and are herein combined into a single character state (1).

8. Maxilla, anterior portion: (0) short; (1) long. (Nesbitt, 2011 – char. 14; Clark et al., 2000 – char. 2).

This character describes the relative proportions of the maxilla when split anteriorly and posteriorly from the anterior extent of the antorbital fenestra. Taxa with the facial portion of the maxilla (the portion anterior to the antorbital fenestra) longer than the posterior process (the portion of the maxilla extending posteriorly from the anteriormost extent of the anotorbital fenestra), such as *Sphenosuchus* (Walker, 1990), are scored (1).

Nesbitt et al. (2005) discussed an increased number of teeth (five or more) in the facial portion of the maxilla as a characteristic of “sphenosuchians”. In evaluating this as a potential character, I determined that all taxa possessing five or more maxillary alveoli anterior to the antorbital fenestra also possess an elongate anterior portion of the maxilla. Therefore, taxa with only the anterior portion of the maxillae preserved (e.g. *Redondavenator*) can tentatively be scored for this character. The partial left maxilla of *Redondavenator* preserves six alveoli and is therefore scored as (1). In continued analysis of archosauriforms, it is possible that these features will not always correlate, so it is important that both tooth number and relative length be observed in the anterior portion of the maxilla for all future coding; the two features may need to be developed into two separate characters.

9. Posterior maxillary teeth, posterior edge: (0) concave or straight; (1) convex (Nesbitt, 2011 – char. 15; modified from Sues et al., 2003 – char. 28).

In many carnivorous archosaurs, recurved teeth are present throughout the tooth row. Within Crocodylomorpha, the posterior maxillary teeth, become more bulbous and mesodistally symmetrical, resulting in a convex posterior edge.

10. Maxilla, dentition in posterior portion: (0) present; (1) absent (Nesbitt, 2011 – char. 17).

This character is not currently informative for early-Mesozoic paracrocodylomorphs, because only *Orthosuchus* (Nash, 1975) is scored (1) in the current analysis.

11. Maxilla, dentition: (0) present; (1) absent (Nesbitt, 2011 – char. 18; Nesbitt and Norell, 2006 – char. 74).

12.* Maxilla, anterior alveoli: (0) all alveoli approximately the same size; (1) first alveolus much smaller than others; (2) first two alveoli much smaller than others (**new**). ORDERED.

The first two maxillary alveoli are reduced in several basal crocodylomorphs, including *Redondavenator*, *Dibothrosuchus*, *Sphenosuchus*, and *Terrestrisuchus* (Nesbitt et al., 2005), as well as *Qianosuchus* (Li et al., 2006). Only the first maxillary alveolus is reduced in *Postosuchus kirkpatricki* and *Batrachotomus*. Other paracrocodylomorph taxa show no pattern in the size of the anterior maxillary alveoli.

13. Maxilla, posteroventral portion (ventral to the lacrimal): (0) thin (dorsoventral height less than the mediolateral width); (1) thick (mediolateral length greater than dorsoventral height) (Nesbitt, 2011 – char. 21).

14. Maxilla, interdental plates: (0) individually distinct, separate from each other; (1) fused to each other along medial side of tooth row (Nesbitt, 2011 – char. 22).

This character has been slightly reworded from Nesbitt (2011) to clarify the specific morphology being coded for. While many archosaurs possess interdental plates that are fused to the maxilla, several “rauisuchians” (e.g. *Postosuchus* *kirkpatricki*, *Polonosuchus*, *Fasolasuchus*) have fused the interdental plates to one another along the medial side of the tooth row, creating a continuous sheet of bone.

15. Maxilla, anterodorsal margin: (0) separated from the external naris by the premaxilla; (1) borders the external naris (Nesbitt, 2011 – char. 24; modified from Gauthier, 1986)

16. Maxilla, anterodorsal margin: (0) convex or straight; (1) concave (Nesbitt, 2011 – char. 25; modified from Langer and Benton, 2006 – char. 6).

17. Maxilla, lateral surface: (0) smooth; (1) sharp longitudinal ridge; (2) bulbous longitudinal ridge (Nesbitt, 2011 – char. 26; Weinbaum and Hungerbühler, 2007 – char. 2).

18.* Maxilla, posterior process ventral to the antorbital fenestra, not including the posterior-most end of the maxilla: (0) becomes dorsoventrally deeper posteriorly; (1) tapers posteriorly; (2) approximately constant in dorsoventral depth (states rearranged from Butler et al., 2014; modified from Nesbitt, 2011 – char. 27).

Butler et al. (2014) revised this character to better describe the majority of the posterior process of the premaxilla without the influence of the articulation with the jugal. Butler et al. (2014) noticed that the posterior-most extent of the posterior process, ventral to the lacrimal, expands into a triangular posterodorsal process in some taxa and created a new character (413) for this morphology. This new character pertains primarily to Gracilisuchidae and so is not included here. However, my own observations suggest that the articulation between the posterior process of the maxilla and the jugal (and lacrimal) may be more complicated than previously suspected in a number of basal crocodylomorph specimens (e.g. NCSM 21558, NCSM 13733, YPM 41198, CM 29894). Due to the fragile nature of this articulation—generally resulting in poor preservation—and subsequent difficulty in diagnosing different potential character states, a new character for the articulation of the maxilla and jugal is not included herein. Instead, the modifications to Nesbitt’s (2011) character by Butler et al. (2014), evaluating changes in the dorsoventral height of the posterior process of the maxilla ventral to the antorbital fenestra, are accepted here. The states listed by Butler et al. (2014) have been rearranged to reflect the states seen in the outgroup versus the ingroup.

19. Maxilla, ascending process: (0) tapers posterodorsally; (1) remains the same width for its length, with a nearly vertical suture with the lacrimal (Nesbitt, 2011 – char. 29).

20. Antorbital fenestra, anterior margin: (0) gently rounded; (1) nearly pointed (Nesbitt, 2011 – char. 30; modified from Benton and Clark, 1988).

21. Maxilla, anterolateral surface, large anteriorly opening foramen: (0) present; (1) absent (Nesbitt, 2011 – char. 31; Modesto and Sues, 2004 – char. 17).

22.* Maxilla, palatal processes: (0) do not meet at the midline; (1) small, meet at the midline; (2) large, meet at the midline and expand anteriorly and posteriorly, but extend only from the anterior portion of the maxilla; (3) large, meet at the midline and expand anteriorly and posteriorly to such an extent that they prevent the palatine from meeting the body of the maxilla. ORDERED (expanded from Nesbitt, 2011 – char. 32; Clark et al. 2000 – char. 3).

Early crocodylomorph evolution suggests a gradual expansion of the secondary palate (Irmis et al., 2013). In order to better represent the diverse morphology exhibited during the expansion of the palatal process of the maxilla in early crocodylomorph evolution, Nesbitt’s original state (2) has been divided into two characters to illustrate the degree of anteroposterior expansion, especially the expansion of the lateralmost portion of the palatal process, along the medial face of the main body of the maxilla, just dorsomedial to the tooth row. In *Sphenosuchus* (Walker, 1990), the lateral extent of the palatal process runs from just posterior to the second maxillary alveolus to the seventh. Medially, the palatal process constricts anteroposteriorly to less than 3 alveoli in length, before expanding once again along the midline where it meets it’s antimere. The lateral arm of the palatine expands from the seventh maxillary alveolus, along the remaining posterior length of the maxilla. A similar configuration is found in most other basal crocodylomorphs. In more derived crocodyliforms (e.g. *Alligator*), the palatal process of the maxilla expands further posteriorly along its full mediolateral width, preventing the palatine from meeting the main body of the maxilla.

23. Nasals, posterior portion at the midline: (0) convex or flat; (1) concave (Nesbitt, 2011 – char. 34; Brusatte et al., 2010 – char. 27).

24. Nasal, dorsolateral margin of the anterior portion: (0) smoothly rounded; (1) distinct anteroposteriorly trending ridge on lateral edge (Weinbaum and Hungerbühler, 2007 – char. 3; Brusatte et al., 2010 – char. 26; Nesbitt, 2011 – char. 35).

25. Nasal, participation in the dorsal boarder of the antorbital fossa: (0) absent; (1) present (Nesbitt, 2011 – char. 37; modified from Sereno et al., 1994)

26.* Nasal, anterior portion, descending process: (0) present; (1) absent (**new**).

A descending process of the nasal, which typically projects anteroventrally to form a portion of the posterior border of the external naris, has been described for numerous pseudosuchian taxa, such as *Riojasuchus* (Bonaparte, 1967) and *Postosuchus kirkpatricki* (Weinbaum, 2011), but has not been used as a character in evaluating these taxa until now. Ingroup taxa coded as (1) include all Rauisuchids except for *Polonosuchus* and all crocodylomorphs for which the anterior portion of the nasal is preserved.

27.* Lacrimal, shape: (0) roughly square or slightly rectangular, descending process highly reduced or absent; (1) tall and slender, anterior process may or may not be present; (2) L-shaped with distinct anterior and descending processes (**new**).

A lacrimal that appears rectangular in lateral view appears in many basal pseudosuchians, phytosaurians, in the rauisuchids *Postosuchus* *kirkpatricki* and *Polonosuchus*, and in *Alligator*. *Xilousichus*, *Lotosaurus*, *Shuvosaurus*, and *Effigia* possess an extremely slender and tall lacrimal. A plate-like, L-shaped process in present in Gracilisuchidae, *Qianosuchus*, and all non-rauisuchid loricatans included in the analysis, except for *Alligator*.

28. Prefrontal, ventromedial process: (0) absent; (1) present (Nesbitt, 2011 – char. 40; Clark et al., 2000 – char. 5).

29. Prefrontal, contact with palate: (0) absent; (1) present (Nesbitt, 2011 – char. 41; Wu and Chatterjee, 1993 – char. 10).

30. Frontal, dorsal surface: (0) flat; (1) longitudinal ridge along midline (Nesbitt, 2011 – char. 42; Wu and Chatterjee, 1993 – char. 11).

31.* Frontal, participation in the dorsolateral border of the orbit: (0) present; (1) absent (**new**).

In most paracrocodylomorphs, the frontal contributes to the dorsolateral border of the orbit. In *Prestosuchus* (Parrish, 1993), *Saurosuchus* (Alcober, 2000; Nesbitt, 2011), *Postosuchus* *kirkpatricki* (Weinbaum, 2011), and *Polonosuchus* (Sulej, 2005), the frontal is excluded from participation in the orbital margin by a combination of the prefrontal, palpebral, and/or postfrontal.

32. Frontal, anterior margin: (0) about as wide as at the orbital margin or has a transversely aligned suture with the nasal; (1) tapers anteriorly along the midline (Nesbitt, 2011 – char. 43).

33. Postfrontal: (0) present; (1) absent (Nesbitt, 2011 – char. 44; Gauthier, 1986).

34. Quadratojugal: (0) forms less than 80% of the posterior border of the lower temporal fenestra; (1) forms more than 80% of the posterior border of the lower temporal fenestra (Nesbitt, 2011 – char. 45; modified from Benton and Clark, 1988).

35. Squamosal, posterior end: (0) extends posterior to the head of the quadrate; (1) does not extend posterior to the head of the quadrate (Nesbitt, 2011 – char. 48; Nesbitt et al. 2009 – char. 14).

36. Squamosal, distinct ridge on dorsal surface along edge of supratemporal fossa: (0) absent; (1) present (Nesbitt, 2011 – char. 49; Bonaparte, 1982).

37. Squamosal, dorsal exposure: (0) restricted – mediolateral width less than that of the upper temporal fenestra; (1) broad – mediolateral width greater than that of the upper temporal fenestra (Nesbitt, 2011 – char. 50).

38. Squamosal, ridge on lateral side of ventral process: (0) absent; (1) present (Nesbitt, 2011 – char. 51).

39. Squamosal, anteroventral process: (0) absent; (1) present and perforates the lower temporal fenestra; (2) present and contacts the postorbital/jugal, bisecting the lower temporal fenestra. ORDERED (Nesbitt, 2011 – char. 52).

40. Squamosal, dorsolateral edge, longitudinal groove: (0) absent; (1) present (Nesbitt, 2011 – char. 53; Clark et al., 2000 – char. 12).

41. Squamosal, posterodorsal portion, upper temporal fenestra: (0) absent; (1) present (Nesbitt, 2011 – char. 55).

42. Squamosal, ventral process: (0) wider than one-quarter its length; (1) narrower than one-quarter its length (Nesbitt, 2011 – char. 56; Yates, 2003).

43. Squamosal, deep pit on the posterodorsal corner of the lateral surface: (0) absent; (1) present (Nesbitt, 2011 – char. 57; Brusatte et al., 2008; Brusatte et al., 2010 – char. 50).

44.* Squamosal, broad lateral expansion overhanging lateral temporal region: (0) absent; (1) present (Pol et al., 2013 – char. 10).

45. Parietals, in presumed adults: (0) separate; (1) fused into one bone (Nesbitt, 2011 – char. 58; Clark et al., 2000 – char. 15).

46. Area between upper temporal fenestra created by parietals: (0) broad, flat; (1) mediolaterally thin strip; (2) "sagittal crest" (Nesbitt, 2011 – char. 59; modified from Clark et al., 2000 – char. 17).

47. Parietals, posteroventral edge: (0) extends more than half the width of the occiput; (1) extends less than half the width of the occiput (Nesbitt, 2011 – char. 60; Clark et al., 2000 – char. 16).

48. Parietals, occipital margin shape in dorsal view: (0) V-shaped; (1) straight (Nesbitt, 2011 – char. 61; Clark et al, 2000 – char. 18).

49. Quadratojugal - postorbital contact: (0) absent; (1) present (Nesbitt, 2011 – char. 64; Parrish, 1991 – char. 51)

Although this character has been used in a number of analyses (Parrish, 1991, 1993; Nesbitt, 2011), the full distribution of this character seems to be much more extensive within Crocodylomorpha than previously recognized. The quadratojugals in many basal crocodylomorph specimens have been crushed and as Nesbitt (2011) observed, since scoring of this character requires excellent preservation, many taxa have not been previously scored. Observation of a number of basal crocodylomorph specimens (*Dromicosuchus*: NCSM 13733, “*Hesperosuchus*”: CM 29894, and at least two undescribed taxa) suggests that the morphology of the quadratojugal in basal crocodylomorphs is more derived than expected. The quadratojugal in these taxa appears to be exceptionally thin, anteriorly inclined at a low angle, and covering much of the area thought to be occupied by the infratemporal fenestra, suggesting a small slit like infratemporal fenestra just above the posterior process of the jugal. Nesbitt (2011) scored *Dromicosuchus*, “*Hesperosuchus* *agilis*”, and *Sphenosuchus* as (0). Based on these new observations, I rescore them as (1).

50. Postorbital, ventral termination of ventral process: (0) tapered; (1) blunt (Nesbitt, 2011 – char. 65; modified from Benton and Clark, 1988).

51. Postorbital-squamosal contact: (0) restricted to the dorsal margin of the elements; (1) continues for much of the ventral length of the squamosals (Nesbitt, 2011 – char. 66).

52. Postorbital bar: (0) composed of both the jugal and postorbital in equal proportions; (1) composed mostly of the postorbital (Nesbitt, 2011 – char. 67).

53. Jugal, anterior process: (0) participates in the posterior edge of the antorbital fenestra; (1) excluded from the antorbital fenestra by the lacrimal or maxilla (Nesbitt, 2011 – char. 69; Clark et al., 2000 – char. 4).

54.* Jugal, posterior process: (0) rises dorsal to or splits the anterior process of the quadratojugal; (1) extends ventral to the anterior process of the quadratojugal (modified from Nesbitt, 2011 – char. 71).

In many basal archosaurs, the posterior process of the jugal either extends dorsal to the anterior process of the quadratojugal or splits it. These states were originally illustrated by Nesbitt (2011) as states (0) and (2) respectively. Here, the two states have been combined into state (0), since the only variability between the two configurations is seen in the outgroup. More derived loricatans, including *Postosuchus kirkpatricki*, *Polonosuchus*, *Carnufex* and Crocodylomorpha, possess a jugal with a posterior process that lies ventral to the anterior process of the quadratojugal.

55. Jugal, posterior termination: (0) does not extend posterior to the lower temporal fenestra; (1) extends posterior to the lower temporal fenestra (Nesbitt, 2011 – char. 72).

56. Jugal, posterior border of the postorbital process: (0) concave; (1) convex (Nesbitt, 2011 – char. 73).

57.* Jugal, longitudinal ridge on the lateral surface of the body: (0) absent; (1) present; (2) present and forms a bulbous ridge (Butler et al., 2014 – char. 75; modified from Nesbitt, 2011 – char. 75).

Butler et al. (2014) modified this character by combining states (1) and (2) of Nesbitt (2011), which described a “sharp” versus a “rounded” ridge respectively, due to the ambiguity between the two types of ridge. In addition, Nesbitt’s (2011) state (1) does not apply to any paracrocodylomorph taxa. For both reasons, the modifications made by Butler et al. (2014) are followed here.

58. Quadrate-prootic contact: (0) absent; (1) present (Nesbitt, 2011 – char. 76; Walker, 1990).

59. Quadrate, head: (0) partially exposed laterally; (1) completely covered by the squamosal (Nesbitt, 2011 – char. 78; Sereno and Novas, 1992).

60. Quadrate foramen, between quadratojugal and quadrate: (0) present; (1) absent (Nesbitt, 2011 – char. 79; modified from Parrish, 1991 – char. 4).

61. Quadrate body, fenestrae: (0) absent; (1) present (Nesbitt, 2011 – char. 80; Clark et al., 2004 – char. 46).

62. Quadrate, angled: (0) posteroventrally or vertical; (1) anteroventrally (Nesbitt, 2011 – char. 82; Nesbitt, 2007).

63. Quadrate, dorsoventrally oriented crest located on the posterior side: (0) absent; (1) present (Nesbitt, 2011 – char. 83).

64. Ectopterygoid: (0) single-headed; (1) double-headed (Nesbitt, 2011 – char. 89; Weinbaum and Hungerbuhler, 2007 – char. 7).

65. Palatine, fossa on the dorsal surface: (0) extends far anteriorly, near the pila postchoanalis; (1) does not extend far anteriorly along the upper surface of the palatine (Nesbitt, 2011 – char. 90; Witmer, 1997).

66. Palatine, posterior margin of choana, raised rim defining a fossa around the choana: (0) absent; (1) present (Nesbitt, 2011 – char. 91).

67. Basipterygoid, process directed: (0) posteriorly at their distal tips; (1) anteriorly or ventrally at their distal tips (reversed from Nesbitt, 2011 – char. 93).

68*. Parabasisphenoid, foramina for the entrance of the cerebral branches of the internal carotid artery into the braincase positioned on the surface: (0) ventral; (1) anterolateral (modified from Nesbitt, 2011 – char. 95; modified from Parrish, 1993 – char. 7).

Nesbitt’s (2011) original state (1) – posterolateral – has been eliminated here, as it only applies to phytosaurs.

69. Parabasisphenoid, plate: (0) absent; (1) present (reversed from Gower and Sennikov, 1996; Nesbitt et al., 2009 – char. 21).

70. Parabasisphenoid, basipterygoid process: (0) present; (1) absent (Nesbitt, 2011 – char. 99; Clark et al., 2000 – char. 24).

71. Parabasisphenoid, recess: (0) absent; (1) present (Nesbitt, 2011 – char. 100; modified from Nesbitt and Norell, 2006 – char. 76).

72. Parabasisphenoid, between basal tubera and basipterygoid processes: (0) approximately as wide as long or wider; (1) significantly elongated, at least 1.5 times longer than wide (Nesbitt, 2011 – char. 103; Rauhut, 2003).

73. Prootic-opisthotic, contact: (0) broad overlap; (1) reduced to a small contact (Nesbitt, 2011 – char. 105; modified from Clark et al., 2000 – char. 20).

74. Basioccipital, portion of the basal tubera: (0) rounded and anteroposteriorly elongated; (1) bladelike and anteroposteriorly shortened (Nesbitt, 2011 – char. 106).

75. Basioccipital, deep recess on the ventral surface: (0) absent; (1) present (Nesbitt, 2011 – char. 107).

76. Opisthotic, paraoccipital process: (0) no or slight dorsal and ventral expansion distally; (1) markedly expanded dorsally at the distal end (Nesbitt, 2011 – char. 108; reversed from Clark et al., 2000 – char. 23).

77. Opisthotic, extent of the lateral margin of the paraoccipital: (0) lateral to the upper temporal fenestra; (1) at the margin or medial to the lateral extent of the upper temporal fenestra (Nesbitt, 2011 – char. 109).

78. Opisthotic, paraoccipital process: (0) directed laterally or dorsolaterally; (1) directed ventrolaterally (Nesbitt, 2011 – char. 110; Rauhut, 1997).

79. Opisthotic, ventral ramus: (0) extends further laterally than the lateralmost edge of exoccipital in posterior view; (1) covered by the lateralmost edge of the exoccipital in posterior view (Nesbitt, 2011 – char. 111; Gower, 2002 – char. 3)

80. Opisthotic, distal end of the ventral ramus: (0) does not or barely makes contact with prootic anteroventral to fenestra ovalis; (1) has extended contact with prootic (Nesbitt, 2011 – char. 112; Gower, 2002 – char. 12).

81. Exoccipital, lateral surface, subverticle crest (=metotic strut): (0) absent; (1) present, lying anterior to both external foramina for hypoglossal nerve (XII); (2) present, anterior to the more posterior external foramina for hypoglossal nerve (XII) (Nesbitt, 2011 – char. 114; modified from Gower, 2002 – char. 2).

82. Exoccipitals: (0) meet along the midline on the floor of the endocranial cavity; (1) do not meet along the midline on the floor of the endocranial cavity (Nesbitt, 2011 – char. 115; modified from Gower and Sennikov, 1996 – char. 17).

83. Pneumatization of the bony elements of the middle ear cavity: (0) absent or restricted; (1) well developed (Nesbitt, 2011 – char. 116; Gower, 2002 – char. 6).

84. Vestibule, medial wall: (0) incompletely ossified; (1) almost completely ossified (Nesbitt, 2011 – char. 117; Gower, 2002 – char. 7).

85. Crista vestibule: (0) absent; (1) present (Nesbitt, 2011 – char. 119; Gower, 2002 – char. 10).

86. Lagenar/cochlear prominence: (0) absent; (1) present (Nesbitt, 2011 – char. 120; Gower, 2002 – char. 11).

87. Eustachian tubes: (0) not enclosed by bone; (1) partially enclosed by bone; (2) fully enclosed by bone. ORDERED (Nesbitt, 2011 – char. 121; Gower, 2002 – char. 13).

88. External foramen for abducen nerve: (0) between parabasisphenoid and prootic; (1) within prootic; (2) within parabasisphenoid (Nesbitt, 2011 – char. 122; Gower, 2002 – char. 15).

89. Basipterygoid process: (0) of moderate size; (1) markedly enlarged (Nesbitt, 2011 – char. 124; Gower, 2002 – char. 18).

90. Exit of cranial nerve VII: (0) small, only slightly larger than cranial nerve XII; (1) large (Nesbitt, 2011 – char. 125).

91. Supraoccipital: (0) contributes to border of foramen magnum; (1) excluded from dorsal border of foramen magnum by mediodorsal midline contact between opposite exoccipitals (**reversed** from Nesbitt, 2011 – char. 126; Gower, 2002 – char. 19).

92. Pila antotica: (0) ossified mainly by prootic and laterosphenoid, such that laterosphenoid-parabasisphenoid contact is absent (1) ossified largely by the laterosphenoid and parabasisphenoid, with contact occurring between these two elements anterior to the trigeminal foramen in the adult braincase (Nesbitt, 2011 – char. 128; Gower, 2002 – char. 20).

93. Perilymphatic foramen: (0) with an incompletely ossified border; (1) entirely ossified such that the ventral ramus of the opisthotic forms a perilymphatic loop incorperating a loop closure suture with itself (Nesbitt, 2011 – char. 129; Gower, 2002 – char. 21).

94. Perilymphatic foramen: (0) in a medial position and oriented so as to transmit the perilymphatic duct out if the otic capsule in a posteromedial or posterior direction; (1) foramen positioned more laterally so that the perilymphatic duct is transmitted posterolaterally/ laterally and the foramen is at least partly visible in lateral view (Nesbitt, 2011 – char. 130; Gower, 2002 – char. 22).

95.* Foramen for trigeminal nerve and middle cerebral vein: (0) combined and undivided; (1) at least partially subdivided by prootic (**modified** from Nesbitt, 2011 – char. 131; Gower, 2002 – char. 23).

Nesbitt’s (2011) original state 2 has been eliminated, since the foramen for the trigeminal nerve and middle cerebral vein is only fully divided in some saurischian dinosaurs (Rauhut, 2003).

96. Foramen or groove passing above and into the dorsal end of the metotic foramen: (0) absent; (1) present (Nesbitt, 2011 – char. 132; Gower, 2002 – char. 26).

97. Auricular recess: (0) largely restricted to prootic; (1) extends onto internal surface of epiotic/supraoccipital (Nesbitt, 2011 – char. 133; Gower, 2002 – char. 25).

98.* External nares, shape: (0) small (<15% skull length) and subcircular; (1) large (>20% skull length) and ovate; (2) teardrop-shaped, tapers to point posteriorly (**new**).

A reduced external naris has been suggested as a character of “sphenosuchians” (Nesbitt et al., 2005) and a teardrop-shaped naris has been described in a number of basal loricatans (e.g., *Decuriasuchus quartacolonia*, França et al., 2013). However, the shape of the external naris has yet to be used as a character in phylogenetic analyses of these taxa. In order to formulate appropriate states for this character, the nares of all terminal taxa were first grouped into shape categories (elongate teardrop-shaped, coming to a point posteriorly vs. subcircular/ovate), then the ratio of naris length to skull length was calculated. In taxa with teardrop nares (e.g. *Saurosuchus galilei, Postosuchus kirkpatricki*), nearly all had nares that were approximately 15% of the total skull length. Taxa with round nares fell into two discrete categories divisible by a “gap” (Thorpe, 1984; Archie, 1985) in length of about 5% skull length: those with small nares, less than 15% of the total skull length (e.g. *Redondavenator quayensis, Dromicosuchus grallator, Protosuchus richardsoni*) and those with enlarged nares, greater than 20% of the total skull length (e.g. *Batrachotomus kupferzellensis; Xilousuchus sapingensis*).

99.* Antorbital fossa: (0) poorly defined; (1) well defined anterodorsally, yet not well defined along entire length of posterior process of maxilla; (2) well defined, forming complete circumference around the antorbital fenestra (**new**). ORDERED.

Nesbitt (2011) included a character for the extent of the antorbital fossa (character 137). However, it focused on the extent of the fossa alone and did not capture variation in the length of a banked border around the depressed fossa. In a number of basal crocodylomorphs (*Dromicosuchus* *grallator*, YPM 41198, CM 29894, etc.), a clearly defined fossa encircles the entire fenestra, with a deeply recessed area appearing even at the posteroventral corner of the antorbital fenestra at the junction of the maxilla, jugal, and lacrimal. In most other paracrocodylomorphs, such as *Decuriasuchus quartacolonia* (França et al., 2013) and *Sphenosuchus acutus* (Walker, 1990), a sharp border defining the antorbital fossa cannot be identified on the posterior process of the maxilla.

100. Posttemporal opening, mediolateral width: (0) equal to or greater than half the diameter of the foramen magnum; (1) less than half the diameter of the foramen magnum or absent (Nesbitt, 2011 – char. 141; modified from Sereno and Novas, 1994; Novas, 1996; Benton, 1999 – char. 11).

101.* Orbit, shape: (0) circular or elliptical; (1) tall and narrow ("keyhole-shaped orbit"; maximum width is less than half the maximum height) (modified from Nesbitt, 2011 – char. 142; Benton and Clark, 1988).

Nesbitt’s (2011) state (2) – with distinct ventral point surrounded by V-shaped dorsal process of jugal – applies only to ornithosuchids and is eliminated here.

102. Supratemporal fossa: (0) absent anterior to supratemporal fenestra; (1) present anterior to the supratemporal fenestra (Nesbitt, 2011 – char. 144; modified from Gauthier, 1986).

103. Palpebral(s): (0) absent; (1) present (Nesbitt, 2011 – char. 147).

Within Paracrocodylomorpha, when present, only one palpebral is found on each side of the skull, as opposed to multiple palpebrals found in some other suchian taxa (e.g. *Aetosaurus*). When a palpebral is present, it may be firmly sutured to the frontal along the lateral side of the skull roof (e.g. *Postosuchus kirkpatricki*), which is scored for in character 31, or floating within the orbit (CM 29894). Scoring a palpebral as absent (0) requires exceptional preservation in order to differentiate from a palpebral that is simply separated from the lateral edge of the frontals (1) and just not preserved.

104. Dentary, anterior extent of the Meckelian groove: (0) ends well short of the dentary symphysis; (1) present through the dentary symphysis (Nesbitt, 2011 – char. 153).

105.* Dentary, dorsal margin of the anterior portion compared to the to the dorsal margin of the posterior portion: (0) horizontal (in the same plane); (1) dorsally expanded (modified from Nesbitt, 2011 – char. 154).

A dorsally expanded anterior portion of the dentary in found in a number of Triassic loricatans, including *Postosuchus* *kirkpatricki*, *Hesperosuchus* *agilis*, and *Sphenosuchus*, as well as aetosaurians. Nesbitt (2011) also included a character state for a ventrally deflected dentary, found in sauropodomorphs, but this character has been eliminated here.

106. Dentary, anterior extremity: (0) rounded; (1) tapers to a sharp point (Nesbitt, 2011 – char. 155).

107.* Articular, transverse groove posterior to glenoid: (0) absent; (1) present (derived from Nesbitt, 2011 – char. 156).

Character 156 of Nesbitt (2011) and previous authors (Clark et al., 2000; Olsen et al., 2000; Benton and Walker, 2002; Sues et al., 2003; Clark et al., 2004) describes various combinations of two features: a transverse groove and a dorsomedial process in the region of the articular posterior to the glenoid. Although these features appear in the same region, they are morphologically distinct and likely serve different anatomical functions, so they are treated as separated features here. Therefore, the character has been separated into two characters scoring for the presence or absence of the transvers groove (character 107) and dorsomedially directed process (character 108, below) independently.

108.* Articular, slender dorsomedially directed process projecting posteromedial to the glenoid: (0) absent; (1) present, relatively short; (2) present, elongate and "finger-like". ORDERED (derived from Nesbitt, 2011 – char. 156; see above).

109.* Articular, region posterior to glenoid: (0) large, terminating posteriorly as a knob-like process (1) restricted, terminating just posterior to the glenoid with a flat or slightly concave posterior face (modified from Clark, 1994; Pol et al., 2013 – char. 85).

The entire region posterior to the glenoid of the articular in living crocodilians forms a large yet simple posteriorly directed process, termed the “retroarticular process” (Iordansky, 1973). In Triassic archosaurs, the articular tends to be more complicated, with multiple processes in the “retroarticular” region, the term has been used indiscriminately for a number of these processes such as the dorsomedially directed process, as discussed by Gower (1999) and Nesbitt (2011). Here, the term “retroarticular process” is abandoned in favor of a more explicit description. State (0) scores for a true retroarticular process in extant crocodilians (i.e. *Alligator* in this analysis), as well as similarly shaped and oriented processes that project posteriorly from the region behind the glenoid in more basal taxa. Such a process is found in a number of “rauisuchian taxa”, including *Batrachotomus kupferzellensis* (Gower, 1999), *Postosuchus kirkpatricki* (UCMP 27485; PEFO), and *Postosuchus alisonae* (NCSM 13731). In basal crocodylomorphs, no such process is present. Instead, immediately posterior to the dorsomedial ascending process, the articular terminates abruptly as a relatively flat, posterior facing surface. This surface may be vertical or slightly angled, flat or slightly concave, and is generally ovate.

110. Articular, ventromedially directed process: (0) absent; (1) present (Nesbitt, 2011 – char. 157).

Many “rauisuchian” (e.g. *Postosuchus*) taxa possess a “tongue-like” process on the medial side of the articular. *Carnufex* exhibits a more pyramidal process. Both conditions are considered state (1) herein. The process in basal crocodylomorphs is not known because preserved articulars typically derive from articulated skulls, which rarely have their internal surfaces prepared. In the future, as more ventromedial processes are revealed, a character describing the shape of the process may be necessary.

111. Articular, glenoid of the mandible located: (0) level with dorsal margin of the dentary; (1) well ventral to the dorsal margin of the dentary (Nesbitt, 2011 – char. 158; modified from Gauthier, 1986).

112. Articular, foramen on the medial side: (0) present; (1) absent (reversed from Nesbitt, 2011 – char. 159).

113. Dentary-splenial mandibular symphysis, length: (0) distally positioned; (1) present along one-third of the lower jaw (Nesbitt, 2011 – char. 160; Sereno, 1991).

114. Mandibular fenestra: (0) anteroposterior length more than maximum depth of dentary ramus but less than half the length of the mandible; (1) greater than half the length of the mandible; (2) reduced (anteroposterior length less than maximum depth of dentary ramus) (Nesbitt, 2011 – char. 162; Butler, 2005; Nesbitt and Norell, 2006).

115. Angular, ridge on lateral surface posterior to external mandibular fenestra: (0) absent; (1) present (Clark et al., 2004 – char. 44).

A robust, posterodorsal trending ridge on the lateral aspect of the angular, between the articular and the mandibular fenestra, is seen in *Alligator*, *Junggarsuchus*, and *Carnufex*, of the taxa assessed here. This ridge represents the insertion area for the *M. pterygoideus ventralis* (Clark et al., 2004).

116. Surangular foramen: (0) present and small; (1) present and large; (2) absent (Nesbitt, 2011 – char. 163; modified from Clark et al., 2004 – char. 43).

117. Dentary teeth: (0) present; (1) absent (simplified to presence/absence from Nesbitt, 2011 – char. 166; modified from Parrish, 1994).

118. Tooth crown: (0) not mesiodistally expanded; (1) mesiodistally expanded above root in cheek teeth (Nesbitt, 2011 – char. 171; Sereno, 1986).

**Axial Skeleton**

119. Axis, ventral surface: (0) possesses a single midline keel; (1) possesses two paramedian keels (Nesbitt, 2011 – char. 180).

120. Cervical 3-5 centrum length: (0) shorter or the same length as the middorsals; (1) longer than middorsals (Nesbitt, 2011 – char. 181; Sereno, 1991).

121. Third cervical vertebra, centrum length: (0) subequal to the axis centrum; (1) longer than the axis centrum (Nesbitt, 2011 – char. 183; Gauthier, 1986).

122. Postaxial anterior cervical vertebrae, epipophyses: (0) absent; (1) present (Nesbitt, 2011 – char. 186; Gauthier, 1986).

123.* Cervical vertebrae, deep fossae: (0) absent; (1) present (modified from Nesbitt, 2011 – char. 188; modified from Holtz, 1994).

A version of this character has been used to describe pneumatic features in the vertebrae of saurischian dinosaurs, but Nesbitt (2011) also scored the shuvosaurids *Shuvosaurus* and *Sillosuchus* for deep fossae. Such fossae also appear to be present in some loricatans, including *Postosuchus alisonae* (NCSM 13731) and *Carnufex carolinensis* (NCSM 21558). The correlation between vertebral fossae and laminae and the presence of postcranial skeletal pneumaticity or a non-invasive system of pulmonary air sacs, especially among “rauisuchians” is still under debate (Nesbitt et al., 2013), so this character has been reformulated for the presence of fossae alone and not for explicit pneumatic features.

124. Cervical vertebrae, rimmed depression on the posterior part of the centrum: (0) absent; (1) present (Nesbitt, 2011 – char. 189; modified from Gauthier, 1986).

125. Cervical vertebrae, middle portion of ventral keel: (0) dorsal to the ventralmost extent of the centrum rim; (1) extends ventral to the centrum rims (Nesbitt, 2011 – char. 190).

126. Cervical vertebrae, distal end of neural spines: (0) expansion absent; (1) laterally expanded in the middle of the anteroposterior length; (2) expanded anteriorly, so that the spine table is triangular or heart shaped in dorsal view (Nesbitt, 2011 – char. 191; modified from Gauthier, 1984).

127. Middle cervical vertebrae, hypapophyses: (0) absent; (1) present (Nesbitt, 2011 – char. 192).

128. Posterior cervical vertebrae, divided parapophyses: (0) absent; (1) present (Nesbitt, 2011 – char. 193; modified from Weinbaum and Hungerbühler, 2007 - 18).

129. Posterior cervical vertebrae, neural spines: (0) directed dorsally, straight; (1) arc anteriorly (Nesbitt, 2011 – char. 194).

130.* Posterior cervical and/or dorsal vertebrae, hyposphene: (0) absent; (1) present (**modified** from Nesbitt, 2011 – char. 195; Gauthier, 1986).

This character codes specifically for a thin vertical lamina projecting ventrally from between the postzygapophyes, instead of the presence of hyposphene-hypantrum accessory articulations more broadly. *Carnufex carolinensis* does not display typical hyposphene-hypantrum accessory articulations, but hyposphene-like vertical laminae projecting ventrally from the postzygapophyses, and is coded (1).

131. Cervical ribs: (0) slender and elongate; (1) short and stout (Nesbitt, 2011 – char. 196; Gauthier, 1986).

132. Dorsal vertebrae, neural spine distal expansion (“spine-tables”): (0) present; (1) absent (modified from Nesbitt, 2011 – char. 197).

Nesbitt (2011) originally included two different states for the presence of this character, with a separate state for the condition in dinosaurs which was not considered to be homologous to the distal expansion in other archosaurs. The dinosaurian state was eliminated here and the states were simplified to presence or absence. Since many of the outgroup taxa used here possess “spine-tables”, “present” is scored as (0).

133. Dorsal vertebrae, neural spines: (0) about the same height as the posterior cervical neural spines; (1) 2-5 times taller than the posterior cervical neural spines (Nesbitt, 2011 – char. 198).

134. Middle dorsal vertebrae, diapophyses and parapophyses: (0) close to the body of the centrum; (1) expanded on stalks (Nesbitt, 2011 – char. 199).

135. Sacral centra: (0) separate; (1) coossified at the ventral edge (Nesbitt, 2011 – char. 200).

136. Sacral vertebrae, prezygapophyses and complimentary postzygapophyses: (0) separate; (1) coossified (Nesbitt, 2011 – char. 201).

137. Primordial sacral one, sacral rib: (0) does not or weakly articulates with anteriorly directed process (= preacetabular process) of ilium; (1) an anterior process of the rib articulates with the anteriorly directed process of the ilium (Nesbitt, 2011 – char. 202; Nesbitt, 2005, 2007).

138. Sacral vertebrae, centra articular rims: (0) present in sacrum; (1) nearly obliterated (Nesbitt, 2011 – char. 204; Nesbitt, 2007).

139. Dorsal vertebrae: (0) free from sacrum; (1) incorporated into the sacrum, with their ribs/ transverse processes articulating with the pelvis (Nesbitt, 2011 – char. 205; Sereno et al., 1993).

140. Caudal vertebrae: (0) free from sacrum; (1) incorporated into the sacrum, with their ribs/ transverse processes articulating with the pelvis (Nesbitt, 2011 – char. 206; Galton, 1976).

141. Insertion of a sacral vertebra between the first and second primordial sacral vertebrae: (0) absent; (1) present (Nesbitt, 2011 – char. 207).

142. Sacral ribs: (0) almost entirely restricted to a single sacral vertebrae; (1) shared between two sacral vertebrae (Nesbitt, 2011 – char. 208).

143. Middle caudal vertebrae, accessory laminar process on anterior portion of neural arch: (0) absent; (1) present (Nesbitt, 2011 – char. 210; Benton and Clark, 1988).

144. Distal caudal vertebrae, prezygapophyses: (0) not elongate; (1) elongate, more than a quarter of the adjacent centrum length (Nesbitt, 2011 – char. 211; Gauthier, 1986).

**Dermal Skeleton**

145. Osteoderms, dorsal to the vertebral column: (0) present; (1) absent (reversed from Nesbitt, 2011 – char. 401; Gauthier, 1984).

146. Osteoderms, presacral, dorsal, anterior edge: (0) straight or rounded; (1) with distinct anterior process (leaf shaped) (Nesbitt, 2011 – char. 403; Clark et al., 2000 – char. 32).

147. Osteoderms, presacral, paramedian: (0) flat or weakly arched; (1) with distinct longitudinal bend near lateral edge (Nesbitt, 2011 – char. 404; Clark et al., 2000 – char. 33).

148. Appendicular osteoderms: (0) absent; (1) present (Nesbitt, 2011 – char. 405; Heckert and Lucas, 1999).

149. Presacral osteoderms: (0) square (approximately equal length and width); (1) longer than wide; (2) wider than long (Nesbitt, 2011 – char. 407).

150. Osteoderm, anterior bar: (0) absent; (1) present (Nesbitt, 2011 – char. 408; Heckert and Lucas, 1999).

151. Abdominal osteoderms: (0) absent; (1) present (Nesbitt, 2011 – char. 409; Heckert and Lucas, 1999).

152.* Osteoderms on the ventral surface of the tail: (0) absent; (1) present (**new**).

Osteoderms on the ventral surface of the tail have been known for several pseudosuchian taxa (e.g., Walker, 1961; Schoch, 2007; Lautenschlager and Desojo, 2011), but have only recently been found in multiple paracrocodylomorphs, including *Decuriasuchus* (França et al., 2011).

153. Dorsal osteoderms, relation to presacral vertebrae: (0) one to one; (1) more than one row per vertebra (Nesbitt, 2011 – char. 410; modified from Gauthier, 1986).

154. Dorsal osteoderms, midline alignment: (0) mirrored (one to one); (1) staggered (reversed from Nesbitt, 2011 - char. 411).

**Appendicular Skeleton – Shoulder Girdle**

155. Forelimb - hind limb, length ratio: (0) more than 0.55; (1) less than 0.55 (Nesbitt, 2011 – char. 212; Gauthier, 1984).

156.* Clavicles: (0) present and unfused; (1) absent (modified from Nesbitt, 2011 – char. 213; modified from Gauthier, 1986).

This character previously coded for the presence of a furcula (= fused clavicles) and taxa without clavicles were scored as inapplicable (Nesbitt, 2011). Since fused clavicles are only found in theropod dinosaurs and clavicles are absent in crocodylomorphs (Nesbitt, 2011), state (1) was changed from “fused into a furcular” to “absent”.

157. Scapula, length: (0) more than 75% of the humerus length; (1) less than 75% of the humerus length (Nesbitt, 2011 – char. 216; Sereno, 1991).

158. Scapula, entire anterior margin: (0) markedly concave; (1) convex or fairly straight (reversed from Nesbitt, 2011 – char. 217).

159. Scapula, teardrop-shaped tuber on the posterior edge just dorsal to the glenoid fossa: (0) absent; (1) present (Nesbitt, 2011 – char. 219).

160. Scapula, acromion process: (0) in the same plane as the ventral edge of the scapula; (1) distinctly raised above the ventral edge of the scapula (Nesbitt, 2011 – char. 220).

161. Scapulocoracoid, anterior margin (acromion): (0) distinct notch between the two elements; (1) uninterrupted edge between the two elements ("squared off") (Nesbitt, 2011 – char. 221; Parrish, 1993).

162.* Coracoid, postglenoid process: (0) absent; (1) present but exceptionally short; (2) present as clear, rod-shaped process, but <50% total coracoid length; (3) present as elongate, rod-shaped posterior process; (4) present as posteroventrally projecting, blade-shaped process. (modified: new formulation; combined from Nesbitt, 2011 – char. 222 and 223 and Clark et al., 2004 – char. 29).

This character has been expanded in order to capture a greater diversity in the morphology of the coracoids of basal crocodylomorphs. Most pseudosuchians possess a postglenoid process, defined by the presence of a notch ventral to the glenoid (Nesbitt, 2011). Amongst non-crocodyliform crocodylomorphs, this process extends into a rod-shaped, posteriorly projecting process that is variable in length. Length of the process was calculated relative to the total length of the coracoid, postglenoid process included. The length of the process itself was taken from the anterior extent of the postglenoid notch to the posterior termination of the process. In Crocodyliformes, the coracoid begins to mirror the scapula along the parasagittal plain, with the distal extent of the postglenoid process expanding into a blade and the entire process projecting posteroventrally, as opposed to only posteriorly.

163. Coracoid, posteroventral edge, deep groove: (0) absent; (1) present (Nesbitt, 2011 – char. 224).

164. Coracoid, posteroventral portion, biceps tubercle: (0) absent; (1) present (Nesbitt, 2011 – char. 225).

165. Glenoid, orientation: (0) posterolateral; (1) posteroventral (Nesbitt, 2011 – char. 227; Fraser et al., 2002).

166. Coracoid, deep fossa on the posterodorsal edge: (0) absent; (1) present (Nesbitt, 2011 – char. 228).

167. Coracoid, sharp ridge leading from the glenoid to anteroventral corner: (0) absent; (1) present (Nesbitt, 2011 – char. 229).

**Appendicular Skeleton – Forelimb**

168. Humerus, proximal head: (0) confined to the proximal surface; (1) posteriorly expanded and hooked (Nesbitt, 2011 – char. 232).

169. Humerus, proximal articular surface: (0) continuous with the deltopectoral crest; (1) separated by a gap from the deltopectoral crest (Nesbitt, 2011 – char. 233).

170. Humerus, supinator process: (0) present; (1) absent (Nesbitt, 2011 – char. 234; Benton and Clark, 1988).

171.* Humerus, deltopectoral crest: (0) in line with or projecting at only a slight angle (<45°) to the long axis (mediolateral) of the proximal head; (1) thin crest projecting at about 90° to the long axis of the proximal head (**new**).

In many taxa considered here (e.g. *Postosuchus alisonae, Batrachotomus*) the deltopectoral crest is present as a bulbous tuberosity that projects only slightly from the lateral side of the proximal head. In crocodylomorphs, including *Dromicosuchus* (NCSM 13733) and *Hesperosuchus agilis* (AMNH FR 6758), the deltopectoral crest is a thin blade projecting at nearly 90° posteriorly from the proximal head.

172*. Humerus, distal end transverse width: (0) greater than 25% of humerus length; (1) less than 25% of humerus length (**new**).

In most pseudosuchians, such as *Postosuchus alisonae* (NCSM 13731), *Stagonolepis robertsoni* (Walker, 1961), and *Carnufex carolinensis*, the distal head is well expanded compared to the shaft of the humerus, whereas the distal head remains slender, expanding less than 25% compared to the shaft, in crocodylomorphs (e.g. CM 29894; NCSM 13733; AMNH FR 6758) and *Shuvosaurus* (Nesbitt, 2011).

173. Humerus, proximal portion: (0) expanded more than twice the width of the midshaft; (1) expanded less than twice the width of the midshaft (Nesbitt, 2011 – char. 236; Nesbitt, 2007).

174. Ulna, lateral tuber (radius tuber) on the proximal portion: (0) absent; (1) present (Nesbitt, 2011 – char. 237).

175. Ulna, distal end: (0) anteroposteriorly compressed or rounded; (1) with anterior expansion (Nesbitt, 2011 – char. 239).

176*. Radius, proximal end, distinct medial expansion: (0) absent; (1) present (**new**).

The proximal head of the radius in CM 29894 is expanded medially into an L-shape in posterior view. Clark et al. (2000) first identified the expansion in the specimen (CM 29894) they referred to “*Hesperosuchus*” and has now been observed in another basal crocodylomorph that has not yet been described.

177. Radius, distal end: (0) convex; (1) shallow longitudinal groove on the posterior side (Nesbitt, 2011 – char. 240).

178. Radius length: (0) longer than 80% of humerus length; (1) shorter than 80% of humerus length (Nesbitt, 2011 – char. 241; Langer and Benton, 2006).

179. Proximal carpals (radiale, ulnare): (0) equidimensional; (1) elongate (Nesbitt, 2011 – char. 242; Benton and Clark, 1988).

180. Ulnare, length: (0) shorter than the longest metacarpal; (1) longer than the longest metacarpal (Nesbitt, 2011 – char. 243).

181. Metacarpals, proximal ends: (0) overlap; (1) abut one another without overlapping (Nesbitt, 2011 – char. 246; Sereno and Wild, 1992 – char. 14).

182. Distal carpal V: (0) present; (1) absent (Nesbitt, 2011 – char. 249; Sereno, 1999).

**Appendicular Skeleton – Pelvic Girdle**

183. Ilium, supraacetabular crest/rim: (0) projects laterally or ventrolaterally; (1) projects ventrally (Nesbitt, 2011 – char. 264; Gauthier, 1986).

184*. Ilium, crest dorsal to the supraacetabular crest: (0) absent; (1) present, vertical; (2) present, anterodorsally inclined (combined Nesbitt, 2011 – char. 265 and 266).

State 265 of Nesbitt (2011) described both presence/absence of a crest dorsal to the supraacetabular crest and the position of the dorsal crest. When a dorsal crest is present in paracrocodylomorphs, it divides the anterior process of the ilium from the posterior process – state (1) of Nesbitt (2011) – allowing state (2) to be eliminated (a crest confluent with the anterior process, typically found in dinosauromorphs). Among paracrocodylomorphs, there is instead variation in the orientation of this crest, so Nesbitt’s (2011) state 266 was incorperated to produce a character describing presence/absence and the orientation (vertical versus anterodorsally inclined) of the crest instead of its position.

185*. Ilium, crest dorsal to the supraacetabular crest: (0) thick and well defined; (1) broad, round, and poorly defined; (2) thin ridge (modified from Nesbitt, 2011 – char. 267).

The states of this character have been expanded to better describe the variation in morphology seen in the crest dorsal to the supraacetabular crest among paracrocodylomorphs. Among Paracrocodylomorpha, Nesbitt (2011) scored all members of Loricata with a dorsal crest preserved and the poposauroids *Arizonasaurus babbitti* and *Lotosaurus adentus* as having a “thick” crest dorsal to the supraacetabular crest. This crest is typically well defined, like in *Postosuchus* (Weinbaum, 2013). In the basal crocodylomorphs *Dromicosuchus grallator* (NCSM 13733) and *Hesperosuchus agilis* (AMNH FR 6758) the ridge is clearly present, but broad and rounded and these taxa are scored (1). The poposauoids *Poposaurus gracilis*, *Sillosuchus longicervix*, *Effigia okeefeae*, and *Shuvosaurus inexpectatus* are scored (2) for a thin ridge following Nesbitt (2011).

186. Ilium, preacetabular process: (0) short and does not extend anterior to the pubic peduncle; (1) long and extends anterior to the pubic peduncle, but shorter than the postacetabular process; (2) subequal or longer than the postacetabular process. ORDERED (Nesbitt, 2011 – char. 269; modified from Galton, 1976).

187. Ilium, orientation relative to the sagittal plane: (0) roughly vertical (0-20 degrees); (1) ventrolaterally deflected about 45 degrees (Nesbitt, 2011 – char. 270; modified from Benton and Clark, 1988).

188. Ilium, ridge connecting the posterior portion of the supraacetabular crest to the posterior portion of ilium: (0) absent; (1) present (Nesbitt, 2011 – char. 272; modified from Langer and Benton, 2006).

189. Ilium, ventral margin of the acetabulum: (0) convex; (1) concave (**modified** from Nesbitt, 2011 – char. 273).

A “straight” ventral margin of the acetabulum is only found amongst bird-line archosaurs, so Nesbitt’s (2011) character was eliminated here.

190. Ilium, acetabular antitrochanter: (0) absent; (1) present (Nesbitt, 2011 – char. 274; Sereno and Arcucci, 1994).

191. Ilium, dorsal margin dorsal to the supraacetabular rim: (0) rounded or sharp; (1) flat (Nesbitt, 2011 – char. 275).

192. Ilium, region dorsal to the acetabulum: (0) about the same height or shorter than the height of the acetabulum; (1) expanded dorsally, markedly taller than the acetabulum (Nesbitt, 2011 – char. 276).

193. Pubis, length: (0) less than 70% of femoral length; (1) more than 70% of femoral length (Nesbitt, 2011 – char. 278; Novas, 1996).

194*. Pubis, obturator foramen: (0) small; (1) enlarged; (2) absent (modified from Nesbitt, 2011 – char. 281; Sereno and Wild, 1992 – char. 16).

State (2) was added to reflect the absence of an obturator foramen in *Alligator mississippiensis* and *Orthosuchus stormbergi* (Nash, 1975).

195. Pubis, length: (0) shorter or subequal to the ischium; (1) longer than the ischium (Nesbitt, 2011 – char. 282; modified from Benton and Clark, 1988).

196*. Pubis, pubic boot at distal end: (0) absent; (1) present, mediolaterally thick and rounded; (2) present, mediolaterally thin (modified from Nesbitt, 2011 – char. 283 and 284; Gauthier, 1986).

Characters 283 and 284 of Nesbitt (2011) describe the form of the expansion of the distal end of the pubis relative to the shaft, resulting in a pubic boot and are combined here.

197. Pubis, expanded distal margin: (0) shorter than 33% of the length of the shaft of the pubis; (1) greater than 33% of the length of the shaft of the pubis (Nesbitt, 2011 – char. 285; Nesbitt and Norell, 2006).

198. Ischium-pubis, contact: (0) present and extended ventrally; (1) present and reduced to a thin proximal contact; (2) absent. ORDERED (Nesbitt, 2011 – char. 287; modified from Benton and Clark, 1988).

199. Pubis, pubic apron, proximal portion: (0) similar anteroposterior thickness as the rest of the pubic apron; (1) thickened process (Nesbitt, 2011 – char. 288; Nesbitt, 2005, 2007).

200. Pubis, mediolateral width of distal portion: (0) nearly as broad as proximal width; (1) significantly narrower than proximal width; (2) mediolaterally compressed and not broader than anteroposteriorly deep (Nesbitt, 2011 – char. 289; Galton, 1976).

201. Pubis, lateral side of the shaft: (0) smooth; (1) elongate ridge (Nesbitt, 2011 – char. 290).

202. Ischium, medial contact with antimere: (0) restricted to the medial edge; (1) extensive contact but the dorsal margins are separated; (2) extensive contact and the dorsal margins contact each other. ORDERED (Nesbitt, 2011 – char. 291).

203. Ischium, cross-section of the distal portion: (0) platelike; (1) rounded or semicircular (modified from Nesbitt, 2011 – char. 293; modified from Sereno, 1999).

State 2 – a subtriangular cross section of the distal portion of the ilium – is only found among some dinosaurs and was eliminated here.

204. Ischium, distal portion, ischial boot: (0) absent; (1) present (Nesbitt, 2011 – char. 294; Smith and Galton, 1990).

In this context, an ischial boot specifically refers to a “distal bulge” (Langer and Benton, 2006), as opposed to the marked anteroposterior flaring seen in crocodyliforms. The distal expansion seen in taxa scored as 1 here is more club-like, whereas the expansion in crocodyliforms and some other taxa possess a distal expansion that is flattened and paddle-like and therefore not the same as the “ischial boot” initially identified in basal dinosaur (Smith and Galton, 2006; Holtz, 1994; Hutchinson, 2001; Rauhut, 2003; Langer and Benton, 2006). In future analyses, this character will likely need to be modified to accommodate these differing morphologies of the distal portion of the ischium.

205. Ischium, ventral margin: (0) continuous ventral margin; (1) abrupt change in angle between the proximal end and the shaft (modified from Nesbitt, 2011 – char. 296; modified from Sereno et al., 1996).

A notch on the ventral margin of the ischium is a synapomorphy of Neotheropoda (Rauhut, 2003), so there character used by Nesbitt (2011) was not included.

206. Ischium, proximal articular surfaces: (0) articular surfaces with the ilium and the pubis continuous with each other; (1) articular surfaces with ilium and pubis separated by nonarticulating concave surface (modified from Nesbitt, 2011 – char. 297; modified form Irmis et al., 2007).

Nesbitt (2011) included a state for a fossa within the articular surface with the ilium and the pubis. Since such a fossa has not been observed in any paracrocodylomorph taxa included in this analysis, the state was removed.

207. Ischium length: (0) about the same length or shorter than the dorsal margin of the iliac blade; (1) markedly longer than the dorsal margin of the iliac blade (Nesbitt, 2011 – char. 298; Juul, 1994).

**Appendicular Skeleton – Hind Limb**

208. Tibia-femur length: (0) femur longer or about the same length; (1) tibia longer (Nesbitt, 2011 – char. 299; modified from Gauthier, 1986).

209. Femur, proximal portion, anteromedial tuber: (0) absent; (1) small and rounded; (2) large and "hooked" posteriorly (modified from Nesbitt, 2011 – char. 300; modified from Gauthier, 1986).

Nesbitt’s (2011) state (2) – offset medially relative to the posteromedial tuber – was removed as it pertains only to theropod dinosaurs.

210*. Femur, proximal portion, posteromedial tuber: (0) largest of the proximal tubera; (1) small (modified from Nesbitt, 2011 – char. 301; modified from Novas, 1996).

All taxa included in this analysis, with the proximal head of the femur preserved, possess a posteromedial tuber, so the “absent” state was removed. In all outgroup taxa, the posteromedial tuber is the largest of the proximal tubera.

211. Femur, proximal portion, anterolateral tuber: (0) present as an expansion; (1) absent, the anterolateral face is flat (Nesbitt, 2011 – char. 302; modified from Sereon and Arcucci, 1994).

212.* Femur, femoral head orientation: (0) anteromedial (20°-60°); (1) anterior (60°-90°) (modified from Nesbitt, 2011 – char. 305; modified from Benton and Clark, 1988).

The femoral head orientation is taken as the long axis of the femoral head angle with respect to the transverse axis through the femoral condyles (Parrish, 1986). The character for a medially (0°-20°) oriented femoral head pertains only to some theropod dinosaur and was removed. An anteromedially directed (20°-60°) femoral head is found in nearly all taxa considered here, except for *Machaeroprosopus pristinus* and *Orthosuchus stormbergi* which are scored (1) here for an anteriorly (60°-90°) directed femoral head (Nesbitt, 2011).

213. Femur, dorsolateral margin of the proximal portion: (0) smooth; (1) sharp ridge (dorsolateral trochanter) (modified from Nesbitt, 2011 – char. 307).

A rounded ridge is found only among Dinosauria, so Nesbitt’s (2011) state (2) was removed.

214.* Femur, anterior trochanter: (0) absent; (1) present (modified from Nesbitt, 2011 – char. 308; Bakker and Galton, 1974).

This character has typically scored for the presence/absence of the insertion point of the *M. iliofemoralis cranialis* (= anterior trochanter) as well as the form and location of the trochanter. In taxa scored here, only *Riojasuchus* and *Kayentasuchus* possess an anterior trochanter that forms a steep margin with the shaft (Nesbitt, 2011). Therefore, the states for this character have been simplified to only reflect presence/absence.

215. Femur, anterior trochanter shelf proximal to the attachment site for the *M. caudofemoralis*: (0) absent; (1) present (Nesbitt, 2011 – char. 311; modified from Gauthier, 1986).

216. Femur, proximal condylar fold: (0) absent; (1) present (Nesbitt, 2011 – char. 312; Nesbitt et al., 2006).

217. Femur, posterolateral portion of the head: (0) level with the greater trochanter; (1) ventrally descended (Nesbitt, 2011 – char. 313; modified from Novas, 1996).

218. Femur, proximal surface: (0) rounded and smooth; (1) transverse groove (straight) (**modified** from Nesbitt, 2011 – char. 314; Ezcurra, 2006).

A curved transverse groove – Nesbitt (2011) state (2) – is found among basal neotheropods, so the state was removed.

219. Femur, fourth trochanter: (0) present; (1) absent (modified from Nesbitt, 2011 – char. 316; Gauthier, 1986).

Character 316 of Nesbitt (2011) coded for both the shape of the fourth trochanter (= the attachment site for the *M. caudofemoralis*) and its presence or absence. Among crocodile-line archosaurs, nearly all taxa have a moundlike, rounded fourth trochanter, whereas *Shuvosaurus* lacks a fourth trochanter (Nesbitt, 2011), so the character was simplified to reflect presence/absence alone.

220. Femur, angle between the lateral condyle and the crista tibiofibularis in distal view: (0) obtuse; (1) about 90° (Nesbitt, 2011 – char. 319; Parker and Irmis, 2005).

221. Femur, medial condyle of distal portion: (0) tapers to a point on the medial portion in distal view; (1) smoothly rounded in distal view (Nesbitt, 2011 – char. 320).

222. Femur, distal surface between the lateral and medial condyles: (0) flat or nearly flat; (1) groove separating the medial condyle from the lateral condyle (Nesbitt, 2011 – char. 321).

223. Femur, surface between the lateral condyle and crista tibiofibularis on the distal surface: (0) smooth; (1) deep groove (Nesbitt, 2011 – char. 322).

224. Femur, bone wall thickness at or near midshaft: (0) thickness/diameter >0.3; (1) thickness/diameter <0.3 (modified from Nesbitt, 2011 – char. 323).

This character was originally formulated to reflect the degree of “hollowness” of the femur throughout the diversity of basal Archosauria. Since *Poposaurus* and *Terrestrisuchus* are the only taxa with thin-walled femora included here, the states were simplified.

225. Fibula, form of attachment site for the *M. iliofibularis*: (0) crest shaped, low; (1) knob shaped, robust (Nesbitt, 2011 – char. 339; Sereno, 1991).

226. Fibula, location of the attachment site for the *M. iliofibularis*: (0) near the proximal head; (1) near the midpoint between the proximal and distal ends (Nesbitt, 2011 – char. 340; modified from Sereno, 1991).

227. Fibula, proximal end in proximal view: (0) round end in proximal view; (1) mediolaterally compressed (Nesbitt, 2011 – char. 341).

228. Fibula, medial face of the distal portion: (0) smooth; (1) banked with an articular facet that articulates with the astragalus (Nesbitt, 2011 – char. 344).

229. Fibula, distal end in lateral view: (0) angled anterodorsally (asymmetrical); (1) rounded or flat (symmetrical) (Nesbitt, 2011 – char. 345).

230. Distal tarsal IV, posteroventral portion: (0) tapers to point or poorly ventrally expanded; (1) ventrally expanded into a small process (Nesbitt, 2011 – char. 349).

231. Distal tarsal IV, foramen/foramina on medial side: (0) absent; (1) present (Nesbitt, 2011 – char. 352).

232. Distal tarsal IV, proximal surface: (0) flat; (1) distinct, proximally raised region on the posterior portion (Nesbitt, 2011 – char. 353).

233. Astragalus, ridge separating the tibial facet from the posterior edge: (0) thick; (1) thin lamina (Nesbitt, 2011 – char. 354).

234. Articular facet for the astragalus of the calcaneum lies: (0) completely medial to the fibular facet; (1) partially ventral to the fibular facet (Nesbitt, 2011 – char. 358; modified from Parrish, 1993).

235. Astragalus, tibial articular surface: (0) continuous articular surface; (1) nonarticular fossa present on the posterior portion (Nesbitt, 2011 – char. 364).

236. Astragalus, articular surface for the calcaneum: (0) stretches from fibular facet of the astragalus to the ventral margin; (1) restricted to the ventral surface of the fibular facet, clearly separated from the ventral margin (Nesbitt, 2011 – char. 367).

237. Calcaneum, ventral articular surface for the distal tarsal IV and the distal end of the tuber: (0) continuous; (1) separated by a clear gap; (2) separated by a gap with a ventral fossa. ORDERED (Nesbitt, 2011 – char. 371).

238. Calcaneum, articular facets for the fibula and astragalus: (0) connected by a continuous surface; (1) separated (Nesbitt, 2011 – char. 372).

239. Calcaneum, calcaneal tuber, dorsomedially aligned median depression on distal face: (0) absent; (1) present (Nesbitt, 2011 – char. 375; Parrish, 1993).

240. Calcaneum, calcaneal tuber, shaft proportions at the midshaft of the tuber: (0) about the same width or only slightly wider than the fibular facet; (1) nearly twice as wide as the fibular facet (modified from Nesbitt, 2011 – char. 376; modified from Sereno, 1991).

Many taxa considered here have relatively thin clacaneal tubera, such as *Postosuchus* *alisonae* (NCSM 13731), CM 73372, and “*Hesperosuchus*” (YPM 41198) whereas many basal members of Suchian, including basal paracrocodylomorphs, possess a relatively thick (nearly twice as wide as the fibular facet) shaft of the calcaneal tuber (Nesbitt, 2011). The two states listed here were simplified from the three states used by Nesbitt (2011) because the third state described a particularly tall tuber shaft found only in non-archosaurian archosauromorphs.

241. Calcaneum, articular surface for the fibula: (0) convex; (1) hemicylindrical (modified from Nesbitt, 2011 – char. 378; modified from Sereno, 1991).

Taxa considered here possess only convex fibular facets of the calcaneum and not the concave condition found in some bird-line archosaur (Sereno, 1991). Nesbitt (2011) distinguishes between the simpler convex facet and the hemicylindrical convex facet based on a morphology in which the articulation is convex anteroposteriorly and mediolaterally, mediolaterally constricted, and bordered medially by a nonarticular surface.

242. Metatarsus, configuration: (0) metatarsals diverging from ankle; (1) compact metatarsus, with metatarsals II-IV tightly bunched for half of the length (Nesbitt, 2011 – char. 382; Gauthier, 1986).

243. Metatarsal I, length, relative to the length of metatarsal III: (0) 0%-84%; (1) 85% or more (Nesbitt, 2011 – char. 387; Sereno, 1991).

244. Metatarsal III: (0) longer than metatarsal II; (1) subequal to metatarsal II (Nesbitt, 2011 – char. 390).

245. Metatarsal IV, distal articulation surface: (0) broader than deep (nearly symmetrical); (1) as broad as deep or deeper than broad (asymmetrical) (Nesbitt, 2011 – char. 391; modified from Sereno, 1991).

246. Metatarsal IV length: (0) longer than metatarsal II; (1) subequal to metatarsal II (Nesbitt, 2011 – char. 395; modified from Gauthier, 1986).

247. Pedal digit IV, number of phalanges: (0) five; (1) four or fewer (Nesbitt, 2011 – char. 396; Parrish, 1993).

248. Metatarsal V, dorsal prominence separated from the proximal surface by a concave gap: (0) absent; (1) present (Nesbitt, 2011 – char. 397).

249. Metatarsal V, "hooked" proximal end: (0) present; (1) absent, with articular face for distal tarsal 4 subparallel to shaft axis (Nesbitt, 2011 – char. 398; Sereno, 1991).

250. Metatarsal V, phalanges: (0) present and "fully" developed first phalanx; (1) present and "poorly" developed first phalanx; (0) without phalanges and metatarsal tapers to a point. ORDERED (Nesbitt, 2011 – char. 399; modified from Gauthier, 1984).

251. Pedal unguals: (0) weekly mediolaterally compressed; (1) dorsolaterally compressed; (2) strongly mediolaterally compressed, with sharp dorsal keel (Nesbitt, 2011 – char. 400; modified from Sereno, 1991).

**References**

Alcober O. Redescription of the skull of *Saurosuchus galilee* (Archosauria: Rauisuchidae). Journal of Vertebrate Paleontology. 2000; 20: 302–316.

Archie JW. Methods for coding variable morphological features for numerical taxonomic analysis. Systematic Zoology. 1985; 34: 326–345.

Bakker RT, Galton PM. Dinosaur monophyly and a new class of vertebrates. Nature. 1974; 248: 168–172.

Benton MJ. *Scleromochlus taylori* and the origin of dinosaurs and pterosaurs. Philosophical Transactions of the Royal Society of London Series B Biological Sciences. 1999; 354: 1423–1446.

Benton MJ, Clark JM. Archosaur phylogeny and the relationships of the Crocodylia. In: Benton MJ, editor. The Phylogeny and Classification of the Tetrapods, 1: Amphibians, Reptiles, Birds. Oxford: Clarenden Press; 1988. pp. 295–338

Benton MJ, Walker AD. *Erpetosuchus*, a crocodile-like basal archosaur from the Late Triassic of Elgin, Scotland. Biological Journal of the Linnean Society. 2002; 136: 25–47.

Bonaparte JF. Dos nuevas ‘‘faunas’’ de reptiles Triasicos de Argentina. Gondwana Stratigraphy. IUGS Symposium. 1967; 283–306.

Bonaparte JF. Classification of the Thecodontia. Géobios, Mémoire Spécial. 1982; 6: 99–112.

Brusatte SL, Benton MJ, Ruta M, Lloyd FT. The first 50 Myr of dinosaur evolution: macroevolutionary pattern and morphological disparity. Biology Letters. 2008; 4(6): 733-736.

Brusatte SL, Benton MJ, Desojo JB, Langer MC. The higher-level phylogeny of Archosauria (Tetrapoda: Diapsida). Journal of Systematic Palaeontology. 2010; 8: 3–47.

Butler RJ. The ‘fabrosaurid’ ornithischian dinosaurs of the upper Elliot Formation (Lower Jurassic) of South Africa and Lesotho. Zoological Journal of the Linnean Society. 2005; 145: 175–218.

Butler RJ, Sullivan C, Ezcurra MD, Liu J, Lecuona A, Sookias RB. New clade of enigmatic early archosaurs yields insights into early pseudosuchian phylogeny and biogeography of the archosaur radiation. BMC Evolutionary Biology. 2014. doi:10.1186/1471-2148-14-128

Clark JM. Patterns of evolution in Mesozoic Crocodyliformes. In: Fraser NC, Sues HD, editors. In the Shadow of the Dinosaurs: Early Mesozoic Tetrapods. New York: Cambridge University Press; 1994. pp. 84-97

Clark JM, Sues HD. Two new basal crocodylomorph archosaurs from the Lower Jurassic and the monophyly of the Sphenosuchia. Zoological Journal of the Linnean Society. 2002; 136: 77–95.

Clark JM, Sues HD, Berman DS. A new specimen of *Hesperosuchus agilis* from the Upper Triassic of New Mexico and the interrelationships of basal crocodylomorph archosaurs. Journal of Vertebrate Paleontology. 2000; 20: 683–704.

Clark JM, Xing X, Forster CA, Wang Y. A Middle Jurassic ‘sphenosuchian’ from China and the origin of the crocodylian skull. Nature. 2004; 430: 1021–1024.

Ezcurra MD. A review of the systematic position of the dinosauriform archosaur *Eucoelophysis baldwini* Sullivan and Lucas, 1999 from the Upper Triassic of New Mexico, USA. Geodiversitas. 2006; 28: 649–684.

França MAG, Ferigolo J, Langer MC. Associated skeletons of a new middle Triassic ‘Rauisuchian’ from Brazil. Naturwissenschaften. 2011; 98: 389–395.

França MAG, Langer MC, Ferigolo J. The skull anatomy of *Decuriasuchus quartacolonia* (Pseudosuchia: Suchia: Loricata) from the middle Triassic of Brazil. In: Nesbitt SJ, Desojo JB, Irmis RB, editors. Anatomy, Phylogeny and Palaeobiology of Early Archosaurs and their Kin. Geological Society of London Special Publication 379; 2013. pp. 469-501

Fraser NC, Padian K, Walkden GM, Davis ALM. Basal dinosauriform remains from Britain and the diagnosis of the Dinosauria. Palaeontology. 2002; 45: 79–95.

Galton PM. Prosauropod dinosaurs (Reptilia: Saurischia) of North America. Postilla. 1976; 169: 1–98.

Gauthier JA. A cladistic analysis of the higher systematic categories of Diapsida. Ph.D. Dissertation, University of California Berkeley. 1984.

Gauthier JA. Saurischian monophyly and the origin of birds. Memoirs of the California Academy of Science. 1986; 8: 1–55.

Gower DJ. The cranial and mandibular osteology of a new rauisuchian archosaurian from the Middle Triassic of southern Germany. Stuttgarter Beiträge zur Naturkunde B. 1999; 280: 1–49.

Gower DJ. Rauisuchian archosaurs (Reptilia, Diapsida): an overview. Neues Jahrbuch für Geologie und Paläontologie Abhandlungen. 2000; 218:447–488.

Gower DJ. Braincase evolution in suchian archosaurs (Reptilia: Diapsida): evidence from the rauisuchian *Batrachotomus kupferzellensis*. Zoological Journal of the Linnean Society. 2002; 136: 49–76.

Gower DJ, Sennikov AG. Braincase morphology in early archosaurian reptiles. Palaeontology. 1996; 39: 883–906.

Heckert AB, Lucas SG. A new aetosaur (Reptilia: Archosauria) from the upper Triassic of Texas and the phylogeny of aetosaurs. Journal of Vertebrate Paleontology. 1999; 19: 50–68.

Holtz TR. The phylogenetic position of the Tyrannosauridae: implications for theropod systematics. Journal of Paleontology. 1994; 68: 1100–1117.

Hutchinson JR. The evolution of femoral osteology and soft tissue on the line to extant birds (Neornithes). Zoological Journal of the Linnean Society, 2001; 131: 169-197.

Iordansky NN. The skull of the Crocodilia. In: Gans C, Parsons TS, editors. Biology of the Reptilia. Volume 4. Morphology D. New York: Academic Press; 1973. pp. 201-262.

Irmis RB, Parker WG, Nesbitt SJ, Liu J. Ornithischian dinosaurs: the Triassic record. Historical Biology. 2007; 18: 3–22.

Juul L. The phylogeny of basal archosaurs. Palaeontologia Africana. 1994; 31: 1–38.

Langer MC, Benton MJ. Early dinosaurs: a phylogenetic study. Journal of Systematic Palaeontology. 2006; 4: 309–358.

Lautenschlager S, Desojo JB. Reassessment of the Middle Triassic ‘rauisuchian’ archosaurs *Ticinosuchus ferox* and *Stagonosuchus nyassicus*. Paläontologische Zeitschrift. 2011; 85: 357–381.

Li C, Wu XC, Cheng YN, Sato T, Wang L. An unusual archosaurian from the marine Triassic of China. Naturwissenschaften. 2006; 93: 200–206.

Modesto SP, Sues HD. The skull of the Early Triassic archosauromorph *Prolacerta* *broomi* and its phylogenetic significance. Zoological Journal of the Linnean Society. 2004; 140: 335–351.

Nash D. The morphology and relationships of a crocodilian, *Orthosuchus* *strombergi*, from the Upper Triassic of Lesotho. Annals of the South African Museum. 1975; 67: 227–329.

Nesbitt SJ. The anatomy of *Effigia okeeffeae* (Archosauria, Suchia), theropod convergence, and the distribution of related taxa. Bulletin of the American Museum of Natural History. 2007; 302: 1–84.

Nesbitt SJ. The early evolution of archosaurs: relationships and the origin of major clades. Bulletin of the American Museum of Natural History. 2011; 352: 1–292.

Nesbitt SJ, Norell MA. Extreme convergence in the body plans of an early suchian (Archosauria) and ornithomimid dinosaurs (Theropoda). Proceedings of the Royal Society of London B Biological Sciences. 2006; 273: 1045–1048.

Nesbitt SJ, Irmis RB, Lucas SG, Hunt AP. A giant crocodylomorph from the upper Triassic of New Mexico. Paläontolgische Zeitschrift. 2005; 79: 471–478.

Nesbitt SJ, Turner AH, Erickson GM, Norell MA. Prey choice and cannibalistic behavior in the theropod *Coelophysis*. Biology Letters. 2006; 2: 611–614.

Nesbitt SJ, Stocker MR, Small B, Downs A. The osteology and relationships of *Vancleavea* *campi* (Reptilia: Archosauriformes). Zoological Journal of the Linnean Society. 2009; 157: 814–864. (2009)

Nesbitt SJ, Brusatte SL, Desojo JB, Liparini A, França MA, Weinbaum JC, et al. Rauisuchia. In: Nesbitt SJ, Desojo JB, Irmis RB, editors. Anatomy, Phylogeny and Palaeobiology of Early Archosaurs and their Kin. Geological Society of London Special Publication 379; 2013. pp. 241-274.

Novas FE. Dinosaur monophyly. Journal of Vertebrate Paleontology. 1996; 16: 723–741.

Olsen PE, Sues HD, Norell MA. First record of *Erpetosuchus* (Reptilia: Archosauria) from the Late Triassic of North America. Journal of Vertebrate Paleontology. 2000; 20: 633–636.

Parker WG, Irmis RB. Advances in Late Triassic vertebrate paleontology based on new material from Petrified Forest National Park, Arizona. In: Heckert AB, Lucas SG, editors. Vertebrate paleontology in Arizona. Albuquerque: New Mexico Museum of Natural History and Science; 2005. pp. 45-58.

Parrish JM. Locomotor adaptations in the hindlimb and pelvis of the Thecodontia. Hunteria. 1986; 1: 1–35.

Parrish JM. A new specimen of an early crocodylomorph (cf. *Sphenosuchus* sp.) from the Upper Triassic Chinle Formation of Petrified Forest National Park, Arizona. Journal of Vertebrate Paleontology. 1991; 11: 198–212.

Parrish JM. Phylogeny of the Crocodylotarsi, with reference to archosaurian and crurotarsan monophyly. Journal of Vertebrate Paleontology. 1993; 13: 287–308.

Parrish JM. Cranial osteology of *Longosuchus meadei* and the phylogeny and distribution of the Aetosauria. Journal of Vertebrate Paleontology. 1994; 14: 196–209.

Pol D, Rauhut OW, Lecuona A, Leardi JM, Xu X, Clark JM. A new fossil from the Jurassic of Patagonia reveals the early basicranial evolution and the origins of Crocodyliformes. Biological Reviews. 2013; 88(4): 862-872.

Rauhut OWM. The interrelationships and evolution of basal theropod dinosaurs. Special Papers in Palaeontology. 2003; 69: 1–214.

Schoch RR. Osteology of the small archosaur Aetosaurus from the Upper Triassic of Germany. Neues Jahrbuch für Geologie und Paläontologie, Abhandlungen. 2007; 246: 1–35.

Sereno PC. Phylogeny of the bird-hipped dinosaurs (Order Ornithischia). National Geographic Society Research. 1986; 2: 234–256.

Sereno PC. Basal archosaurs: phylogenetic relationships and functional implications. Journal of Vertebrate Paleontology. 1991; 10(3): 1–53.

Sereno PC, Arcucci AB. Dinosaurian precursors from the Middle Triassic of Argentina: *Marasuchus lilloensis*, gen. nov. Journal of Vertebrate Paleontology. 1994; 14: 53–73.

Sereno PC, Novas FE. The complete skull and skeleton of an early dinosaur. Science. 1992; 258: 1137–1140.

Sereno PC, Wild R. *Procompsognathus*: theropod, ‘‘thecodont’’ or both? Journal of Vertebrate Paleontology. 1992; 12: 435–458.

Sereno PC, Forester CA, Rogers RR, Monetta AM. Primitive dinosaur skeleton from Argentina and the early evolution of Dinosauria. Nature. 1993; 361: 64–66.

Sereno PC, Wilson JA, Larsson HCE, Dutheil, DB, Sues HD. Early cretaceous dinosaurs from the Sahara. Science. 1994; 266: 267–270.

Sereno PC, Dutheil DB, Iarochene M, Larsson CE, Lyon GH, Magwene PM, et al. Predatory dinosaurs from the Sahara and Late Cretaceous faunal differentiation. Science. 1996; 272: 986–991.

Smith DK, Galton PM. Osteology of *Archaeornithomimus asiaticus* (Upper Cretaceous, Iren Dabasu Formation, People’s Republic of China. Journal of Vertebrate Paleontology. 1990; 10: 255–265.

Sues HD, Olsen PE, Carter JG, Scott DM. A new crocodylomorph archosaur from the Upper Triassic of North Carolina. Journal of Vertebrate Paleontology. 2003; 23: 329–343.

Sulej T. A new rauisuchian reptile (Diapsida: Archosauria) from the Late Triassic of Poland. Journal of Vertebrate Paleontology. 2005; 25: 78–86.

Thorpe RS. Coding morphometric characters for constructing Distance Wagner networks. Evolution. 1984; 38: 244-255.

Walker AD. Triassic reptiles from the Elgin area: *Stagonoleplis*, *Dasygnathus*, and their allies. Philosophical Transactions of the Royal Society of London. 1961; 244: 103–204.

Walker AD. A revision of *Sphenosuchus acutus* Haugton, a crocodylomorph reptile from the Elliot Formation (Late Triassic or Early Jurassic) of South Africa. Philosophical Transactions of the Royal Society of London, Series B. 1990; 330: 1–120

Weinbaum JC. The skull of *Postosuchus kirkpatricki* (Archosauria: Paracrocodyliformes) from the Upper Triassic of the United States. PaleoBios. 2011; 30: 18–44.

Weinbaum JC. Postcranial skeleton of *Postosuchus kirkpatricki* (Archosauria: Paracrocodylomorpha), from the upper Triassic of the United States. In: Nesbitt SJ, Desojo JB, Irmis RB, editors. Anatomy, Phylogeny and Palaeobiology of Early Archosaurs and their Kin. Geological Society of London Special Publication 379; 2013. pp. 525–553.

Weinbaum JC, Hungerbühler A. A revision of *Poposaurus gracilis* (Archosauria: Suchia) based on two new specimens from the Late Triassic of the southwestern U.S.A. Paläontologische Zeitschrift. 2007; 81/82: 131–145.

Witmer LM. The evolution of the antorbital cavity of archosaurs: a study in soft-tissue reconstruction in the fossil record. Journal of Vertebrate Paleontology Memoir. 1997; 3: 1–73.

Wu XC, Chatterjee S (1993) *Dibothrosuchus elaphros*, a crocodylomorph from the Lower Jurassic of China and the phylogeny of the Sphenosuchia. Journal of Vertebrate Paleontology 13: 58–89.

Yates AM. The species taxonomy of the sauropodomorph dinosaurs from the Lowenstein Formation (Norian, Late Triassic) of Germany. Palaeontology. 2003; 46: 317–337.
