# Supplementary material for: Osteology of Carnufex carolinensis (Archosauria: Psuedosuchia) from the Pekin Formation of North Carolina and Its Implications for Early Crocodylomorph Evolution
Source: PLoS One. 2016 Jun 15;11(6):e0157528. doi: 10.1371/journal.pone.0157528 (PMC4909254; doi:10.1371/journal.pone.0157528)
Supplement: S3 File — (DOCX) [file pone.0157528.s003.docx]

**S3 File. Phylogenetic data matrix.**

|  | 1 | 2 | 3 | 4 | 5 | 6 | 7 | 8 | 9 | 10 | 11 | 12 | 13 | 14 | 15 |
| --- | --- | --- | --- | --- | --- | --- | --- | --- | --- | --- | --- | --- | --- | --- | --- |
| *Euparkeri capensis* | 0 | 1 | 0 | 0 | 0 | 0 | 0 | 0 | 0 | 0 | 0 | 0 | 0 | 0 | 0 |
| *Machaeroprosopus pristinus* | ? | ? | 0 | 0 | 4 | 0 | 0 | 1 | 1 | 0 | 0 | 0 | 0 | 0 | 0 |
| *Riojasuchus tenulsceps* | 0 | 0 | 0 | 0 | 0 | 0 | 0 | 0 | 0 | 0 | 0 | 0 | 0 | 0 | 0 |
| *Revueltosaurus callenderi* | 0 | 0 | 0 | 0 | 2 | 0 | 0 | 0 | 1 | 0 | 0 | 0 | 0 | 0 | 0 |
| *Stagonolepis robertsoni* | 0 | 0 | 0 | 0 | 2 | 0 | 0 | 0 | 1 | 0 | 0 | 0 | 0 | 0 | 1 |
| *Turfanosuchus dabanensis* | 0 | 0 | 0 | 0 | 2 | 0 | 0 | 0 | ? | 0 | 0 | 0 | 0 | ? | 0 |
| *Gracilisuchus stipanicicorum* | 0 | 1 | 0 | 0 | 1 | 0 | 0 | 0 | 0/1 | 0 | 0 | 0 | 0 | ? | 0 |
| *Ticinosuchus ferox* | ? | ? | ? | ? | ? | ? | ? | 0 | 0 | ? | 0 | ? | 0 | 0 | ? |
| *Qianosuchus mixtus* | 1 | 0 | 1 | 0 | 4 | 0 | 0 | 1 | 0 | 0 | 0 | 2 | 0 | 0 | 1 |
| *Arizonasaurus babbitti* | ? | ? | 1 | ? | ? | 0 | 0 | 0 | 0 | 0 | 0 | 0 | 0 | 0 | 1 |
| *Xilousuchus sapingensis* | ? | 0 | 1 | 0 | 2 | 0 | 0 | 0 | 0 | 0 | 0 | 0 | 0 | 0 | 1 |
| *Poposaurus gracilis* | ? | ? | 1 | ? | ? | ? | ? | 0 | ? | 0 | 0 | 0 | ? | 0 | ? |
| *Lotosaurus adentus* | ? | 0 | ? | 1 | - | 0 | 0 | 0 | ? | ? | 1 | ? | 1 | ? | 0 |
| *Sillosuchus longicervix* | ? | ? | ? | ? | ? | ? | ? | ? | ? | ? | ? | ? | ? | ? | ? |
| *Shuvosaurus inexpectatus* | 1 | 0 | 1 | 1 | - | 0 | ? | ? | ? | ? | 1 | ? | ? | ? | ? |
| *Effigia okeeffeae* | 1 | 0 | 1 | 1 | - | 0 | ? | 0 | ? | ? | 1 | ? | 1 | ? | 1 |
| *Prestosuchus* | 0 | 1 | ? | 0 | 1 | 0 | 0 | 0 | 0 | ? | 0 | ? | 0 | ? | 0 |
| *Saurosuchus galilei* | 0 | 1 | 0 | 0 | 1 | 0 | ? | - | 0 | 0 | 0 | 0 | 0 | 0 | 0 |
| *Batrachotomus kuperferzellensis* | ? | ? | 0 | 0 | 1 | 1 | 0 | 0 | 0 | 0 | 0 | 1 | 0 | 0 | 1 |
| *Decuriasuchus quartacolonia* | ? | 1 | 0 | 0 | 1 | 1 | 0 | 0 | 0 | 0 | 0 | 0 | 0 | 0 | ? |
| *Fasolasuchus tenax* | 0 | 1 | 0 | 0 | 1 | 0 | 0 | 0 | ? | 0 | 0 | ? | 0 | 1 | 0 |
| *Rauisuchus triradentes* | 0 | 1 | ? | 0 | 1 | 0 | 0 | 0 | ? | ? | ? | ? | ? | ? | ? |
| *Postosuchus alisonae* | ? | ? | ? | ? | ? | ? | ? | ? | ? | ? | ? | ? | ? | ? | ? |
| *Postosuchus kirkpatricki* | 0 | 1 | 0 | 0 | 1 | 0 | 0 | 0 | 1 | 0 | 0 | 1 | 0 | 1 | 0 |
| *Polonosuchus silesiacus* | 0 | 1 | 0 | 0 | 1 | 0 | 0 | 0 | ? | 0 | 0 | 0 | 0 | 1 | 0 |
| *Carnufex carolinensis* | ? | 0 | 2 | 0 | 3 | 0 | 1 | ? | 1 | 0 | 0 | ? | 0 | 0 | 0 |
| CM 73372 | ? | ? | ? | ? | ? | ? | ? | ? | ? | ? | ? | ? | ? | ? | ? |
| *Redondavenator quayensis* | 0 | 1 | 2 | 0 | 2 | 0 | 1 | 1 | ? | ? | 0 | 2 | ? | 0 | 0 |
| *“Hesperosuchus agilis”* | 0 | 0 | 2 | 0 | 2 | 0 | 0/1 | 1 | 1 | 0 | 0 | 2 | 0 | 0 | 0 |
| *Dromicosuchus grallator* | 0 | 0 | 2 | 0 | 2 | 0 | 0/1 | 1 | 1 | 0 | 0 | 2 | 0 | 0 | 0 |
| *Hesperosuchus agilis* HT | ? | ? | ? | ? | ? | ? | ? | ? | 1 | 0 | 0 | 2 | 0 | 0 | ? |
| *Sphenosuchus acutus* | ? | 1 | 2 | 0 | 2 | 0 | 1 | 1 | 1 | 0 | 0 | 2 | 0 | 0 | 0 |
| *Terrestrisuchus gracilis* | ? | ? | ? | ? | ? | ? | ? | 1 | 1 | 0 | 0 | 2 | 0 | 0 | ? |
| *Dibothrosuchus elaphros* | ? | 0 | 2 | 0 | 2 | 0 | 1 | 1 | 1 | 0 | 0 | 2 | 0 | 0 | 0 |
| *Litargosuchus leptorhynchus* | 0 | 0 | 2 | 0 | 1 | 0 | 1 | 1 | 1 | 0 | 0 | ? | 0 | ? | 0 |
| *Kayentasuchus walker* | ? | 1 | 2 | 0 | 1 | 0 | 1 | 1 | 1 | 0 | 0 | ? | 0 | ? | 0 |
| *Junggarsuchus sloani* | 0 | 0 | 2 | 0 | ? | 0 | 0 | 1 | 1 | 0 | 0 | ? | 0 | ? | 0 |
| *Orthosuchus stormbergi* | 0 | 0 | 3 | 0 | 1 | 0 | 1 | 1 | ? | 1 | 0 | 0 | 0 | 0 | 0 |
| *Protosuchus richardsoni* | 0 | 0 | 3 | 0 | 1 | 0 | 1 | 0 | 1 | 0 | 0 | 0 | 0 | ? | 0 |
| *Protosuchus haughtoni* | 0 | 0 | 3 | 0 | 1 | 0 | 1 | 0 | 1 | 0 | 0 | ? | 0 | ? | 0 |
| *Alligator mississippiensis* | 0 | 0 | 3 | 0 | 2 | 0 | 0 | ? | 1 | 0 | 0 | 0 | 0 | 1 | 0 |
|  |  |  |  |  |  |  |  |  |  |  |  |  |  |  |  |
|  | 16 | 17 | 18 | 19 | 20 | 21 | 22 | 23 | 24 | 25 | 26 | 27 | 28 | 29 | 30 |
| *Euparkeri capensis* | 0 | 0 | 0 | 0 | 0 | 1 | 0 | 0 | 0 | 0 | 0 | 2 | 0 | 0 | 0 |
| *Machaeroprosopus pristinus* | 0 | 0 | 0 | 0 | 0 | 0 | 0 | 0 | 0 | 0 | ? | 0 | 0 | 0 | 0 |
| *Riojasuchus tenulsceps* | 0 | 0 | 1 | 1 | 1 | 0 | 1 | 0 | 0 | 0 | 1 | 0 | 0 | 0 | 0 |
| *Revueltosaurus callenderi* | 0 | 1 | 0 | 1 | 0 | 0 | 1 | 0 | 0 | 0 | 0 | 0 | 0 | 0 | 0 |
| *Stagonolepis robertsoni* | 1 | 1 | 0 | 1 | 0 | 0 | 1 | 0 | 0 | ? | 0 | 0 | 1 | 0 | 0 |
| *Turfanosuchus dabanensis* | 0 | 0 | 1 | 0 | 0 | 0 | ? | 0 | 0 | 1 | 0 | 2 | 0 | ? | 1 |
| *Gracilisuchus stipanicicorum* | 0 | 0 | 2 | 0 | 0 | 0 | 1 | 0 | 0 | 1 | 0 | 2 | 0 | 0 | 0 |
| *Ticinosuchus ferox* | 0 | 0 | 1 | 0 | 1 | 0 | ? | ? | ? | ? | 0 | ? | 0 | ? | ? |
| *Qianosuchus mixtus* | 1 | 0 | 2 | 0 | 0 | 0 | 1 | 0 | 0 | 0 | 0 | 2 | 0 | ? | 0 |
| *Arizonasaurus babbitti* | 1 | 0 | 1 | 0 | 0 | 0 | 1 | 0 | 0 | ? | 0 | ? | ? | ? | 0 |
| *Xilousuchus sapingensis* | 1 | 0 | 1 | 0 | 0 | 0 | 1 | ? | 0 | ? | 0 | 1 | ? | ? | ? |
| *Poposaurus gracilis* | 1 | ? | ? | ? | 1 | ? | 1 | ? | ? | ? | ? | ? | ? | ? | ? |
| *Lotosaurus adentus* | 1 | 1 | 1 | 0 | 0 | 1 | 0 | 0 | 0 | 0 | ? | 1 | 0 | 0 | 0 |
| *Sillosuchus longicervix* | ? | ? | ? | ? | ? | ? | ? | ? | ? | ? | ? | ? | ? | ? | ? |
| *Shuvosaurus inexpectatus* | ? | ? | ? | ? | ? | ? | ? | ? | 0 | ? | ? | 1 | 0 | ? | 0 |
| *Effigia okeeffeae* | 1 | 1 | 1 | 0 | 0 | 0 | 0 | 0 | 0 | 0 | 0 | 1 | 0 | 0 | 0 |
| *Prestosuchus* | 0 | 0 | 2 | 1 | 1 | 0 | 1 | 0 | 0 | 0 | ? | 2 | 0 | ? | 0 |
| *Saurosuchus galilei* | 0 | 0 | 2 | 1 | 1 | 0 | 1 | 0 | 0 | 0 | 0 | 2 | 0 | 0 | 1 |
| *Batrachotomus kuperferzellensis* | 1 | 0 | 1 | 0 | 1 | 0 | 1 | 1 | 1 | 1 | 0 | 2 | ? | 0 | 1 |
| *Decuriasuchus quartacolonia* | 0 | 0 | ? | ? | 1 | ? | ? | ? | 0 | 0 | 0 | 2 | ? | ? | 0 |
| *Fasolasuchus tenax* | 0 | 0 | 2 | 0 | 1 | 0 | 1 | 1 | 0 | 1 | ? | ? | ? | ? | ? |
| *Rauisuchus triradentes* | ? | ? | ? | ? | ? | ? | ? | 1 | 1 | ? | 1 | ? | ? | 0 | ? |
| *Postosuchus alisonae* | ? | ? | ? | ? | ? | ? | ? | ? | 1 | ? | 1 | ? | ? | ? | ? |
| *Postosuchus kirkpatricki* | 0 | 2 | 2 | 1 | 1 | 0 | 1 | 1 | 1 | 1 | 1 | 0 | ? | 0 | 1 |
| *Polonosuchus silesiacus* | 0 | 2 | 2 | 1 | 1 | 0 | 1 | 1 | 1 | 1 | 0 | 0 | ? | ? | ? |
| *Carnufex carolinensis* | 0 | 0 | 2 | 1 | 1 | ? | ? | ? | ? | ? | ? | 2 | ? | ? | ? |
| CM 73372 | ? | ? | ? | ? | ? | ? | ? | ? | ? | ? | ? | ? | ? | ? | ? |
| *Redondavenator quayensis* | 0 | 0 | ? | ? | ? | 0 | 2 | ? | 0 | ? | 1 | ? | ? | ? | ? |
| *“Hesperosuchus agilis”* | 0 | 0 | 2 | 0 | 1 | 0 | 2 | 1 | 0 | 0 | 1 | 2 | 1 | ? | 1 |
| *Dromicosuchus grallator* | 0 | 0 | 2 | 0 | 1 | 0 | ? | 1 | 0 | 0 | 1 | 2 | 1 | ? | 1 |
| *Hesperosuchus agilis* HT | ? | 0 | ? | ? | ? | 0 | ? | ? | 0 | ? | ? | ? | ? | ? | 1 |
| *Sphenosuchus acutus* | 0 | 0 | 2 | ? | ? | 0 | 2 | 1 | 0 | ? | 1 | 2 | 1 | 0 | 1 |
| *Terrestrisuchus gracilis* | 0 | 0 | 1 | 0 | 0 | 0 | 2 | ? | ? | 0 | 1 | 2 | ? | ? | ? |
| *Dibothrosuchus elaphros* | ? | 0 | 1 | 0 | 0 | 0 | 3 | 1 | 0 | 0 | 1 | 2 | 1 | 1 | 1 |
| *Litargosuchus leptorhynchus* | ? | 0 | 1 | 0 | 1 | 0 | ? | 0 | 0 | 0 | 1 | 2 | ? | ? | 0 |
| *Kayentasuchus walker* | 0 | 0 | 2 | 0 | 1 | 0 | ? | ? | 0 | ? | 1 | 2 | ? | ? | ? |
| *Junggarsuchus sloani* | 0 | 0 | 2 | ? | 1 | 0 | 2 | 0 | 0 | 0 | 1 | 2 | ? | ? | 1 |
| *Orthosuchus stormbergi* | 0 | 0 | 2 | ? | 1 | 0 | 2 | ? | 0 | ? | ? | 2 | 1 | ? | 0 |
| *Protosuchus richardsoni* | 0 | 0 | 2 | 0 | 1 | 0 | 2 | 0 | 0 | 0 | 1 | 2 | 1 | 1 | 0 |
| *Protosuchus haughtoni* | 0 | 0 | 2 | 0 | 1 | 0 | ? | 0 | 0 | 0 | 1 | 2 | 1 | ? | 0 |
| *Alligator mississippiensis* | 0 | 0 | ? | ? | ? | 0 | 3 | 0 | 0 | ? | 1 | 0 | 1 | 1 | 0 |
|  |  |  |  |  |  |  |  |  |  |  |  |  |  |  |  |
|  |  |  |  |  |  |  |  |  |  |  |  |  |  |  |  |
|  |  |  |  |  |  |  |  |  |  |  |  |  |  |  |  |
|  | 31 | 32 | 33 | 34 | 35 | 36 | 37 | 38 | 39 | 40 | 41 | 42 | 43 | 44 | 45 |
| *Euparkeri capensis* | 0 | 0 | 0 | 0 | 0 | 0 | 0 | 0 | 0 | 0 | 0 | 0 | 0 | 0 | 0 |
| *Machaeroprosopus pristinus* | 0 | 0 | 0 | 0 | 0 | 0 | 0 | 0 | 0 | 0 | 0 | 0 | 0 | ? | 0 |
| *Riojasuchus tenulsceps* | 0 | 0 | 0 | 0 | 0 | 0 | 0 | 0 | 0 | 0 | 0 | 0 | 0 | 0 | 0 |
| *Revueltosaurus callenderi* | 0 | 1 | 1 | 0 | 0 | 0 | 0 | 0 | 0 | 0 | 0 | 0 | 0 | 0 | 0 |
| *Stagonolepis robertsoni* | 0 | 0 | 0 | 1 | 0 | 0 | 0 | 0 | 0 | 0 | 0 | 0 | 0 | 0 | 0 |
| *Turfanosuchus dabanensis* | 0 | 1 | 0 | ? | 0 | 0 | 0 | 0 | 0 | 0 | 0 | 0 | 0 | 0 | 0 |
| *Gracilisuchus stipanicicorum* | 0 | 1 | 0 | 1 | 0 | 1 | 0 | 0 | 0 | 0 | 1 | ? | 0 | 0 | 1 |
| *Ticinosuchus ferox* | ? | ? | 0 | ? | ? | ? | ? | ? | ? | ? | ? | ? | ? | ? | ? |
| *Qianosuchus mixtus* | 0 | 0 | 0 | 0 | 0 | 0 | 0 | 0 | 0 | 0 | 0 | 0 | 0 | 0 | 0 |
| *Arizonasaurus babbitti* | 0 | 0 | 0 | ? | 0 | 0 | 0 | ? | 0 | 0 | 0 | 0 | 0 | 0 | 0 |
| *Xilousuchus sapingensis* | ? | ? | ? | ? | ? | ? | ? | ? | ? | ? | ? | ? | ? | ? | ? |
| *Poposaurus gracilis* | ? | ? | ? | ? | ? | ? | ? | ? | ? | ? | ? | ? | ? | ? | ? |
| *Lotosaurus adentus* | 0 | 0 | 0 | 0 | 1 | 0 | 0 | 0 | 0 | 0 | 0 | 1 | 0 | 0 | 0 |
| *Sillosuchus longicervix* | ? | ? | ? | ? | ? | ? | ? | ? | ? | ? | ? | ? | ? | ? | ? |
| *Shuvosaurus inexpectatus* | ? | 1 | 1 | 0 | 0 | 0 | 0 | 0 | 0 | 0 | 0 | 1 | 0 | 0 | 0 |
| *Effigia okeeffeae* | 0 | 1 | 1 | ? | 1 | 0 | 0 | 0 | 0 | 0 | 0 | 1 | 0 | 0 | 0 |
| *Prestosuchus* | 1 | 0 | 0 | 0 | 0 | 0 | ? | 1 | 1 | 0 | 0 | 0 | 0 | 0 | 0 |
| *Saurosuchus galilei* | 1 | 0 | 0 | 0 | 0 | 0 | 0 | 1 | 1 | 0 | 0 | ? | 0 | ? | 0 |
| *Batrachotomus kuperferzellensis* | 0 | 1 | 0 | 0 | 0 | 1 | 0 | 1 | ? | 0 | 0 | 0 | 0 | 0 | 0 |
| *Decuriasuchus quartacolonia* | 0 | 1 | 0 | 0 | 0 | 1 | 0 | 1 | 1 | 0 | 0 | 0 | 0 | 0 | 0 |
| *Fasolasuchus tenax* | ? | ? | ? | ? | ? | ? | ? | ? | ? | ? | ? | ? | ? | ? | ? |
| *Rauisuchus triradentes* | ? | ? | ? | ? | 0 | 1 | 0 | 1 | 2 | 0 | 0 | 0 | 0 | 0 | ? |
| *Postosuchus alisonae* | ? | ? | ? | ? | ? | 1 | ? | ? | ? | ? | ? | ? | ? | ? | ? |
| *Postosuchus kirkpatricki* | 1 | 1 | 0 | 0 | 0 | 1 | 0 | 0 | 2 | 0 | 0 | 0 | 1 | 0 | 0 |
| *Polonosuchus silesiacus* | 1 | ? | ? | 0 | 0 | 1 | ? | 0 | 2 | 0 | 0 | 0 | 1 | 0 | ? |
| *Carnufex carolinensis* | ? | ? | ? | ? | ? | ? | ? | ? | ? | ? | ? | ? | ? | ? | ? |
| CM 73372 | ? | ? | ? | ? | ? | ? | ? | ? | ? | ? | ? | ? | ? | ? | ? |
| *Redondavenator quayensis* | ? | ? | ? | ? | ? | ? | ? | ? | ? | ? | ? | ? | ? | ? | ? |
| *“Hesperosuchus agilis”* | 0 | 1 | 1 | 1 | 0 | 1 | 0 | ? | 0 | 0 | 1 | - | 0 | 1 | 0 |
| *Dromicosuchus grallator* | 0 | 1 | 1 | 1 | 0 | 1 | 0 | 0 | 0 | 0 | 1 | - | 0 | 1 | 0 |
| *Hesperosuchus agilis* HT | 0 | 1 | 1 | ? | 0 | ? | ? | ? | ? | ? | ? | ? | ? | ? | ? |
| *Sphenosuchus acutus* | 0 | 1 | 1 | 1 | 0 | 0 | 0 | 0 | 0 | 0 | 1 | - | 0 | 1 | 1 |
| *Terrestrisuchus gracilis* | 0 | 1 | 1 | 1 | 0 | 1 | 1 | 0 | 0 | 0 | ? | - | ? | 1 | ? |
| *Dibothrosuchus elaphros* | 0 | 1 | 1 | ? | 0 | 1 | 0 | 0 | 0 | 0 | 1 | - | 0 | 1 | 1 |
| *Litargosuchus leptorhynchus* | 0 | 1 | 1 | ? | 0 | 1 | 1 | ? | ? | 0 | 1 | ? | ? | 1 | 1 |
| *Kayentasuchus walker* | ? | ? | ? | ? | 0 | 1 | 1 | 1 | 0 | 1 | 1 | - | 0 | 1 | ? |
| *Junggarsuchus sloani* | ? | 1 | 1 | 1 | 0 | 1 | 1 | ? | 0 | ? | 1 | - | 0 | 1 | 1 |
| *Orthosuchus stormbergi* | ? | 1 | 1 | 1 | 0 | 1 | 1 | 0 | 0 | 1 | 1 | - | ? | 1 | 1 |
| *Protosuchus richardsoni* | 0 | 1 | 1 | 1 | 0 | 1 | 1 | ? | 0 | 1 | 1 | - | 0 | 1 | 1 |
| *Protosuchus haughtoni* | ? | 1 | 1 | 1 | 0 | 1 | 1 | ? | ? | 1 | 1 | - | 0 | 1 | 1 |
| *Alligator mississippiensis* | 0 | 1 | 1 | 1 | 0 | 1 | 0 | ? | 0 | 1 | 1 | - | 0 | 1 | 1 |
|  |  |  |  |  |  |  |  |  |  |  |  |  |  |  |  |
|  |  |  |  |  |  |  |  |  |  |  |  |  |  |  |  |
|  |  |  |  |  |  |  |  |  |  |  |  |  |  |  |  |
|  | 46 | 47 | 48 | 49 | 50 | 51 | 52 | 53 | 54 | 55 | 56 | 57 | 58 | 59 | 60 |
| *Euparkeri capensis* | 0 | 0 | 0 | 0 | 0 | 0 | 0 | 0/1 | 0 | 0 | 0 | 1 | 0 | 0 | 0 |
| *Machaeroprosopus pristinus* | 0 | 0 | 0 | 0 | 0 | 0 | 0 | 0 | 0 | 1 | 0 | 0 | 0 | 0 | 0 |
| *Riojasuchus tenulsceps* | 0 | 0 | 0 | 0 | 0 | 0 | 0 | 0 | 0 | 0 | 0 | 0 | 0 | 0 | 0 |
| *Revueltosaurus callenderi* | 0 | 0 | 0 | 0 | 0 | 1 | 1 | 1 | 0 | 0 | 0 | 1 | 0 | 0 | 0 |
| *Stagonolepis robertsoni* | 0 | 0 | 1 | 0 | 0 | 1 | 1 | ? | 0 | 0 | 0 | ? | 0 | 0 | 0 |
| *Turfanosuchus dabanensis* | 0 | 0 | ? | 0 | 0 | 0 | 1 | 1 | 0 | 0 | 0 | 1 | 0 | 0 | 0 |
| *Gracilisuchus stipanicicorum* | 0 | 0 | 0 | 0 | 0 | 1 | 1 | 1 | 1 | 1 | 0 | 1 | 0 | 1 | 0 |
| *Ticinosuchus ferox* | ? | ? | ? | ? | ? | ? | ? | ? | ? | ? | ? | ? | ? | ? | ? |
| *Qianosuchus mixtus* | 0 | 0 | 0 | 0 | 0 | 0 | 0 | 1 | 0 | 0 | 0 | 1 | 0 | 0 | 0 |
| *Arizonasaurus babbitti* | 0 | 0 | 0 | 0 | 0 | 0 | 0 | ? | 0 | 0 | 0 | 1 | 0 | 0 | 0 |
| *Xilousuchus sapingensis* | ? | ? | ? | ? | ? | ? | ? | ? | ? | ? | ? | ? | ? | ? | ? |
| *Poposaurus gracilis* | ? | ? | ? | ? | ? | ? | ? | ? | ? | ? | ? | ? | ? | ? | ? |
| *Lotosaurus adentus* | 0 | 0 | 0 | 0 | 0 | 0 | 0 | 1 | 0 | 0 | 0 | 1 | 0 | 0 | 0 |
| *Sillosuchus longicervix* | ? | ? | ? | ? | ? | ? | ? | ? | ? | ? | ? | ? | ? | ? | ? |
| *Shuvosaurus inexpectatus* | 2 | 0 | ? | 0 | 0 | 0 | 1 | ? | 0 | 0 | 0 | 0 | 0 | 0 | ? |
| *Effigia okeeffeae* | 2 | 0 | ? | ? | 0 | 0 | 1 | ? | ? | ? | 0 | 0 | 0 | 0 | ? |
| *Prestosuchus* | 0 | ? | 0 | 0 | 0 | 0 | 0 | ? | 0 | 0 | 0 | ? | 0 | 0 | 0 |
| *Saurosuchus galilei* | 0 | 0 | 0 | 0 | 0 | 0 | 0 | 1 | ? | ? | 0 | 1 | 0 | 0 | 0 |
| *Batrachotomus kuperferzellensis* | 1 | 0 | 0 | 0 | 1 | 0 | 0 | ? | 0 | 0 | 0 | 1 | 0 | 0 | 0 |
| *Decuriasuchus quartacolonia* | 0 | 0 | 0 | 0 | 1 | 0 | 0 | ? | 0 | 0 | 0 | 1 | 0 | 0 | 0 |
| *Fasolasuchus tenax* | ? | ? | ? | ? | ? | ? | ? | ? | ? | ? | ? | ? | ? | ? | ? |
| *Rauisuchus triradentes* | ? | ? | ? | ? | ? | ? | 0 | ? | ? | ? | 0 | 2 | ? | 1 | ? |
| *Postosuchus alisonae* | ? | ? | ? | ? | ? | ? | ? | ? | ? | ? | ? | ? | ? | ? | ? |
| *Postosuchus kirkpatricki* | 1 | 0 | 0 | 0 | 1 | 1 | 0 | 1 | 1 | 1 | 0 | 2 | 0 | 1 | 0 |
| *Polonosuchus silesiacus* | ? | ? | ? | 0 | ? | 1 | 0 | ? | 1 | 1 | 0 | 2 | ? | 1 | 0 |
| *Carnufex carolinensis* | ? | ? | ? | ? | 0 | ? | 0 | 1 | 1 | ? | 0 | ? | ? | ? | ? |
| CM 73372 | ? | ? | ? | ? | ? | ? | ? | ? | ? | ? | ? | ? | ? | ? | ? |
| *Redondavenator quayensis* | ? | ? | ? | ? | ? | ? | ? | ? | ? | ? | ? | ? | ? | ? | ? |
| *“Hesperosuchus agilis”* | 1 | 0 | 0 | 1 | 0 | 1 | 0 | 1 | 1 | 1 | 0 | 1 | ? | 1 | 1 |
| *Dromicosuchus grallator* | 1 | 0 | 0 | 1 | 0 | 1 | 0 | 1 | 1 | 1 | 0 | 1 | ? | 1 | 1 |
| *Hesperosuchus agilis* HT | 1 | ? | ? | ? | ? | ? | ? | ? | ? | ? | ? | 1 | 1 | 1 | 1 |
| *Sphenosuchus acutus* | 2 | 0 | 1 | 1 | 0 | 1 | 0 | 1 | 1 | 1 | 1 | 1 | 1 | 1 | 1 |
| *Terrestrisuchus gracilis* | 2 | 0 | 0 | ? | ? | 1 | 0 | 1 | 1 | 1 | 0 | 0 | 1 | 1 | 1 |
| *Dibothrosuchus elaphros* | 2 | 0 | 1 | ? | ? | 1 | ? | 1 | ? | ? | ? | ? | 1 | 1 | 1 |
| *Litargosuchus leptorhynchus* | 0 | 1 | 1 | ? | 0 | ? | 0 | 1 | 1 | 1 | 1 | 1 | ? | 1 | 1 |
| *Kayentasuchus walker* | 0 | 1 | 1 | ? | ? | ? | ? | 1 | ? | ? | ? | ? | 1 | 1 | ? |
| *Junggarsuchus sloani* | 2 | 1 | 1 | ? | 0 | ? | 0 | 1 | 1 | 1 | 1 | 1 | 1 | 1 | 1 |
| *Orthosuchus stormbergi* | 0 | 1 | 1 | 1 | ? | 1 | 0 | ? | 1 | 1 | 0 | 1 | 1 | 1 | 1 |
| *Protosuchus richardsoni* | 0 | 1 | 1 | 1 | 0 | 1 | 0 | 1 | 1 | 1 | ? | 1 | 1 | 1 | 1 |
| *Protosuchus haughtoni* | 0 | 1 | 1 | ? | 0 | 1 | 0 | 1 | 1 | 1 | 1 | 1 | ? | 1 | 1 |
| *Alligator mississippiensis* | 0 | 1 | 1 | 1 | 0 | 1 | 0 | - | 1 | 1 | 0 | 0 | 1 | 1 | 1 |
|  |  |  |  |  |  |  |  |  |  |  |  |  |  |  |  |
|  |  |  |  |  |  |  |  |  |  |  |  |  |  |  |  |
|  |  |  |  |  |  |  |  |  |  |  |  |  |  |  |  |
|  | 61 | 62 | 63 | 64 | 65 | 66 | 67 | 68 | 69 | 70 | 71 | 72 | 73 | 74 | 75 |
| *Euparkeri capensis* | 0 | 0 | 0 | 0 | 0 | 0 | 0 | 0 | 1 | 0 | 0 | 0 | 0 | 0 | 0 |
| *Machaeroprosopus pristinus* | 0 | 0 | 0 | 0 | ? | 0 | 0 | ? | 0 | 0 | 0 | 0 | 0 | 0 | 0 |
| *Riojasuchus tenulsceps* | 0 | 0 | 0 | 0 | ? | 0 | 0 | 1 | 1 | 0 | 1 | 0 | ? | 0 | 0 |
| *Revueltosaurus callenderi* | 0 | 0 | 0 | 0 | ? | 0 | 0 | 1 | 0 | 0 | 1 | 0 | 0 | 0 | 0 |
| *Stagonolepis robertsoni* | 0 | 1 | 0 | ? | 1 | 0 | ? | 1 | 0 | 0 | 1 | 0 | 0 | 0 | ? |
| *Turfanosuchus dabanensis* | 0 | 0 | 0 | 0 | ? | ? | 0 | 0 | 0 | 0 | 1 | 0 | 0 | 0 | 0 |
| *Gracilisuchus stipanicicorum* | 0 | 0 | 0 | ? | ? | ? | 0 | 1 | 0 | 0 | 1 | 1 | ? | ? | 0 |
| *Ticinosuchus ferox* | ? | ? | ? | ? | ? | ? | ? | ? | ? | ? | ? | ? | ? | ? | ? |
| *Qianosuchus mixtus* | 0 | 0 | 0 | ? | 1 | 0 | ? | 0 | 0 | 0 | 1 | 0 | ? | ? | ? |
| *Arizonasaurus babbitti* | 0 | 0 | 0 | 0 | ? | ? | 0 | 0 | 1 | 0 | 1 | 0 | 0 | 0 | 0 |
| *Xilousuchus sapingensis* | ? | ? | ? | ? | ? | ? | 0 | 0 | 1 | 0 | 1 | 0 | 0 | 0 | 0 |
| *Poposaurus gracilis* | ? | ? | ? | ? | ? | ? | ? | ? | ? | ? | ? | ? | ? | ? | ? |
| *Lotosaurus adentus* | 0 | 0 | 0 | 0 | ? | ? | 0 | 0 | 0 | 1 | 1 | 0 | 0 | 0 | 0 |
| *Sillosuchus longicervix* | ? | ? | ? | ? | ? | ? | ? | ? | ? | ? | ? | ? | ? | ? | ? |
| *Shuvosaurus inexpectatus* | 0 | 1 | 0 | 0 | ? | ? | 1 | 1 | 0 | 0 | 1 | 1 | 0 | 0 | 0 |
| *Effigia okeeffeae* | 0 | 1 | 0 | 0 | ? | 0 | 1 | 1 | 0 | 0 | 1 | 1 | 0 | 0 | 0 |
| *Prestosuchus* | 0 | 0 | 0 | 0 | ? | ? | 0 | 1 | 0 | 0 | 1 | 0 | 0 | 0 | 0 |
| *Saurosuchus galilei* | 0 | 0 | 0 | 0 | 0 | 0 | 0 | 1 | 0 | 0 | 1 | 0 | 0 | 1 | 0 |
| *Batrachotomus kuperferzellensis* | 0 | 0 | 0 | 1 | 0 | 0 | 0 | 1 | 0 | 0 | 1 | 0 | 0 | 1 | 0 |
| *Decuriasuchus quartacolonia* | 0 | 0 | 0 | ? | ? | ? | ? | ? | ? | ? | ? | ? | ? | ? | ? |
| *Fasolasuchus tenax* | ? | ? | ? | ? | ? | ? | ? | ? | ? | ? | ? | ? | ? | ? | ? |
| *Rauisuchus triradentes* | ? | ? | ? | 1 | ? | ? | ? | ? | ? | ? | ? | ? | ? | ? | ? |
| *Postosuchus alisonae* | ? | ? | ? | ? | ? | ? | ? | ? | ? | ? | ? | ? | 1 | ? | ? |
| *Postosuchus kirkpatricki* | 0 | 0 | 1 | 1 | ? | ? | 0 | 1 | 0 | 0 | 1 | ? | 1 | 1 | 0 |
| *Polonosuchus silesiacus* | 0 | 0 | 1 | 1 | 1 | 1 | ? | ? | ? | 0 | ? | ? | ? | ? | ? |
| *Carnufex carolinensis* | ? | ? | ? | ? | ? | ? | ? | ? | ? | ? | ? | ? | ? | ? | ? |
| CM 73372 | ? | ? | ? | ? | ? | ? | ? | ? | ? | ? | ? | ? | ? | ? | ? |
| *Redondavenator quayensis* | ? | ? | ? | ? | ? | ? | ? | ? | ? | ? | ? | ? | ? | ? | ? |
| *“Hesperosuchus agilis”* | 0 | 0 | 0 | 1 | 1 | ? | ? | ? | ? | ? | ? | ? | ? | 1 | ? |
| *Dromicosuchus grallator* | 0 | 0 | 0 | ? | ? | ? | ? | ? | ? | ? | ? | ? | ? | ? | ? |
| *Hesperosuchus agilis* HT | 0 | 0 | 0 | ? | ? | ? | ? | ? | ? | ? | 1 | ? | 1 | ? | 0 |
| *Sphenosuchus acutus* | 0 | 0 | 0 | 1 | 1 | 1 | 0 | 1 | 0 | 0 | 1 | ? | 1 | 1 | 1 |
| *Terrestrisuchus gracilis* | 0 | 0 | 0 | ? | 0 | ? | ? | ? | ? | ? | ? | ? | ? | 1 | 1 |
| *Dibothrosuchus elaphros* | 0 | 0 | 0 | ? | ? | 1 | ? | 1 | 0 | 0 | 1 | 1 | 1 | 1 | 1 |
| *Litargosuchus leptorhynchus* | 0 | 0 | 0 | ? | ? | ? | ? | ? | ? | ? | ? | ? | ? | ? | ? |
| *Kayentasuchus walker* | ? | ? | ? | ? | 0 | 1 | ? | ? | ? | ? | ? | ? | 1 | ? | ? |
| *Junggarsuchus sloani* | 1 | 0 | 0 | ? | ? | ? | ? | ? | ? | ? | ? | ? | ? | ? | ? |
| *Orthosuchus stormbergi* | 1 | 0 | 0 | 1 | ? | 0 | - | ? | 0 | 1 | 0 | ? | ? | 1 | 1 |
| *Protosuchus richardsoni* | 1 | 0 | 0 | 1 | 0 | 0 | - | 1 | 0 | 1 | 0 | ? | 1 | 1 | 1 |
| *Protosuchus haughtoni* | 1 | 0 | 0 | ? | ? | ? | - | 1 | 0 | 1 | 0 | ? | ? | 1 | 1 |
| *Alligator mississippiensis* | 1 | 0 | 0 | ? | ? | ? | - | 1 | 0 | 1 | 0 | ? | 1 | 1 | 0 |
|  |  |  |  |  |  |  |  |  |  |  |  |  |  |  |  |
|  |  |  |  |  |  |  |  |  |  |  |  |  |  |  |  |
|  |  |  |  |  |  |  |  |  |  |  |  |  |  |  |  |
|  | 76 | 77 | 78 | 79 | 80 | 81 | 82 | 83 | 84 | 85 | 86 | 87 | 88 | 89 | 90 |
| *Euparkeri capensis* | 0 | 0 | 0 | 0 | 0 | 0 | 0 | 0 | 0 | 0 | 0 | 0 | 0 | 0 | 0 |
| *Machaeroprosopus pristinus* | 0 | 0 | 0 | 0 | 0 | 0 | 0 | 0 | 0 | 0 | 0 | 0 | 0 | 0 | 0 |
| *Riojasuchus tenulsceps* | 0 | 0 | 0 | 0 | ? | 0 | 0 | ? | ? | ? | ? | 0 | ? | 0 | ? |
| *Revueltosaurus callenderi* | 0 | 0 | 0 | ? | 0 | 2 | 0 | 0 | ? | 0 | 0 | 0 | 2 | 0 | 0 |
| *Stagonolepis robertsoni* | 0 | 0 | 0 | 1 | 0 | 2 | ? | 0 | 1 | 0 | 0 | 0 | ? | 0 | 0 |
| *Turfanosuchus dabanensis* | ? | 0 | 0 | 0 | 0 | 2 | ? | ? | ? | ? | 0 | 0 | 1 | 0 | 0 |
| *Gracilisuchus stipanicicorum* | 0/1 | 0 | 0 | 0 | ? | ? | 0/1 | ? | ? | ? | ? | ? | ? | 0 | ? |
| *Ticinosuchus ferox* | ? | ? | ? | ? | ? | ? | ? | ? | ? | ? | ? | ? | ? | ? | ? |
| *Qianosuchus mixtus* | 0 | 0 | 0 | ? | ? | ? | ? | ? | ? | ? | ? | ? | ? | 0 | ? |
| *Arizonasaurus babbitti* | 0 | 0 | 0 | 0 | 0 | 0 | 0 | 0 | 1 | 0 | 0 | 0 | 1 | 0 | 0 |
| *Xilousuchus sapingensis* | 0 | ? | 0 | 0 | 0 | 0 | 0 | 0 | 0 | ? | 0 | 0 | 1 | 0 | 0 |
| *Poposaurus gracilis* | ? | ? | ? | ? | ? | ? | ? | ? | ? | ? | ? | ? | ? | ? | ? |
| *Lotosaurus adentus* | 0 | 0 | 0 | 0 | 0 | 0 | 0 | ? | ? | ? | 0 | 0 | 1 | 0 | 0 |
| *Sillosuchus longicervix* | ? | ? | ? | ? | ? | ? | ? | ? | ? | ? | ? | ? | ? | ? | ? |
| *Shuvosaurus inexpectatus* | 0 | 0 | 1 | 0 | 0 | 0 | 1 | 0 | 1 | 0 | 0 | 0 | ? | 0 | 0 |
| *Effigia okeeffeae* | 0 | 0 | ? | ? | ? | ? | 1 | 0 | 1 | 0 | 0 | 0 | ? | 0 | 0 |
| *Prestosuchus* | 1 | 0 | 0 | 0 | 0 | 2 | 0 | ? | ? | 0 | 0 | 0 | 1 | 0 | 0 |
| *Saurosuchus galilei* | 0 | 0 | 0 | 0 | 0 | 2 | 0 | 0 | 1 | ? | 0 | 0 | ? | 0 | 0 |
| *Batrachotomus kuperferzellensis* | 1 | 0 | 0 | 0 | 0 | ? | 0 | 0 | 1 | 0 | 0 | 0 | 1 | 0 | 0 |
| *Decuriasuchus quartacolonia* | ? | ? | ? | ? | ? | ? | ? | ? | ? | ? | ? | ? | ? | ? | ? |
| *Fasolasuchus tenax* | ? | ? | ? | ? | ? | ? | ? | ? | ? | ? | ? | ? | ? | ? | ? |
| *Rauisuchus triradentes* | ? | 0 | ? | ? | ? | ? | ? | ? | ? | ? | ? | ? | ? | ? | ? |
| *Postosuchus alisonae* | 1 | ? | 0 | ? | ? | ? | ? | ? | 1 | ? | ? | ? | ? | ? | 1 |
| *Postosuchus kirkpatricki* | 1 | 0 | 0 | 1 | 0 | 2 | 0 | 0 | 1 | 0 | 0 | 1 | ? | 0 | 1 |
| *Polonosuchus silesiacus* | ? | ? | ? | ? | ? | ? | ? | ? | ? | ? | ? | ? | ? | ? | ? |
| *Carnufex carolinensis* | ? | ? | ? | ? | ? | ? | ? | ? | ? | ? | ? | ? | ? | ? | ? |
| CM 73372 | ? | ? | ? | ? | ? | ? | ? | ? | ? | ? | ? | ? | ? | ? | ? |
| *Redondavenator quayensis* | ? | ? | ? | ? | ? | ? | ? | ? | ? | ? | ? | ? | ? | ? | ? |
| *“Hesperosuchus agilis”* | 1 | 0 | 0 | ? | ? | ? | 1 | ? | ? | ? | ? | 1 | ? | ? | ? |
| *Dromicosuchus grallator* | 1 | 0 | 0 | ? | ? | ? | ? | ? | ? | ? | ? | ? | ? | ? | ? |
| *Hesperosuchus agilis* HT | 1 | 0 | 0 | ? | ? | 1 | 1 | 1 | ? | ? | ? | ? | ? | ? | ? |
| *Sphenosuchus acutus* | 1 | 0 | 0 | 1 | 1 | 1 | 1 | 1 | 1 | 1 | 1 | 1 | 2 | 1 | 0 |
| *Terrestrisuchus gracilis* | ? | ? | 0 | ? | ? | ? | 1 | ? | ? | ? | ? | ? | ? | ? | 0 |
| *Dibothrosuchus elaphros* | 1 | 0 | 0 | 1 | 1 | 1 | 1 | 1 | ? | ? | 1 | 1 | 2 | 1 | 0 |
| *Litargosuchus leptorhynchus* | 1 | 1 | 0 | ? | ? | ? | ? | ? | ? | ? | ? | ? | ? | ? | ? |
| *Kayentasuchus walker* | ? | 1 | ? | ? | ? | ? | ? | 1 | 1 | ? | ? | ? | ? | ? | 0 |
| *Junggarsuchus sloani* | 1 | ? | ? | ? | ? | ? | ? | ? | ? | ? | ? | ? | ? | ? | ? |
| *Orthosuchus stormbergi* | 1 | 1 | 0 | ? | ? | 1 | 1 | ? | ? | ? | ? | 2 | ? | ? | ? |
| *Protosuchus richardsoni* | 1 | 1 | 0 | 1 | 1 | 1 | 1 | 1 | 1 | 1 | ? | 2 | ? | ? | 0 |
| *Protosuchus haughtoni* | 1 | 1 | 0 | ? | ? | ? | 1 | ? | ? | ? | ? | 2 | ? | ? | ? |
| *Alligator mississippiensis* | 0 | 0 | 0 | 1 | 1 | 1 | 1 | 1 | 1 | 1 | 1 | 2 | 2 | ? | 0 |
|  |  |  |  |  |  |  |  |  |  |  |  |  |  |  |  |
|  |  |  |  |  |  |  |  |  |  |  |  |  |  |  |  |
|  |  |  |  |  |  |  |  |  |  |  |  |  |  |  |  |
|  | 91 | 92 | 93 | 94 | 95 | 96 | 97 | 98 | 99 | 100 | 101 | 102 | 103 | 104 | 105 |
| *Euparkeri capensis* | 0 | 0 | 0 | 0 | 0 | 0 | 0 | 0 | 1 | 0 | 0 | 0 | 0 | 0 | 0 |
| *Machaeroprosopus pristinus* | 0 | 0 | 0 | 0 | 0 | 0 | 0 | 0 | 0 | 0 | 0 | 0 | 0 | ? | 0 |
| *Riojasuchus tenulsceps* | ? | ? | ? | ? | ? | ? | ? | 1 | 2 | 0 | ? | 0 | 0 | 0 | 0 |
| *Revueltosaurus callenderi* | ? | ? | ? | ? | ? | 0 | ? | 0 | 2 | 0 | 0 | 0 | ? | 0 | 0 |
| *Stagonolepis robertsoni* | ? | 0 | 1 | 0 | 1 | 0 | ? | 1 | 2 | 0 | 0 | 0 | ? | ? | 1 |
| *Turfanosuchus dabanensis* | ? | 0 | ? | 0 | 0 | ? | ? | 0 | 1 | ? | 0 | 0 | 0 | ? | 0 |
| *Gracilisuchus stipanicicorum* | 0 | ? | ? | ? | ? | 0 | ? | 0 | 1 | ? | 0 | 0 | 0 | 1 | 0 |
| *Ticinosuchus ferox* | ? | ? | ? | ? | ? | ? | ? | ? | 1 | ? | ? | ? | ? | ? | 0 |
| *Qianosuchus mixtus* | ? | ? | ? | ? | ? | ? | ? | 1 | ? | ? | 0 | 0 | 0 | ? | 0 |
| *Arizonasaurus babbitti* | ? | 0 | 0 | 0 | 0 | 1 | 0 | 1 | 0 | ? | 0 | 0 | ? | 0 | 0 |
| *Xilousuchus sapingensis* | ? | 0 | 0 | 0 | 0 | ? | 0 | 1 | 0 | ? | ? | ? | ? | 0 | 0 |
| *Poposaurus gracilis* | ? | ? | ? | ? | ? | ? | ? | ? | ? | ? | ? | ? | ? | ? | ? |
| *Lotosaurus adentus* | ? | 0 | 0 | 0 | 0 | ? | ? | 1 | ? | 1 | 0 | 0 | 0 | ? | 0 |
| *Sillosuchus longicervix* | ? | ? | ? | ? | ? | ? | ? | ? | ? | ? | ? | ? | ? | ? | ? |
| *Shuvosaurus inexpectatus* | 0 | 0 | ? | ? | 0 | 0 | 0 | 0 | ? | ? | 0 | 0 | 0 | 0 | 0 |
| *Effigia okeeffeae* | ? | 0 | ? | ? | 0 | ? | 0 | 0 | 1 | ? | 0 | 0 | 0 | ? | 0 |
| *Prestosuchus* | 0 | 0 | ? | 0 | 0 | ? | ? | 2 | 1 | 0 | 1 | 0 | 0 | 0 | 0 |
| *Saurosuchus galilei* | 0 | 0 | ? | 0 | 0 | ? | ? | 2 | 1 | 0 | 1 | 0 | 0 | ? | 0 |
| *Batrachotomus kuperferzellensis* | 0 | 0 | 0 | 0 | 1 | 1 | 1 | 1 | 2 | 1 | 1 | 1 | 0 | 0 | 0 |
| *Decuriasuchus quartacolonia* | ? | ? | ? | ? | ? | ? | ? | 2 | ? | ? | 1 | ? | 0 | 1 | 0 |
| *Fasolasuchus tenax* | ? | ? | ? | ? | ? | ? | ? | ? | 2 | ? | ? | ? | ? | 0 | 0 |
| *Rauisuchus triradentes* | ? | ? | ? | ? | ? | ? | ? | 2 | ? | ? | ? | ? | ? | ? | ? |
| *Postosuchus alisonae* | ? | ? | ? | ? | ? | ? | 1 | ? | ? | ? | ? | ? | 1 | ? | ? |
| *Postosuchus kirkpatricki* | ? | 0 | ? | ? | ? | 1 | ? | 2 | 1 | ? | 1 | 1 | 1 | 0 | 1 |
| *Polonosuchus silesiacus* | ? | ? | ? | ? | ? | ? | ? | 2 | 1 | ? | 1 | ? | 1 | 1 | 1 |
| *Carnufex carolinensis* | ? | ? | ? | ? | ? | ? | ? | 0 | 1 | ? | 1 | ? | ? | ? | ? |
| CM 73372 | ? | ? | ? | ? | ? | ? | ? | ? | ? | ? | ? | ? | ? | ? | ? |
| *Redondavenator quayensis* | ? | ? | ? | ? | ? | ? | ? | 0 | ? | ? | ? | ? | ? | ? | ? |
| *“Hesperosuchus agilis”* | ? | ? | ? | ? | ? | ? | ? | 0 | 2 | ? | 0 | 1 | 1 | ? | 1 |
| *Dromicosuchus grallator* | ? | ? | ? | ? | ? | ? | ? | 0 | 2 | ? | 0 | 1 | 1 | 0 | ? |
| *Hesperosuchus agilis* HT | ? | ? | ? | ? | ? | ? | ? | ? | 1 | ? | ? | 1 | ? | 0 | 1 |
| *Sphenosuchus acutus* | 0 | 1 | 1 | 1 | 1 | 1 | 1 | 0 | 1 | 1 | 0 | 1 | ? | - | 1 |
| *Terrestrisuchus gracilis* | ? | ? | ? | ? | ? | ? | ? | 0 | 1 | ? | 0 | 1 | ? | ? | 0 |
| *Dibothrosuchus elaphros* | 0 | 1 | 1 | 1 | ? | ? | ? | 0 | 1 | ? | 0 | 1 | ? | ? | 0 |
| *Litargosuchus leptorhynchus* | 0 | ? | ? | ? | ? | ? | ? | 0 | 1 | ? | 0 | 0 | 1 | ? | ? |
| *Kayentasuchus walker* | ? | ? | ? | ? | ? | ? | 1 | 0 | 2 | ? | ? | ? | ? | 1 | 0 |
| *Junggarsuchus sloani* | ? | ? | ? | ? | ? | ? | ? | 0 | ? | ? | 0 | 0 | 1 | ? | ? |
| *Orthosuchus stormbergi* | ? | ? | ? | ? | ? | ? | ? | 0 | 2 | ? | 0 | 0 | 1 | ? | 1 |
| *Protosuchus richardsoni* | 1 | 1 | ? | ? | 1 | ? | ? | 0 | 1 | 1 | 0 | 0 | 1 | ? | 0 |
| *Protosuchus haughtoni* | ? | ? | ? | ? | ? | ? | ? | ? | ? | ? | 0 | 0 | 1 | ? | 0 |
| *Alligator mississippiensis* | 1 | 1 | 1 | 1 | 0 | 1 | ? | 0 | - | 0 | 0 | 1 | 1 | 1 | 0 |
|  |  |  |  |  |  |  |  |  |  |  |  |  |  |  |  |
|  |  |  |  |  |  |  |  |  |  |  |  |  |  |  |  |
|  |  |  |  |  |  |  |  |  |  |  |  |  |  |  |  |
|  | 106 | 107 | 108 | 109 | 110 | 111 | 112 | 113 | 114 | 115 | 116 | 117 | 118 | 119 | 120 |
| *Euparkeri capensis* | 0 | 0 | 0 | ? | 0 | 0 | 0 | 0 | 0 | 0 | 0 | 0 | 0 | 0 | 0 |
| *Machaeroprosopus pristinus* | 0 | 0 | 0 | ? | 0 | 0 | 0 | 1 | 0 | 0 | ? | 0 | 0 | 0 | 0 |
| *Riojasuchus tenulsceps* | 0 | ? | 0 | ? | 0 | 0 | 0 | 1 | 0 | 0 | 0 | 0 | 0 | 0 | 0 |
| *Revueltosaurus callenderi* | 0 | ? | 0 | ? | 0 | 0 | 0 | 0 | 0 | 0 | 0 | 0 | 0 | 0 | 0 |
| *Stagonolepis robertsoni* | 1 | 0 | 1 | 0 | 0 | 1 | ? | 0 | 0 | 0 | ? | 0 | 1 | ? | ? |
| *Turfanosuchus dabanensis* | 0 | ? | 0 | ? | ? | 0 | ? | 0 | 0 | 0 | ? | 0 | 0 | ? | ? |
| *Gracilisuchus stipanicicorum* | 0 | ? | 0 | ? | 0 | 0 | ? | 0 | 0 | 0 | 0 | 0 | 0 | 0 | 1 |
| *Ticinosuchus ferox* | 0 | ? | ? | ? | ? | ? | ? | ? | 0 | ? | ? | ? | 0 | ? | 0 |
| *Qianosuchus mixtus* | 0 | ? | ? | ? | ? | 0 | ? | ? | 0 | 0 | 0 | 0 | 0 | ? | 1 |
| *Arizonasaurus babbitti* | 0 | 0 | 1 | ? | 1 | 0 | 0 | 0 | 0 | ? | ? | 0 | 0 | 0 | 1 |
| *Xilousuchus sapingensis* | 0 | 0 | 1 | ? | 1 | 0 | 0 | 0 | 0 | ? | ? | 0 | 0 | 0 | ? |
| *Poposaurus gracilis* | ? | ? | 1 | ? | ? | ? | ? | ? | ? | ? | ? | ? | 0 | ? | ? |
| *Lotosaurus adentus* | 0 | ? | 1 | ? | 0 | 1 | 0 | 0 | 0 | ? | 0 | 1 | - | 0 | 0 |
| *Sillosuchus longicervix* | ? | ? | ? | ? | ? | ? | ? | ? | ? | ? | ? | ? | ? | ? | 1 |
| *Shuvosaurus inexpectatus* | 0 | ? | 0 | ? | 0 | 1 | 1 | 0 | 1 | ? | 1 | 1 | - | 0 | 0 |
| *Effigia okeeffeae* | 0 | 0 | 0 | ? | 0 | 1 | 1 | 0 | 1 | 0 | 1 | 1 | - | ? | 0 |
| *Prestosuchus* | 0 | ? | 0 | ? | 1 | 0 | 0 | 0 | 0 | 0 | 0 | 0 | 0 | 0 | 0 |
| *Saurosuchus galilei* | 0 | ? | ? | ? | ? | ? | ? | ? | ? | ? | ? | 0 | 0 | 0 | 0 |
| *Batrachotomus kuperferzellensis* | 0 | 1 | 1 | 0 | 1 | 0 | 0 | 0 | 0 | 0 | 0 | 0 | 0 | 0 | 0 |
| *Decuriasuchus quartacolonia* | 0 | 1 | 1 | 0 | 1 | 0 | 0 | ? | 0 | 0 | 0 | 0 | 0 | ? | ? |
| *Fasolasuchus tenax* | 0 | 1 | 1 | ? | 1 | ? | 0 | ? | ? | ? | 0 | 0 | 0 | 0 | 0 |
| *Rauisuchus triradentes* | ? | 1 | 1 | ? | ? | 0 | 0 | 0 | ? | ? | 0 | ? | 0 | 1 | 0 |
| *Postosuchus alisonae* | ? | 1 | 1 | 0 | 1 | ? | 0 | ? | ? | ? | ? | ? | 0 | 1 | 0 |
| *Postosuchus kirkpatricki* | 1 | 1 | 1 | 0 | 1 | 0 | 0 | - | 0 | 0 | 0 | 0 | 0 | ? | ? |
| *Polonosuchus silesiacus* | 1 | 2 | 1 | 0 | 1 | ? | 0 | ? | ? | ? | ? | 0 | 0 | 1 | ? |
| *Carnufex carolinensis* | ? | 1 | 1 | 1 | 1 | ? | 0 | ? | 0 | 1 | ? | ? | 0 | ? | ? |
| CM 73372 | ? | ? | ? | ? | ? | ? | ? | ? | ? | ? | ? | ? | ? | ? | 0 |
| *Redondavenator quayensis* | ? | ? | ? | ? | ? | ? | ? | ? | ? | ? | ? | ? | 0 | ? | ? |
| *“Hesperosuchus agilis”* | 1 | 0 | 2 | 1 | 1 | 0 | ? | ? | 0 | 0 | 2 | 0 | 0 | 0 | 0 |
| *Dromicosuchus grallator* | ? | 0 | 2 | 1 | 1 | 0 | 0 | ? | 0 | 0 | 2 | 0 | 0 | ? | 1 |
| *Hesperosuchus agilis* HT | 1 | ? | ? | ? | ? | ? | ? | ? | ? | ? | ? | 0 | 0 | 0 | 0 |
| *Sphenosuchus acutus* | 1 | 0 | 2 | 1 | 1 | 0 | 0 | 0 | 0 | 0 | 2 | 0 | 0 | 0 | 0 |
| *Terrestrisuchus gracilis* | 0 | 0 | 2 | 1 | ? | 0 | ? | 0 | 0 | 0 | 2 | 0 | 0 | 0 | 0 |
| *Dibothrosuchus elaphros* | 0 | 0 | 2 | 1 | ? | 0 | ? | 0 | 0 | ? | 2 | 0 | 0 | 0 | 0 |
| *Litargosuchus leptorhynchus* | ? | ? | 2 | ? | ? | 0 | ? | 0 | 0 | 0 | ? | ? | 0 | ? | 0 |
| *Kayentasuchus walker* | 0 | ? | ? | ? | ? | ? | ? | 1 | 0 | ? | ? | 0 | 0 | ? | ? |
| *Junggarsuchus sloani* | ? | ? | 2 | 1 | ? | 0 | ? | ? | 0 | 1 | ? | 0 | ? | ? | ? |
| *Orthosuchus stormbergi* | 1 | 0 | 2 | ? | ? | 0 | ? | 0 | 0 | ? | ? | 0 | 1 | 0 | 0 |
| *Protosuchus richardsoni* | 0 | 0 | 2 | 1 | 1 | 0 | 0 | 1 | 2 | 0 | 2 | 0 | 0 | 1 | 0 |
| *Protosuchus haughtoni* | 0 | 0 | 2 | 1 | 1 | 0 | 0 | 1 | 2 | 0 | ? | 0 | 0 | 1 | 0 |
| *Alligator mississippiensis* | 0 | 0 | 0 | 0 | 0 | 0 | 1 | 0 | 0 | 1 | 2 | 0 | 1 | 0 | 0 |
|  |  |  |  |  |  |  |  |  |  |  |  |  |  |  |  |
|  |  |  |  |  |  |  |  |  |  |  |  |  |  |  |  |
|  |  |  |  |  |  |  |  |  |  |  |  |  |  |  |  |
|  | 121 | 122 | 123 | 124 | 125 | 126 | 127 | 128 | 129 | 130 | 131 | 132 | 133 | 134 | 135 |
| *Euparkeri capensis* | 0 | 0 | 0 | 0 | 0 | 1 | 0 | 0 | 0 | 0 | 0 | 0 | 0 | 0 | 0 |
| *Machaeroprosopus pristinus* | 0 | 0 | 0 | 0 | 0 | 1 | 0 | 0 | 0 | 0 | 1 | 0 | 0 | 0 | 0 |
| *Riojasuchus tenulsceps* | 0 | 0 | 0 | 0 | 1 | 1 | 1 | 0 | 0 | 0 | ? | 0 | 0 | 1 | 0 |
| *Revueltosaurus callenderi* | 0 | 1 | 0 | 0 | 0 | 2 | 0 | 0 | 0 | 0 | 1 | 0 | 0 | 1 | 0 |
| *Stagonolepis robertsoni* | ? | ? | 0 | 0 | 0 | 1 | 0 | 0 | 0 | 1 | 1 | 0 | 0 | 1 | 0 |
| *Turfanosuchus dabanensis* | ? | ? | 0 | 0 | ? | 1 | 1 | 0 | 0 | ? | ? | 0 | 0 | ? | 0 |
| *Gracilisuchus stipanicicorum* | 0 | 0 | 0 | 0 | 0 | 1 | 1 | 0 | 0 | ? | 1 | 0 | 0 | 0 | 0 |
| *Ticinosuchus ferox* | 0 | 0 | 0 | 0 | 0 | ? | 0 | ? | 0 | 1 | 1 | 0 | 0 | 0 | 0 |
| *Qianosuchus mixtus* | 1 | 0 | 0 | 0 | 0 | 0 | 0 | ? | 0 | 1 | 0 | 1 | 0 | 0 | ? |
| *Arizonasaurus babbitti* | ? | 0 | 0 | 0 | 0 | 0 | 0 | 1 | 1 | 1 | 0 | 1 | 1 | 0 | ? |
| *Xilousuchus sapingensis* | 1 | 1 | 0 | 0 | 0 | 0 | 0 | 0 | 1 | 1 | 0 | ? | ? | ? | ? |
| *Poposaurus gracilis* | ? | ? | 0 | 0 | ? | 0 | 0 | 1 | 0 | 1 | 0 | 1 | 0 | 0 | 1 |
| *Lotosaurus adentus* | 0 | 0 | 0 | 0 | 0 | 0 | 0 | ? | 1 | 1 | ? | 1 | 1 | 0 | 1 |
| *Sillosuchus longicervix* | ? | ? | 1 | 1 | 0 | 0 | 0 | 0 | 0 | ? | ? | 1 | 0 | 0 | 1 |
| *Shuvosaurus inexpectatus* | 0 | 0 | 1 | 1 | 1 | 0 | 0 | ? | ? | ? | ? | 1 | 0 | ? | 1 |
| *Effigia okeeffeae* | ? | ? | 1 | 1 | 1 | 0 | 0 | ? | 0 | 1 | ? | 1 | 0 | 1 | 1 |
| *Prestosuchus* | 0 | 0 | 0 | 0 | 0 | ? | 0 | 0 | 0 | 1 | ? | 0 | 0 | 0 | 0 |
| *Saurosuchus galilei* | 0 | 0 | 0 | 0 | 0 | 2 | 0 | 0 | 0 | 1 | ? | 0 | 0 | 0 | 0 |
| *Batrachotomus kuperferzellensis* | 0 | 0 | 0 | 0 | 0 | 2 | 0 | 0 | 0 | 1 | 1 | 0 | 0 | 0 | 0 |
| *Decuriasuchus quartacolonia* | ? | ? | 0 | ? | ? | ? | ? | ? | ? | 1 | ? | ? | ? | 0 | ? |
| *Fasolasuchus tenax* | 0 | 0 | 0 | 0 | 0 | 1 | 0 | 0 | 0 | 1 | ? | 0 | 0 | 0 | ? |
| *Rauisuchus triradentes* | ? | 1 | 0 | 0 | 0 | ? | 0 | 0 | 0 | 1 | ? | 1 | 0 | 0 | ? |
| *Postosuchus alisonae* | 0 | ? | 1 | 0 | 0 | 2 | 1 | 0 | 0 | 1 | 1 | 1 | 0 | 0 | ? |
| *Postosuchus kirkpatricki* | ? | ? | 0 | 0 | 0 | ? | 1 | ? | 0 | 1 | ? | 1 | 0 | 0 | 0 |
| *Polonosuchus silesiacus* | ? | 0 | 0 | 0 | 0 | ? | ? | ? | ? | ? | ? | ? | ? | ? | ? |
| *Carnufex carolinensis* | ? | ? | 1 | ? | ? | ? | ? | ? | 0 | 1 | ? | 1 | 0 | 0 | ? |
| CM 73372 | ? | ? | 0 | 0 | 0 | ? | 0 | 0 | 0 | ? | 1 | 1 | 0 | 0 | 0 |
| *Redondavenator quayensis* | ? | ? | ? | ? | ? | ? | ? | ? | ? | ? | ? | ? | ? | ? | ? |
| *“Hesperosuchus agilis”* | 0 | ? | 0 | 0 | 0 | ? | 0 | 0 | ? | ? | 1 | ? | 0 | ? | ? |
| *Dromicosuchus grallator* | ? | ? | 0 | 0 | 0 | 0 | 0 | 0 | ? | ? | 1 | 0 | 0 | 0 | 0 |
| *Hesperosuchus agilis* HT | 0 | 0 | 0 | 0 | 0 | 1 | ? | 0 | 0 | ? | 1 | 0 | ? | 0 | ? |
| *Sphenosuchus acutus* | ? | 0 | 0 | 0 | 0 | ? | ? | 0 | 0 | ? | 1 | 1 | ? | ? | ? |
| *Terrestrisuchus gracilis* | 0 | 0 | 0 | 0 | 0 | 0 | 0 | 0 | 0 | ? | 1 | 1 | 0 | 0 | 0 |
| *Dibothrosuchus elaphros* | 0 | 0 | 0 | 0 | 0 | 1 | 0 | 0 | 0 | 0 | 1 | ? | ? | 0 | ? |
| *Litargosuchus leptorhynchus* | ? | 0 | 0 | 0 | 0 | 0 | 0 | 0 | 0 | ? | 1 | 1 | 0 | 0 | ? |
| *Kayentasuchus walker* | ? | ? | ? | ? | ? | ? | ? | ? | ? | ? | ? | ? | ? | ? | ? |
| *Junggarsuchus sloani* | ? | ? | ? | ? | ? | ? | ? | ? | ? | ? | ? | ? | ? | ? | ? |
| *Orthosuchus stormbergi* | 0 | 0 | 0 | 0 | 0 | ? | 0 | 0 | 0 | ? | 1 | 1 | 0 | 1 | 0 |
| *Protosuchus richardsoni* | 0 | 0 | 0 | 0 | 0 | 0 | 0 | ? | 0 | ? | 1 | 1 | 0 | 1 | 0 |
| *Protosuchus haughtoni* | 0 | ? | 0 | 0 | 0 | 0 | 0 | ? | ? | ? | 1 | 1 | 0 | 1 | ? |
| *Alligator mississippiensis* | 0 | 0 | 0 | 0 | ? | 1 | 1 | 0 | 0 | 0 | 1 | 1 | 0 | 1 | 0 |
|  |  |  |  |  |  |  |  |  |  |  |  |  |  |  |  |
|  |  |  |  |  |  |  |  |  |  |  |  |  |  |  |  |
|  |  |  |  |  |  |  |  |  |  |  |  |  |  |  |  |
|  | 136 | 137 | 138 | 139 | 140 | 141 | 142 | 143 | 144 | 145 | 146 | 147 | 148 | 149 | 150 |
| *Euparkeri capensis* | 0 | 0 | 0 | 0 | 0 | 0 | 0 | 0 | 0 | 0 | 0 | 1 | 0 | 0 | 0 |
| *Machaeroprosopus pristinus* | 0 | 0 | 0 | 0 | 0 | 0 | 0 | ? | ? | 0 | 0 | 0 | 1 | 0 | 0 |
| *Riojasuchus tenulsceps* | 0 | 0 | 0 | 1 | 0 | 0 | 1 | ? | ? | 0 | 0 | 0 | 0 | 2 | 0 |
| *Revueltosaurus callenderi* | 0 | 0 | 0 | 0 | 0 | 0 | 0 | 0 | 0 | 0 | 0 | 0 | 1 | 2 | 1 |
| *Stagonolepis robertsoni* | 0 | 0 | 0 | 0 | 0 | 0 | 0 | 0 | ? | 0 | 0 | 0 | 1 | 2 | 1 |
| *Turfanosuchus dabanensis* | 0 | 0 | 0 | 0 | 0 | 0 | 0 | 0 | 0 | 0 | 1 | 1 | ? | 0 | 0 |
| *Gracilisuchus stipanicicorum* | 0 | 0 | 0 | 0 | 0 | 0 | 0 | 0 | 0 | 0 | 1 | 1 | 0 | 0 | ? |
| *Ticinosuchus ferox* | 0 | ? | 0 | 0 | 0 | 0 | 0 | 1 | 0 | 0 | 1 | 0 | 0 | 0 | 0 |
| *Qianosuchus mixtus* | 0 | 1 | ? | 0 | 0 | 1 | 0 | 1 | 0 | 0 | 1 | ? | 0 | 0 | 0 |
| *Arizonasaurus babbitti* | 1 | 1 | 0 | 0 | 0 | 1 | 0 | ? | ? | 1 | - | - | - | - | - |
| *Xilousuchus sapingensis* | ? | ? | ? | ? | ? | ? | ? | ? | ? | ? | ? | ? | ? | ? | ? |
| *Poposaurus gracilis* | 1 | 1 | 0 | 1 | 1 | 1 | ? | 0 | 1 | 1 | - | - | - | - | - |
| *Lotosaurus adentus* | ? | 1 | ? | 0 | 0 | 1 | 0 | ? | ? | 1 | - | - | - | - | - |
| *Sillosuchus longicervix* | 1 | 1 | 0 | 1 | 1 | 1 | 1 | ? | ? | 1 | - | - | - | - | - |
| *Shuvosaurus inexpectatus* | 1 | 1 | 1 | 1 | 0 | 1 | 1 | ? | 1 | 1 | - | - | - | - | - |
| *Effigia okeeffeae* | 1 | 1 | 1 | 1 | 0 | 1 | 1 | 0 | 1 | 1 | - | - | - | - | - |
| *Prestosuchus* | 0 | 0 | 0 | 0 | 0 | 0 | 0 | 1 | ? | 0 | 1 | 0 | ? | 0 | 0 |
| *Saurosuchus galilei* | ? | 0 | 0 | 0 | 0 | 0 | 0 | ? | ? | 0 | 1 | 0 | ? | 0 | 0 |
| *Batrachotomus kuperferzellensis* | 0 | 1 | 0 | 0 | 0 | 1 | 0 | 1 | 0 | 0 | 1 | 1 | ? | 1 | 0 |
| *Decuriasuchus quartacolonia* | ? | ? | ? | ? | ? | ? | ? | ? | ? | 0 | 1 | ? | ? | ? | ? |
| *Fasolasuchus tenax* | ? | ? | ? | ? | ? | ? | ? | ? | ? | 0 | ? | 1 | ? | ? | ? |
| *Rauisuchus triradentes* | ? | 0 | ? | 0 | 0 | 0 | ? | 1 | 0 | 0 | 1 | 1 | ? | 1 | 1 |
| *Postosuchus alisonae* | ? | ? | ? | ? | ? | ? | ? | ? | 0 | 0 | 1 | 1 | ? | 1 | 0 |
| *Postosuchus kirkpatricki* | 0 | 0 | 0 | 0 | 0 | 0 | 0 | ? | ? | 0 | 1 | 1 | ? | 1 | 0 |
| *Polonosuchus silesiacus* | ? | ? | ? | ? | ? | ? | ? | 1 | ? | 0 | 1 | ? | ? | ? | 0 |
| *Carnufex carolinensis* | ? | ? | ? | ? | ? | ? | ? | ? | ? | ? | ? | ? | ? | ? | ? |
| CM 73372 | ? | ? | 0 | 0 | 0 | 0 | 0 | 1 | ? | 0 | 1 | 1 | 0 | 1 | 0 |
| *Redondavenator quayensis* | ? | ? | ? | ? | ? | ? | ? | ? | ? | ? | ? | ? | ? | ? | ? |
| *“Hesperosuchus agilis”* | ? | ? | ? | ? | ? | ? | ? | 1 | ? | 0 | 1 | 1 | 0 | 1 | 0 |
| *Dromicosuchus grallator* | ? | 0 | 0 | 0 | 0 | 0 | 0 | ? | 0 | 0 | 1 | 1 | 0 | 1 | 0 |
| *Hesperosuchus agilis* HT | ? | 0 | ? | 0 | 0 | 0 | ? | ? | ? | 0 | 1 | 1 | ? | 1 | 0 |
| *Sphenosuchus acutus* | ? | ? | ? | ? | ? | ? | ? | ? | ? | 0 | ? | ? | ? | 1 | ? |
| *Terrestrisuchus gracilis* | 0 | 0 | 0 | 0 | 0 | 0 | 0 | 1 | 0 | 0 | ? | ? | 0 | 1 | 0 |
| *Dibothrosuchus elaphros* | ? | ? | ? | ? | ? | ? | ? | ? | ? | 0 | ? | 1 | ? | 1 | ? |
| *Litargosuchus leptorhynchus* | ? | ? | ? | ? | ? | ? | ? | 0 | 0 | 0 | 1 | ? | 0 | 0 | ? |
| *Kayentasuchus walker* | ? | ? | ? | ? | ? | ? | ? | ? | ? | 0 | 1 | ? | ? | ? | ? |
| *Junggarsuchus sloani* | ? | ? | ? | ? | ? | ? | ? | ? | ? | 1 | - | - | - | - | - |
| *Orthosuchus stormbergi* | ? | 0 | 0 | 0 | 0 | 0 | 0 | ? | ? | 0 | 1 | 0 | ? | 2 | 1 |
| *Protosuchus richardsoni* | ? | 0 | 0 | 0 | 0 | 0 | 0 | ? | ? | 0 | 1 | 0 | 1 | 2 | 1 |
| *Protosuchus haughtoni* | ? | ? | ? | ? | ? | ? | ? | ? | ? | 0 | 1 | 0 | ? | 2 | 1 |
| *Alligator mississippiensis* | 0 | 0 | 0 | 0 | 0 | 0 | 0 | 1 | 0 | 0 | 0 | 0 | 1 | 0/1 | ? |
|  |  |  |  |  |  |  |  |  |  |  |  |  |  |  |  |
|  |  |  |  |  |  |  |  |  |  |  |  |  |  |  |  |
|  |  |  |  |  |  |  |  |  |  |  |  |  |  |  |  |
|  | 151 | 152 | 153 | 154 | 155 | 156 | 157 | 158 | 159 | 160 | 161 | 162 | 163 | 164 | 165 |
| *Euparkeri capensis* | 0 | ? | 0 | 1 | 0 | 0 | 0 | 0 | 0 | 0 | 0 | 0 | 0 | 0 | 0 |
| *Machaeroprosopus pristinus* | 0 | 0 | 0 | 0 | 0 | 0 | 0 | 0 | 0 | 0 | 0 | 0 | 0 | 0 | 0 |
| *Riojasuchus tenulsceps* | 0 | ? | 0 | 0 | ? | ? | ? | 0 | 0 | 1 | 0 | 1 | 1 | 1 | 0 |
| *Revueltosaurus callenderi* | 1 | ? | 0 | 0 | 0 | ? | 0 | 0 | 0 | 1 | 0 | 1 | 0 | 1 | 0 |
| *Stagonolepis robertsoni* | 1 | 1 | 0 | 0 | ? | 0 | ? | 0 | 1 | 1 | ? | ? | 0 | 1 | 0 |
| *Turfanosuchus dabanensis* | ? | ? | 1 | ? | 0 | ? | ? | 0 | ? | ? | ? | ? | ? | ? | ? |
| *Gracilisuchus stipanicicorum* | 0 | ? | 0 | 0 | ? | ? | ? | 0 | ? | ? | ? | ? | ? | ? | ? |
| *Ticinosuchus ferox* | 0 | 1 | 1 | 1 | 0 | 0 | 0 | 0 | ? | ? | ? | 1 | 0 | ? | 0 |
| *Qianosuchus mixtus* | 0 | ? | 1 | 1 | ? | 0 | 0 | 0 | 0 | 1 | 0 | ? | ? | ? | ? |
| *Arizonasaurus babbitti* | - | - | - | - | ? | 0 | ? | 0 | 0 | 1 | ? | 1 | 0 | 1 | 0 |
| *Xilousuchus sapingensis* | ? | ? | ? | ? | ? | ? | ? | ? | ? | ? | ? | ? | ? | ? | ? |
| *Poposaurus gracilis* | 0 | - | - | - | 1 | 0 | 0 | ? | 0 | ? | ? | 1 | 0 | 1 | 0 |
| *Lotosaurus adentus* | - | - | - | - | 0 | ? | 0 | 0 | 0 | 0 | 0 | 0 | 0 | 1 | 1 |
| *Sillosuchus longicervix* | - | - | - | - | ? | ? | ? | 1 | ? | ? | ? | 1 | 0 | 0 | 1 |
| *Shuvosaurus inexpectatus* | - | - | - | - | 1 | ? | 0 | ? | 0 | ? | ? | 1 | 0 | 0 | 1 |
| *Effigia okeeffeae* | - | - | - | - | 1 | 0 | 0 | 1 | 0 | ? | ? | 1 | 0 | 0 | 1 |
| *Prestosuchus* | ? | ? | 1 | 1 | ? | 0 | 0 | 0 | 1 | 1 | 0 | 1 | 0 | 1 | 0 |
| *Saurosuchus galilei* | 0 | ? | 1 | 1 | ? | ? | ? | ? | ? | ? | ? | ? | ? | ? | ? |
| *Batrachotomus kuperferzellensis* | ? | ? | 0 | 0 | 0 | 0 | 0 | 0 | 1 | 1 | ? | 1 | 0 | 1 | 0 |
| *Decuriasuchus quartacolonia* | 0 | 1 | 1 | ? | 0 | ? | ? | ? | ? | ? | ? | ? | ? | ? | ? |
| *Fasolasuchus tenax* | ? | ? | ? | ? | ? | ? | ? | ? | ? | ? | ? | ? | ? | ? | ? |
| *Rauisuchus triradentes* | 0 | ? | 0 | 0 | ? | ? | ? | 0 | 1 | ? | ? | ? | ? | 1 | 1 |
| *Postosuchus alisonae* | 0 | ? | 0 | 0 | ? | 0 | ? | 0 | ? | ? | 1 | 1 | 1 | 1 | 1 |
| *Postosuchus kirkpatricki* | 0 | ? | 0 | ? | 0 | ? | 0 | 0 | 0 | 1 | 1 | 1 | ? | 1 | 1 |
| *Polonosuchus silesiacus* | ? | ? | ? | ? | ? | ? | ? | ? | ? | ? | ? | ? | ? | ? | ? |
| *Carnufex carolinensis* | ? | ? | ? | ? | ? | ? | ? | ? | ? | ? | ? | ? | ? | ? | ? |
| CM 73372 | 0 | 0 | 0 | ? | ? | ? | ? | 0 | ? | ? | ? | ? | ? | ? | ? |
| *Redondavenator quayensis* | ? | ? | ? | ? | ? | ? | ? | ? | 0 | ? | ? | 1 | ? | ? | ? |
| *“Hesperosuchus agilis”* | 0 | ? | 0 | 0 | ? | 1 | 0 | 0 | 0 | 1 | ? | ? | ? | ? | ? |
| *Dromicosuchus grallator* | 0 | ? | 0 | 0 | 0 | 1 | 0 | 0 | 0 | 1 | 1 | 2 | 1 | 1 | 1 |
| *Hesperosuchus agilis* HT | ? | ? | 0 | 0 | 0 | ? | 0 | 0 | 0 | 1 | 1 | 2 | 1 | 1 | 1 |
| *Sphenosuchus acutus* | ? | ? | ? | ? | ? | 1 | 0 | 0 | 0 | 1 | 1 | 3 | 1 | 1 | 1 |
| *Terrestrisuchus gracilis* | 0 | ? | 0 | 0 | 0 | 1 | 1 | 0 | 0 | 1 | 1 | 2 | 1 | 1 | 1 |
| *Dibothrosuchus elaphros* | ? | ? | ? | ? | ? | ? | ? | 0 | 0 | 1 | 1 | 3 | 1 | 1 | 1 |
| *Litargosuchus leptorhynchus* | 0 | ? | 0 | 0 | 0 | 1 | 1 | 0 | 0 | 1 | ? | ? | 0 | 1 | 1 |
| *Kayentasuchus walker* | ? | ? | ? | ? | ? | ? | ? | ? | ? | ? | ? | ? | ? | ? | ? |
| *Junggarsuchus sloani* | - | - | - | - | ? | 1 | ? | ? | ? | ? | 1 | ? | ? | ? | ? |
| *Orthosuchus stormbergi* | ? | ? | 0 | 0 | 0 | 1 | 0 | 0 | 0 | 1 | 1 | 4 | 0 | 1 | 0 |
| *Protosuchus richardsoni* | 1 | 1 | 0 | 0 | 0 | 1 | 1 | 1 | 0 | 1 | 1 | 4 | 0 | 1 | 0 |
| *Protosuchus haughtoni* | 1 | ? | 0 | 0 | ? | ? | ? | ? | ? | ? | 1 | ? | ? | ? | ? |
| *Alligator mississippiensis* | 1 | ? | 0 | 0 | 0 | 1 | 0 | 0 | 1 | 1 | 0 | 4 | 0 | 1 | 0 |
|  |  |  |  |  |  |  |  |  |  |  |  |  |  |  |  |
|  |  |  |  |  |  |  |  |  |  |  |  |  |  |  |  |
|  |  |  |  |  |  |  |  |  |  |  |  |  |  |  |  |
|  | 166 | 167 | 168 | 169 | 170 | 171 | 172 | 173 | 174 | 175 | 176 | 177 | 178 | 179 | 180 |
| *Euparkeri capensis* | 0 | 0 | 0 | 0 | 0 | 0 | ? | 0 | 0 | 0 | ? | 0 | 0 | 0 | 0 |
| *Machaeroprosopus pristinus* | 0 | 0 | 0 | 0 | 0 | 0 | ? | 0 | 0 | 0 | ? | 0 | ? | ? | ? |
| *Riojasuchus tenulsceps* | 0 | 0 | 0 | 0 | 1 | 0 | 0 | 0 | 1 | 0 | ? | 0 | ? | 0 | 0 |
| *Revueltosaurus callenderi* | 0 | 0 | 0 | 0 | 0 | 0 | 0 | 0 | 1 | 0 | ? | 1 | 1 | 0 | 0 |
| *Stagonolepis robertsoni* | 0 | 0 | 0 | 0 | 0 | 0 | 0 | 0 | ? | ? | ? | ? | ? | 0 | 0 |
| *Turfanosuchus dabanensis* | ? | ? | ? | ? | ? | 0 | ? | ? | ? | ? | ? | ? | ? | ? | ? |
| *Gracilisuchus stipanicicorum* | ? | ? | ? | ? | ? | 0 | ? | ? | ? | ? | ? | ? | ? | ? | ? |
| *Ticinosuchus ferox* | ? | ? | 0 | 0 | ? | 0 | ? | 0 | 1 | ? | ? | ? | 0 | 0 | 0 |
| *Qianosuchus mixtus* | ? | 0 | 0 | 0 | ? | 0 | ? | 0 | ? | ? | 0 | ? | ? | ? | ? |
| *Arizonasaurus babbitti* | 0 | 0 | ? | ? | ? | ? | ? | ? | ? | ? | ? | ? | ? | ? | ? |
| *Xilousuchus sapingensis* | ? | ? | ? | ? | ? | ? | ? | ? | ? | ? | ? | ? | ? | ? | ? |
| *Poposaurus gracilis* | 0 | 0 | 0 | 0 | 0 | ? | ? | 0 | 1 | 0 | ? | 0 | 0 | 0 | 0 |
| *Lotosaurus adentus* | 0 | 0 | 0 | 0 | 0 | ? | ? | 0 | 1 | 0 | ? | ? | 0 | ? | 0 |
| *Sillosuchus longicervix* | 0 | 0 | ? | ? | ? | ? | ? | 1 | ? | ? | ? | ? | ? | ? | ? |
| *Shuvosaurus inexpectatus* | 1 | 0 | 0 | 0 | 1 | 0 | 1 | 1 | 1 | 0 | ? | ? | ? | ? | ? |
| *Effigia okeeffeae* | 1 | 0 | 0 | ? | 1 | 0 | ? | 1 | ? | ? | 0 | 0 | 0 | 0 | 0 |
| *Prestosuchus* | 0 | 1 | 0 | 0 | ? | ? | ? | 0 | ? | ? | ? | ? | ? | ? | ? |
| *Saurosuchus galilei* | ? | ? | ? | ? | ? | ? | ? | ? | ? | ? | ? | ? | ? | ? | ? |
| *Batrachotomus kuperferzellensis* | 0 | 0 | 0 | 0 | 0 | 0 | 0 | 0 | 1 | 1 | 0 | 1 | 0 | ? | ? |
| *Decuriasuchus quartacolonia* | ? | ? | ? | ? | ? | ? | ? | ? | ? | ? | ? | ? | ? | ? | ? |
| *Fasolasuchus tenax* | ? | ? | ? | ? | ? | ? | ? | ? | 1 | 1 | ? | 0 | ? | ? | ? |
| *Rauisuchus triradentes* | ? | 0 | ? | ? | ? | ? | ? | ? | ? | ? | ? | ? | ? | ? | ? |
| *Postosuchus alisonae* | 0 | 0 | 1 | 0 | 0 | 0 | 0 | 0 | 1 | 1 | 0 | 1 | ? | 0 | 0 |
| *Postosuchus kirkpatricki* | 0 | 0 | 1 | 0 | 0 | 0 | 0 | 0 | 1 | 1 | 0 | 1 | 0 | ? | 0 |
| *Polonosuchus silesiacus* | ? | ? | ? | ? | ? | ? | ? | ? | ? | ? | ? | ? | ? | ? | ? |
| *Carnufex carolinensis* | ? | ? | ? | ? | 0 | 0 | 0 | 0 | ? | ? | ? | ? | ? | ? | ? |
| CM 73372 | ? | ? | ? | ? | ? | ? | ? | 0 | ? | ? | ? | ? | ? | 0 | 0 |
| *Redondavenator quayensis* | ? | ? | ? | ? | ? | ? | ? | ? | ? | ? | ? | ? | ? | ? | ? |
| *“Hesperosuchus agilis”* | ? | ? | 1 | ? | 1 | 1 | 1 | 0 | 1 | 1 | 1 | 1 | 1 | 1 | 0 |
| *Dromicosuchus grallator* | 0 | 0 | 1 | 1 | 1 | 1 | 1 | 0 | 0 | 1 | ? | ? | 0 | 1 | 0 |
| *Hesperosuchus agilis* HT | 0 | 0 | 1 | 1 | 1 | 1 | 1 | 0 | 1 | 1 | 0 | 1 | 0 | 1 | ? |
| *Sphenosuchus acutus* | 0 | 0 | 1 | ? | 1 | 1 | 1 | 0 | ? | ? | ? | ? | ? | ? | ? |
| *Terrestrisuchus gracilis* | 0 | 0 | 1 | 0 | 1 | 1 | 1 | 0 | 1 | 1 | 0 | 1 | 0 | 1 | 0 |
| *Dibothrosuchus elaphros* | 0 | 0 | 1 | 0 | 1 | 1 | 1 | 0 | 1 | 1 | 0 | 1 | 0 | 1 | 1 |
| *Litargosuchus leptorhynchus* | 0 | 0 | 1 | 0 | 1 | ? | ? | 0 | ? | ? | ? | 1 | 0 | 1 | ? |
| *Kayentasuchus walker* | ? | ? | ? | ? | ? | ? | ? | ? | ? | ? | ? | ? | ? | ? | ? |
| *Junggarsuchus sloani* | ? | ? | ? | ? | ? | ? | ? | ? | ? | ? | ? | ? | ? | 1 | ? |
| *Orthosuchus stormbergi* | 0 | 0 | 1 | 0 | 1 | ? | ? | 0 | 1 | 1 | ? | 1 | 0 | 1 | 1 |
| *Protosuchus richardsoni* | 0 | 0 | ? | 0 | 1 | ? | ? | 0 | 1 | 1 | 0 | 1 | 1 | 1 | 1 |
| *Protosuchus haughtoni* | ? | ? | ? | ? | ? | ? | ? | ? | ? | ? | ? | ? | ? | ? | ? |
| *Alligator mississippiensis* | 0 | 0 | 0 | 1 | 1 | 1 | 1 | 0 | 1 | 1 | 0 | 1 | 1 | 1 | 0 |
|  |  |  |  |  |  |  |  |  |  |  |  |  |  |  |  |
|  |  |  |  |  |  |  |  |  |  |  |  |  |  |  |  |
|  | 181 | 182 | 183 | 184 | 185 | 186 | 187 | 188 | 189 | 190 | 191 | 192 | 193 | 194 | 195 |
| *Euparkeri capensis* | 0 | ? | 0 | 0 | - | 0 | 0 | 0 | 0 | 0 | 0 | 0 | 0 | 0 | 0 |
| *Machaeroprosopus pristinus* | 0 | ? | 0 | 0 | - | 0 | 0 | 0 | 0 | 0 | 0 | 0 | 0 | 0 | 0 |
| *Riojasuchus tenulsceps* | 0 | ? | 0 | 0 | - | 0 | 0 | 0 | 0 | 0 | 0 | 0 | 1 | 0 | ? |
| *Revueltosaurus callenderi* | 0 | ? | 0 | 0 | - | 0 | 0 | 0 | 0 | 0 | 0 | 0 | 0 | 0 | 0 |
| *Stagonolepis robertsoni* | 0 | ? | 0 | 0 | - | 0 | 1 | 0 | 0 | 0 | 0 | 0 | 0 | 0 | 0 |
| *Turfanosuchus dabanensis* | ? | ? | 0 | 0 | - | 0 | 0 | 0 | 0 | 0 | 0 | 0 | 0 | 0 | 1 |
| *Gracilisuchus stipanicicorum* | ? | ? | 0 | 0 | - | 0 | 0 | 0 | 0 | 0 | 0 | 0 | 0 | 0 | 1 |
| *Ticinosuchus ferox* | 0 | ? | ? | ? | ? | 0 | ? | ? | 0 | ? | 0 | 0 | 0 | ? | 0 |
| *Qianosuchus mixtus* | ? | ? | 0 | 1 | ? | 0 | ? | 0 | 1 | 0 | 0 | 0 | 0 | 0 | 0 |
| *Arizonasaurus babbitti* | ? | ? | 0 | 1 | 0 | 0 | 1 | 0 | 1 | 0 | 0 | 0 | ? | 0 | 1 |
| *Xilousuchus sapingensis* | ? | ? | ? | ? | ? | ? | ? | ? | ? | ? | ? | ? | ? | ? | ? |
| *Poposaurus gracilis* | 0 | ? | 1 | 2 | 2 | 1 | 0 | 1 | 1 | ? | 0 | 0 | 1 | 0 | 1 |
| *Lotosaurus adentus* | 0 | ? | 1 | 2 | 0 | ? | 0 | 0 | 1 | ? | 0 | 1 | ? | ? | ? |
| *Sillosuchus longicervix* | ? | ? | 1 | 2 | 2 | ? | 0 | 1 | 1 | 1 | ? | 1 | 1 | 0 | 1 |
| *Shuvosaurus inexpectatus* | ? | ? | 1 | 2 | 2 | 2 | 0 | 0 | 1 | 1 | 0 | 1 | 1 | 0 | 1 |
| *Effigia okeeffeae* | 0 | ? | 1 | 2 | 2 | 2 | 0 | 0 | 1 | 1 | 0 | 1 | ? | 0 | 1 |
| *Prestosuchus* | ? | ? | 0 | 0 | ? | 0 | 1 | 0 | 0 | 0 | 0 | 0 | 1 | 0 | 1 |
| *Saurosuchus galilei* | ? | ? | 0 | 1 | 0 | 0 | 1 | 0 | 0 | 0 | 0 | 0 | 1 | 0 | ? |
| *Batrachotomus kuperferzellensis* | ? | ? | 0 | 1 | 0 | 0 | 1 | 0 | 0 | 0 | 0 | 0 | 1 | 0 | 1 |
| *Decuriasuchus quartacolonia* | ? | ? | ? | ? | ? | ? | ? | ? | ? | ? | ? | ? | ? | ? | ? |
| *Fasolasuchus tenax* | ? | ? | ? | ? | ? | ? | ? | ? | ? | ? | ? | ? | ? | 1 | ? |
| *Rauisuchus triradentes* | ? | ? | ? | 1 | 0 | 0 | ? | 0 | 0 | 0 | 0 | 0 | ? | ? | ? |
| *Postosuchus alisonae* | 0 | 0 | ? | ? | ? | ? | ? | ? | ? | ? | ? | ? | ? | ? | ? |
| *Postosuchus kirkpatricki* | 0 | ? | 0 | 1 | 0 | ? | 1 | 0 | 0 | 0 | 0 | 0 | ? | ? | ? |
| *Polonosuchus silesiacus* | ? | ? | ? | ? | ? | ? | ? | ? | ? | ? | ? | ? | ? | ? | ? |
| *Carnufex carolinensis* | ? | ? | ? | ? | ? | ? | ? | ? | ? | ? | ? | ? | ? | ? | ? |
| CM 73372 | 0 | ? | 0 | 1 | 0 | 1 | ? | 0 | 1 | ? | 0 | 0 | 1 | ? | 1 |
| *Redondavenator quayensis* | ? | ? | ? | ? | ? | ? | ? | ? | ? | ? | ? | ? | ? | ? | ? |
| *“Hesperosuchus agilis”* | 0 | 1 | ? | ? | ? | ? | ? | ? | ? | ? | ? | ? | 1 | 1 | ? |
| *Dromicosuchus grallator* | 0 | 1 | 0 | 1 | 1 | 1 | 1 | 0 | 0 | 0 | 1 | 0 | ? | ? | ? |
| *Hesperosuchus agilis* HT | 0 | ? | 0 | 1 | 1 | 1 | 1 | 0 | 0 | 1 | 1 | 0 | ? | ? | ? |
| *Sphenosuchus acutus* | ? | ? | ? | ? | ? | ? | ? | ? | ? | ? | ? | ? | ? | ? | ? |
| *Terrestrisuchus gracilis* | 0 | 1 | 0 | 1 | ? | 1 | 0 | 0 | 1 | 1 | 1 | 0 | 1 | 1 | 1 |
| *Dibothrosuchus elaphros* | 0 | 1 | 0 | 0 | - | 1 | 0 | 0 | 1 | 1 | 1 | 0 | ? | ? | ? |
| *Litargosuchus leptorhynchus* | ? | ? | ? | ? | ? | 1 | ? | ? | ? | ? | ? | ? | ? | ? | ? |
| *Kayentasuchus walker* | ? | ? | 0 | ? | ? | ? | 0 | ? | 1 | 1 | ? | ? | ? | ? | ? |
| *Junggarsuchus sloani* | ? | ? | ? | ? | ? | ? | ? | ? | ? | ? | ? | ? | ? | ? | ? |
| *Orthosuchus stormbergi* | 1 | ? | 0 | 1 | ? | 1 | 0 | 0 | 1 | 0 | 1 | 0 | 0 | 2 | 1 |
| *Protosuchus richardsoni* | 0 | 1 | 0 | 1 | ? | 1 | 0 | 0 | 1 | 1 | 1 | 0 | 0 | ? | 1 |
| *Protosuchus haughtoni* | ? | ? | ? | ? | ? | ? | ? | ? | ? | ? | ? | ? | ? | ? | ? |
| *Alligator mississippiensis* | 0 | ? | ? | 0 | - | ? | 0 | 0 | 1 | 1 | 0 | 0 | 0 | 2 | 0 |
|  |  |  |  |  |  |  |  |  |  |  |  |  |  |  |  |
|  |  |  |  |  |  |  |  |  |  |  |  |  |  |  |  |
|  |  |  |  |  |  |  |  |  |  |  |  |  |  |  |  |
|  | 196 | 197 | 198 | 199 | 200 | 201 | 202 | 203 | 204 | 205 | 206 | 207 | 208 | 209 | 210 |
| *Euparkeri capensis* | 0 | ? | 0 | 0 | 0 | 0 | 0 | 0 | 0 | 0 | 0 | 0 | 0 | 0 | 0 |
| *Machaeroprosopus pristinus* | 0 | ? | 0 | 0 | 0 | 0 | 0 | 0 | 0 | 0 | 0 | 0 | 0 | 0 | 0 |
| *Riojasuchus tenulsceps* | 0 | ? | ? | 0 | 0 | 0 | ? | ? | ? | 0 | 0 | ? | 0 | 1 | 0 |
| *Revueltosaurus callenderi* | 0 | ? | 0 | 0 | 0 | 0 | 0 | 0 | 0 | 0 | 0 | 0 | 0 | 1 | 0 |
| *Stagonolepis robertsoni* | ? | ? | 0 | ? | 0 | 0 | 0 | 0 | 0 | 0 | 0 | 0 | 0 | 1 | 0 |
| *Turfanosuchus dabanensis* | 0 | ? | 0 | ? | 0 | 0 | 0 | 0 | 0 | 0 | 0 | 0 | ? | 1 | 0 |
| *Gracilisuchus stipanicicorum* | 0 | ? | 2 | 0 | 0 | 0 | 0 | 0 | 0 | 0 | ? | 0 | 0 | 0 | 0 |
| *Ticinosuchus ferox* | 0 | ? | ? | ? | 0 | ? | 1 | 1 | 1 | 0 | ? | 1 | 0 | 1 | 0 |
| *Qianosuchus mixtus* | 1 | 0 | 1 | 1 | 0 | 0 | 1 | 0 | 1 | 0 | 0 | 1 | 0 | ? | ? |
| *Arizonasaurus babbitti* | 2 | 0 | 2 | 1 | 1 | 0 | 1 | 1 | 1 | 0 | 0 | 1 | ? | 1 | 1 |
| *Xilousuchus sapingensis* | ? | ? | ? | ? | ? | ? | ? | ? | ? | ? | ? | ? | ? | ? | ? |
| *Poposaurus gracilis* | 2 | 0 | 2 | 1 | 1 | 0 | 1 | 1 | 1 | 0 | 0 | ? | 0 | 1 | 1 |
| *Lotosaurus adentus* | ? | ? | 1 | ? | ? | ? | 2 | 1 | 0 | 0 | 0 | ? | 0 | ? | ? |
| *Sillosuchus longicervix* | 2 | 0 | 2 | ? | 1 | 0 | 2 | 1 | ? | 0 | 0 | ? | ? | 1 | 1 |
| *Shuvosaurus inexpectatus* | 2 | 1 | 2 | 1 | 2 | 1 | 2 | 1 | 0 | 0 | 0 | ? | 0 | 2 | 1 |
| *Effigia okeeffeae* | 2 | 1 | 2 | 1 | 2 | 1 | 2 | 1 | 0 | 0 | 0 | ? | ? | 2 | 1 |
| *Prestosuchus* | 1 | 0 | 1 | 0 | 1 | 0 | 1 | 1 | 1 | 1 | 0 | 1 | 0 | 1 | 0 |
| *Saurosuchus galilei* | 1 | 0 | 1 | 0 | 0 | 0 | 1 | 1 | 1 | 0 | 0 | ? | ? | 1 | ? |
| *Batrachotomus kuperferzellensis* | 1 | 0 | 1 | 0 | 0 | 0 | 1 | 0 | 1 | 0 | 0 | 1 | 0 | 1 | 1 |
| *Decuriasuchus quartacolonia* | ? | ? | ? | ? | ? | ? | ? | ? | ? | ? | ? | ? | ? | ? | ? |
| *Fasolasuchus tenax* | ? | ? | 1 | 0 | ? | 0 | 1 | ? | ? | 0 | 0 | ? | 0 | 1 | 1 |
| *Rauisuchus triradentes* | ? | ? | ? | ? | ? | ? | ? | ? | ? | ? | ? | ? | ? | ? | ? |
| *Postosuchus alisonae* | 1 | 0 | ? | ? | ? | ? | ? | ? | ? | ? | ? | ? | ? | ? | ? |
| *Postosuchus kirkpatricki* | ? | ? | 1 | ? | ? | ? | 1 | 1 | 1 | 0 | 0 | ? | 0 | 1 | 1 |
| *Polonosuchus silesiacus* | ? | ? | ? | ? | ? | ? | ? | ? | ? | ? | ? | ? | ? | ? | ? |
| *Carnufex carolinensis* | ? | ? | ? | ? | ? | ? | ? | ? | ? | ? | ? | ? | ? | ? | ? |
| CM 73372 | 1 | 0 | ? | ? | 1 | 0 | 1 | 1 | 0 | 0 | ? | 1 | 0 | 1 | 1 |
| *Redondavenator quayensis* | ? | ? | ? | ? | ? | ? | ? | ? | ? | ? | ? | ? | ? | ? | ? |
| *“Hesperosuchus agilis”* | 1 | 0 | 1 | 0 | 1 | 0 | ? | ? | ? | ? | ? | ? | 0 | 1 | 1 |
| *Dromicosuchus grallator* | ? | ? | 1 | ? | ? | ? | ? | ? | ? | ? | ? | ? | 0 | 1 | 1 |
| *Hesperosuchus agilis* HT | 1 | 0 | ? | ? | 1 | 0 | ? | ? | ? | ? | ? | ? | 0 | 1 | 1 |
| *Sphenosuchus acutus* | ? | ? | ? | ? | ? | ? | ? | ? | ? | ? | ? | ? | ? | ? | ? |
| *Terrestrisuchus gracilis* | 0 | ? | 1 | 0 | 0 | 0 | 1 | 0 | 0 | 0 | 1 | 0 | 0 | 1 | 1 |
| *Dibothrosuchus elaphros* | ? | ? | ? | ? | ? | ? | ? | ? | ? | ? | ? | ? | ? | ? | ? |
| *Litargosuchus leptorhynchus* | ? | ? | ? | ? | ? | ? | 0 | 0 | 0 | ? | ? | ? | 1 | ? | ? |
| *Kayentasuchus walker* | ? | ? | ? | ? | ? | ? | ? | ? | ? | ? | ? | ? | ? | 1 | 1 |
| *Junggarsuchus sloani* | ? | ? | ? | ? | ? | ? | ? | ? | ? | ? | ? | ? | ? | ? | ? |
| *Orthosuchus stormbergi* | 0 | ? | 1 | ? | - | 0 | 0 | 0 | 0 | 0 | 1 | 0 | 0 | 1 | 1 |
| *Protosuchus richardsoni* | 0 | ? | 1 | 0 | - | 0 | 0 | 0 | 0 | 0 | 1 | 0 | 0 | 1 | 1 |
| *Protosuchus haughtoni* | ? | ? | ? | ? | ? | ? | ? | ? | ? | ? | ? | ? | ? | ? | ? |
| *Alligator mississippiensis* | 0 | ? | 1 | ? | - | - | 0 | 0 | 0 | 0 | 1 | 1 | 0 | 1 | 1 |
|  |  |  |  |  |  |  |  |  |  |  |  |  |  |  |  |
|  |  |  |  |  |  |  |  |  |  |  |  |  |  |  |  |
|  |  |  |  |  |  |  |  |  |  |  |  |  |  |  |  |
|  | 211 | 212 | 213 | 214 | 215 | 216 | 217 | 218 | 219 | 220 | 221 | 222 | 223 | 224 | 225 |
| *Euparkeri capensis* | 0 | 0 | 0 | 0 | 0 | 0 | 0 | 0 | 0 | 0 | 0 | 0 | 0 | 0 | 0 |
| *Machaeroprosopus pristinus* | 0 | 1 | 0 | 0 | 0 | 0 | 0 | 0 | 0 | 0 | 0 | 0 | 0 | 0 | 1 |
| *Riojasuchus tenulsceps* | 0 | 0 | 0 | 1 | 0 | 0 | 0 | 0 | 0 | 0 | 1 | 0 | 0 | ? | 1 |
| *Revueltosaurus callenderi* | 0 | 0 | 0 | 0 | 0 | 0 | 0 | 0 | 0 | 0 | 0 | 0 | 0 | 0 | 0 |
| *Stagonolepis robertsoni* | 0 | ? | 0 | 0 | 0 | 0 | 0 | 0 | 0 | 0 | ? | 0 | 0 | ? | 1 |
| *Turfanosuchus dabanensis* | 0 | 0 | 0 | 0 | 0 | 0 | 0 | 0 | 0 | 0 | 0 | 0 | 0 | ? | ? |
| *Gracilisuchus stipanicicorum* | 0 | 0 | 0 | 0 | 0 | 0 | ? | 0 | 0 | ? | 1 | 0 | 0 | ? | 0 |
| *Ticinosuchus ferox* | ? | ? | ? | ? | ? | ? | 0 | ? | 0 | ? | ? | ? | ? | ? | 0 |
| *Qianosuchus mixtus* | 0 | ? | 0 | 0 | 0 | 0 | 0 | ? | 0 | ? | ? | ? | ? | ? | 0 |
| *Arizonasaurus babbitti* | 0 | 0 | 0 | 0 | 0 | 0 | 0 | 1 | 0 | 1 | 0 | 0 | 0 | 1 | ? |
| *Xilousuchus sapingensis* | ? | ? | ? | ? | ? | ? | ? | ? | ? | ? | ? | ? | ? | ? | ? |
| *Poposaurus gracilis* | 0 | 0 | 0 | 0 | 0 | 0 | 0 | 1 | 0 | ? | ? | 0 | 0 | 1 | 0 |
| *Lotosaurus adentus* | ? | ? | 0 | 0 | 0 | 0 | 0 | 1 | 0 | 1 | 1 | ? | ? | ? | 0 |
| *Sillosuchus longicervix* | ? | ? | 0 | 0 | 0 | ? | 0 | ? | 0 | ? | ? | 0 | ? | ? | ? |
| *Shuvosaurus inexpectatus* | 1 | 0 | 0 | 0 | 0 | 0 | 1 | 1 | 1 | 1 | 1 | 0 | 1 | 1 | 0 |
| *Effigia okeeffeae* | 1 | ? | 0 | 0 | 0 | 0 | 1 | 1 | ? | 1 | 1 | 0 | 1 | 1 | 0 |
| *Prestosuchus* | 0 | 0 | 0 | 0 | 0 | 0 | 0 | 1 | 0 | 0 | 0 | 0 | 0 | 0 | 1 |
| *Saurosuchus galilei* | 0 | 0 | 0 | 0 | 0 | ? | 0 | 1 | 0 | 0 | 1 | 0 | 0 | 0 | 1 |
| *Batrachotomus kuperferzellensis* | 0 | 0 | 0 | 0 | 0 | 0 | 0 | 1 | 0 | 1 | 1 | 0 | 0 | 0 | 0 |
| *Decuriasuchus quartacolonia* | ? | ? | ? | ? | ? | ? | ? | ? | ? | ? | ? | ? | ? | ? | ? |
| *Fasolasuchus tenax* | 0 | ? | 0 | 0 | 0 | 1 | 0 | 0 | 0 | 1 | 1 | 0 | 0 | ? | 0 |
| *Rauisuchus triradentes* | ? | ? | ? | ? | ? | ? | ? | ? | ? | ? | 1 | ? | ? | ? | 0 |
| *Postosuchus alisonae* | ? | ? | ? | ? | ? | ? | ? | ? | ? | ? | ? | ? | ? | ? | 0 |
| *Postosuchus kirkpatricki* | 0 | 0 | 0 | 0 | 0 | ? | 0 | 0 | 0 | 1 | 1 | 0 | 0 | ? | 0 |
| *Polonosuchus silesiacus* | ? | ? | ? | ? | ? | ? | ? | ? | ? | ? | ? | ? | ? | ? | ? |
| *Carnufex carolinensis* | ? | ? | ? | ? | ? | ? | ? | ? | ? | ? | ? | ? | ? | ? | ? |
| CM 73372 | ? | ? | 0 | 0 | 0 | 1 | 0 | 0 | ? | ? | ? | ? | ? | 0 | 0 |
| *Redondavenator quayensis* | ? | ? | ? | ? | ? | ? | ? | ? | ? | ? | ? | ? | ? | ? | ? |
| *“Hesperosuchus agilis”* | 0 | 0 | 0 | 0 | 0 | 1 | 0 | 0 | ? | 1 | 1 | ? | ? | 0 | 0 |
| *Dromicosuchus grallator* | 0 | 0 | 0 | 0 | 1 | 1 | 0 | 0 | 0 | 1 | 1 | 0 | 0 | ? | 0 |
| *Hesperosuchus agilis* HT | 0 | 0 | 0 | 0 | 0 | 1 | 0 | 0 | 0 | ? | ? | 1 | ? | ? | ? |
| *Sphenosuchus acutus* | ? | ? | ? | ? | ? | ? | ? | ? | ? | ? | ? | ? | ? | ? | ? |
| *Terrestrisuchus gracilis* | 0 | 0 | 1 | 0 | 0 | 1 | 0 | 0 | 0 | 0 | 1 | 0 | 0 | 1 | 0 |
| *Dibothrosuchus elaphros* | ? | ? | ? | ? | ? | ? | ? | ? | ? | ? | ? | ? | ? | ? | ? |
| *Litargosuchus leptorhynchus* | ? | ? | ? | ? | ? | ? | ? | ? | ? | ? | ? | 1 | ? | ? | 0 |
| *Kayentasuchus walker* | ? | 0 | 1 | 1 | 0 | 1 | 1 | 0 | 0 | ? | ? | 1 | ? | ? | ? |
| *Junggarsuchus sloani* | ? | ? | ? | ? | ? | ? | ? | ? | ? | ? | ? | ? | ? | ? | ? |
| *Orthosuchus stormbergi* | 0 | 1 | 0 | 0 | 0 | 1 | 0 | 0 | 0 | 0 | 1 | 1 | 0 | ? | ? |
| *Protosuchus richardsoni* | ? | 0 | 0 | 0 | 0 | 1 | 0 | 0 | 0 | 1 | 1 | 1 | 0 | 0 | 0 |
| *Protosuchus haughtoni* | ? | ? | ? | ? | ? | ? | ? | ? | ? | ? | ? | ? | ? | ? | ? |
| *Alligator mississippiensis* | 0 | 0 | ? | 0 | 1 | 1 | 0 | 0 | 0 | 0 | 1 | 1 | 0 | 0 | 0 |
|  |  |  |  |  |  |  |  |  |  |  |  |  |  |  |  |
|  |  |  |  |  |  |  |  |  |  |  |  |  |  |  |  |
|  |  |  |  |  |  |  |  |  |  |  |  |  |  |  |  |
|  | 226 | 227 | 228 | 229 | 230 | 231 | 232 | 233 | 234 | 235 | 246 | 237 | 238 | 239 | 240 |
| *Euparkeri capensis* | 0 | 0 | 0 | 0 | 0 | 0 | 0 | 0 | 0 | 0 | 0 | 0 | 0 | 0 | 0 |
| *Machaeroprosopus pristinus* | 1 | 0 | 0 | 0 | 0 | 1 | 0 | 0 | 0 | 0 | 0 | 1 | 0 | 0 | 0 |
| *Riojasuchus tenulsceps* | 1 | 0 | 0 | 0 | 0 | 0 | 1 | 0 | 0 | 0 | 0 | 1 | 0 | 0 | 0 |
| *Revueltosaurus callenderi* | 0 | 0 | 0 | 0 | 0 | 0 | 1 | 0 | 0 | 0 | 0 | 1 | 1 | 0 | 1 |
| *Stagonolepis robertsoni* | 1 | 0 | 0 | 0 | ? | ? | ? | ? | ? | ? | ? | ? | 1 | ? | ? |
| *Turfanosuchus dabanensis* | ? | 1 | ? | ? | ? | ? | ? | 0 | 0 | 0 | ? | ½ | 0 | 1 | 1 |
| *Gracilisuchus stipanicicorum* | 0 | ? | 0 | 0 | 0 | 1 | 1 | 0 | 0 | 0 | 1 | 2 | 0 | 1 | 1 |
| *Ticinosuchus ferox* | 0 | ? | 0 | 0 | ? | ? | ? | 0 | 0 | 0 | ? | 1 | 1 | 0 | 1 |
| *Qianosuchus mixtus* | 0 | ? | 0 | 1 | ? | ? | ? | ? | 0 | ? | ? | 1 | 1 | 0 | 1 |
| *Arizonasaurus babbitti* | ? | ? | ? | ? | ? | ? | ? | ? | ? | ? | ? | ? | ? | ? | ? |
| *Xilousuchus sapingensis* | ? | ? | ? | ? | ? | ? | ? | ? | ? | ? | ? | ? | ? | ? | ? |
| *Poposaurus gracilis* | 0 | 1 | 0 | ? | 1 | 1 | ? | 0 | 1 | 1 | 0 | 1 | 1 | 1 | 0 |
| *Lotosaurus adentus* | 0 | 0 | 0 | 1 | ? | ? | ? | 0 | 0 | 0 | ? | 1 | 1 | 0 | 0 |
| *Sillosuchus longicervix* | ? | ? | ? | ? | ? | ? | ? | ? | ? | ? | ? | ? | ? | ? | ? |
| *Shuvosaurus inexpectatus* | 0 | 1 | 0 | 1 | 1 | 1 | 1 | 0 | 1 | 0 | 0 | 1 | 1 | 0 | 0 |
| *Effigia okeeffeae* | 0 | 1 | 0 | 1 | 1 | 1 | 1 | 0 | ? | 0 | 0 | 1 | 1 | ? | 0 |
| *Prestosuchus* | 1 | 0 | 0 | 0 | 0 | 0 | ? | 0 | 0 | 0 | ? | 2 | 1 | 0 | 1 |
| *Saurosuchus galilei* | 1 | 0 | 0 | 0 | 0 | ? | ? | 0 | 1 | 0 | ? | 2 | 1 | ? | 0 |
| *Batrachotomus kuperferzellensis* | 1 | 0 | 0 | 0 | 0 | ? | ? | ? | 1 | ? | ? | ? | 1 | 0 | 1 |
| *Decuriasuchus quartacolonia* | ? | ? | ? | ? | ? | ? | ? | ? | ? | ? | ? | ? | ? | ? | ? |
| *Fasolasuchus tenax* | 1 | 0 | 1 | 0 | 1 | 1 | 1 | 1 | 1 | 1 | 1 | 2 | 1 | 1 | 0 |
| *Rauisuchus triradentes* | ? | ? | ? | ? | ? | ? | ? | ? | ? | ? | 1 | ? | ? | ? | ? |
| *Postosuchus alisonae* | 1 | 1 | 1 | 0 | 1 | 1 | 1 | 1 | 1 | 1 | 1 | 2 | 1 | 1 | 0 |
| *Postosuchus kirkpatricki* | 1 | 1 | 1 | 0 | ? | ? | ? | 1 | 1 | 1 | 1 | 2 | 1 | 1 | 0 |
| *Polonosuchus silesiacus* | ? | ? | ? | ? | ? | ? | ? | ? | ? | ? | ? | ? | ? | ? | ? |
| *Carnufex carolinensis* | ? | ? | ? | ? | ? | ? | ? | ? | ? | ? | ? | ? | ? | ? | ? |
| CM 73372 | 1 | 1 | 1 | 0 | 1 | 1 | 1 | 1 | 1 | 1 | 1 | 2 | 1 | 1 | 0 |
| *Redondavenator quayensis* | ? | ? | ? | ? | ? | ? | ? | ? | ? | ? | ? | ? | ? | ? | ? |
| *“Hesperosuchus agilis”* | 0 | 1 | 1 | 0 | 1 | 1 | 1 | 1 | 1 | 1 | 1 | 2 | 1 | 1 | 0 |
| *Dromicosuchus grallator* | 0 | 1 | 1 | 0 | ? | ? | ? | ? | ? | ? | ? | ? | 1 | ? | ? |
| *Hesperosuchus agilis* HT | ? | 1 | 1 | 0 | 1 | ? | 1 | ? | 1 | ? | ? | ? | 1 | ? | ? |
| *Sphenosuchus acutus* | ? | ? | ? | ? | ? | ? | ? | ? | ? | ? | ? | ? | ? | ? | ? |
| *Terrestrisuchus gracilis* | 0 | 1 | 1 | 1 | 1 | 1 | 1 | 1 | 1 | 1 | 1 | 2 | 1 | 1 | 0 |
| *Dibothrosuchus elaphros* | ? | ? | ? | ? | ? | ? | ? | ? | ? | ? | ? | ? | ? | ? | ? |
| *Litargosuchus leptorhynchus* | 0 | 1 | ? | 1 | ? | ? | ? | ? | ? | ? | ? | ? | ? | ? | 0 |
| *Kayentasuchus walker* | ? | ? | ? | ? | ? | ? | ? | ? | ? | ? | ? | ? | ? | ? | ? |
| *Junggarsuchus sloani* | ? | ? | ? | ? | ? | ? | ? | ? | ? | ? | ? | ? | ? | ? | ? |
| *Orthosuchus stormbergi* | ? | ? | 1 | 1 | 1 | ? | ? | 1 | 1 | ? | 1 | 2 | 1 | 1 | 0 |
| *Protosuchus richardsoni* | 0 | 1 | 1 | 1 | 1 | ? | 0 | 1 | 1 | 1 | 1 | 2 | 1 | 1 | 0 |
| *Protosuchus haughtoni* | ? | ? | ? | ? | ? | ? | ? | ? | ? | ? | ? | ? | ? | ? | ? |
| *Alligator mississippiensis* | 0 | 1 | 1 | 1 | 1 | 1 | 1 | 1 | 1 | 1 | 1 | 1 | 1 | 1 | 0 |
|  |  |  |  |  |  |  |  |  |  |  |  |  |  |  |  |
|  |  |  |  |  |  |  |  |  |  |  |  |  |  |  |  |
|  |  |  |  |  |  |  |  |  |  |  |  |  |  |  |  |
|  | 241 | 242 | 243 | 244 | 245 | 246 | 247 | 248 | 249 | 250 | 251 |  |  |  |  |
| *Euparkeri capensis* | 0 | 0 | 0 | 0 | 0 | 0 | ? | 0 | 0 | 0 | 0 |  |  |  |  |
| *Machaeroprosopus pristinus* | 1 | 0 | 0 | 0 | 0 | 0 | ? | 0 | 0 | 0 | 2 |  |  |  |  |
| *Riojasuchus tenulsceps* | 0 | 0 | 0 | 0 | 0 | 0 | ? | 0 | 0 | 0 | 2 |  |  |  |  |
| *Revueltosaurus callenderi* | 1 | 0 | 0 | ? | 0 | 0 | ? | 0 | 0 | ? | ? |  |  |  |  |
| *Stagonolepis robertsoni* | ? | 0 | 0 | 0 | ? | ? | 0 | 0 | 0 | 0 | ? |  |  |  |  |
| *Turfanosuchus dabanensis* | 1 | ? | ? | ? | ? | ? | ? | 0 | 0 | ? | ? |  |  |  |  |
| *Gracilisuchus stipanicicorum* | 1 | 0 | 0 | 0 | 0 | 0 | ? | 0 | 0 | 0 | 0 |  |  |  |  |
| *Ticinosuchus ferox* | 1 | 0 | 0 | 0 | 0 | 0 | ? | ? | ? | ? | 2 |  |  |  |  |
| *Qianosuchus mixtus* | 1 | 0 | 0 | 0 | 0 | 1 | ? | 0 | 0 | 0 | 2 |  |  |  |  |
| *Arizonasaurus babbitti* | ? | ? | ? | ? | ? | ? | ? | ? | ? | ? | ? |  |  |  |  |
| *Xilousuchus sapingensis* | ? | ? | ? | ? | ? | ? | ? | ? | ? | ? | 2 |  |  |  |  |
| *Poposaurus gracilis* | 1 | 0 | 0 | 0 | ? | 1 | 0 | 0 | 0 | 0/1 | 1 |  |  |  |  |
| *Lotosaurus adentus* | 1 | 0 | 0 | 0 | ? | 1 | ? | 1 | 0 | 0 | 1 |  |  |  |  |
| *Sillosuchus longicervix* | ? | ? | ? | ? | ? | ? | ? | ? | ? | ? | ? |  |  |  |  |
| *Shuvosaurus inexpectatus* | 1 | 0 | 0 | 1 | 1 | 1 | ? | 0 | 0 | ? | 1 |  |  |  |  |
| *Effigia okeeffeae* | 1 | 0 | 0 | 1 | 1 | 1 | ? | 0 | 0 | ? | 1 |  |  |  |  |
| *Prestosuchus* | 1 | 0 | 0 | 0 | 0 | 1 | ? | 1 | 0 | 0 | 2 |  |  |  |  |
| *Saurosuchus galilei* | 1 | 0 | 0 | 0 | 0 | 1 | ? | 1 | 0 | 0 | 2 |  |  |  |  |
| *Batrachotomus kuperferzellensis* | 1 | 0 | 0 | ? | 0 | ? | ? | 1 | 0 | ? | 2 |  |  |  |  |
| *Decuriasuchus quartacolonia* | ? | ? | ? | ? | ? | ? | ? | ? | ? | ? | ? |  |  |  |  |
| *Fasolasuchus tenax* | 1 | ? | ? | ? | ? | ? | ? | ? | ? | ? | ? |  |  |  |  |
| *Rauisuchus triradentes* | ? | ? | ? | ? | ? | ? | ? | ? | ? | ? | ? |  |  |  |  |
| *Postosuchus alisonae* | 1 | 0 | 0 | 1 | 0 | 1 | 0 | 1 | 0 | 1 | 0 |  |  |  |  |
| *Postosuchus kirkpatricki* | 1 | 0 | 0 | 1 | 1 | 1 | ? | 1 | 0 | ? | 0 |  |  |  |  |
| *Polonosuchus silesiacus* | ? | ? | ? | ? | ? | ? | ? | ? | ? | ? | ? |  |  |  |  |
| *Carnufex carolinensis* | ? | ? | ? | ? | ? | ? | ? | ? | ? | ? | ? |  |  |  |  |
| CM 73372 | 1 | 0 | 0 | 1 | 1 | ? | 0 | 1 | 0 | 1 | 0 |  |  |  |  |
| *Redondavenator quayensis* | ? | ? | ? | ? | ? | ? | ? | ? | ? | ? | ? |  |  |  |  |
| *“Hesperosuchus agilis”* | 1 | 0 | 0 | 1 | ? | 1 | ? | 0 | 0 | ? | ? |  |  |  |  |
| *Dromicosuchus grallator* | ? | ? | ? | ? | ? | ? | ? | ? | ? | ? | ? |  |  |  |  |
| *Hesperosuchus agilis* HT | 1 | 0 | ? | ? | ? | ? | ? | 0 | 0 | 2 | ? |  |  |  |  |
| *Sphenosuchus acutus* | ? | 1 | ? | ? | 0 | 1 | ? | ? | ? | ? | ? |  |  |  |  |
| *Terrestrisuchus gracilis* | 1 | 1 | 1 | 1 | 0 | 1 | 1 | 0 | 1 | 1 | 0 |  |  |  |  |
| *Dibothrosuchus elaphros* | ? | ? | ? | ? | ? | ? | ? | ? | ? | ? | ? |  |  |  |  |
| *Litargosuchus leptorhynchus* | 1 | 1 | 0 | ? | ? | ? | ? | 0 | 1 | 2 | ? |  |  |  |  |
| *Kayentasuchus walker* | ? | ? | ? | ? | ? | ? | ? | ? | ? | ? | ? |  |  |  |  |
| *Junggarsuchus sloani* | ? | ? | ? | ? | ? | ? | ? | ? | ? | ? | ? |  |  |  |  |
| *Orthosuchus stormbergi* | 1 | ? | ? | ? | ? | ? | ? | 0 | 0 | 2 | ? |  |  |  |  |
| *Protosuchus richardsoni* | 1 | 1 | 0 | 1 | 0 | 1 | 1 | 0 | 0 | 2 | 0 |  |  |  |  |
| *Protosuchus haughtoni* | ? | ? | ? | ? | ? | ? | ? | ? | ? | ? | ? |  |  |  |  |
| *Alligator mississippiensis* | 1 | 0 | 0 | 1 | 0 | 1 | 1 | 0 | 0 | 2 | 0 |  |  |  |  |
|  |  |  |  |  |  |  |  |  |  |  |  |  |  |  |  |
|  |  |  |  |  |  |  |  |  |  |  |  |  |  |  |  |
